# Exposure Patterns Driving Ebola Transmission in West Africa: A Retrospective Observational Study

**DOI:** 10.1371/journal.pmed.1002170

**Published:** 2016-11-15

**Authors:** International Ebola Response Team, Junerlyn Agua-Agum, Archchun Ariyarajah, Bruce Aylward, Luke Bawo, Pepe Bilivogui, Isobel M. Blake, Richard J. Brennan, Amy Cawthorne, Eilish Cleary, Peter Clement, Roland Conteh, Anne Cori, Foday Dafae, Benjamin Dahl, Jean-Marie Dangou, Boubacar Diallo, Christl A. Donnelly, Ilaria Dorigatti, Christopher Dye, Tim Eckmanns, Mosoka Fallah, Neil M. Ferguson, Lena Fiebig, Christophe Fraser, Tini Garske, Lice Gonzalez, Esther Hamblion, Nuha Hamid, Sara Hersey, Wes Hinsley, Amara Jambei, Thibaut Jombart, David Kargbo, Sakoba Keita, Michael Kinzer, Fred Kuti George, Beatrice Godefroy, Giovanna Gutierrez, Niluka Kannangarage, Harriet L. Mills, Thomas Moller, Sascha Meijers, Yasmine Mohamed, Oliver Morgan, Gemma Nedjati-Gilani, Emily Newton, Pierre Nouvellet, Tolbert Nyenswah, William Perea, Devin Perkins, Steven Riley, Guenael Rodier, Marc Rondy, Maria Sagrado, Camelia Savulescu, Ilana J. Schafer, Dirk Schumacher, Thomas Seyler, Anita Shah, Maria D. Van Kerkhove, C. Samford Wesseh, Zabulon Yoti

**Affiliations:** 1 World Health Organization, Geneva, Switzerland; 2 Ministry of Health, Monrovia, Liberia; 3 Ministry of Health, Conakry, Guinea; 4 MRC Centre for Outbreak Analysis and Modelling, Department of Infectious Disease Epidemiology, School of Public Health, Imperial College London, London, United Kingdom; 5 World Health Organization, Freetown, Sierra Leone; 6 World Health Organization, Monrovia, Liberia; 7 Ministry of Health, Freetown, Sierra Leone; 8 Centers for Disease Control and Prevention, Conakry, Guinea; 9 World Health Organization, Conakry, Guinea; 10 Department for Infectious Disease Epidemiology, Robert Koch Institute, Berlin, Germany; 11 Oxford Big Data Institute, Li Ka Shing Centre for Health Information and Discovery, Nuffield Department of Medicine, University of Oxford, Oxford, United Kingdom; 12 Centers for Disease Control and Prevention, Freetown, Sierra Leone; 13 MRC Integrative Epidemiology Unit, School of Social and Community Medicine, University of Bristol, Bristol, United Kingdom; 14 School of Veterinary Sciences, University of Bristol, Bristol, United Kingdom; 15 European Centre for Disease Prevention and Control, Conakry, Guinea; 16 Epiconcept, Conakry, Guinea; Mahidol-Oxford Tropical Medicine Research Unit, THAILAND

## Abstract

**Background:**

The ongoing West African Ebola epidemic began in December 2013 in Guinea, probably from a single zoonotic introduction. As a result of ineffective initial control efforts, an Ebola outbreak of unprecedented scale emerged. As of 4 May 2015, it had resulted in more than 19,000 probable and confirmed Ebola cases, mainly in Guinea (3,529), Liberia (5,343), and Sierra Leone (10,746). Here, we present analyses of data collected during the outbreak identifying drivers of transmission and highlighting areas where control could be improved.

**Methods and Findings:**

Over 19,000 confirmed and probable Ebola cases were reported in West Africa by 4 May 2015. Individuals with confirmed or probable Ebola (“cases”) were asked if they had exposure to other potential Ebola cases (“potential source contacts”) in a funeral or non-funeral context prior to becoming ill. We performed retrospective analyses of a case line-list, collated from national databases of case investigation forms that have been reported to WHO. These analyses were initially performed to assist WHO’s response during the epidemic, and have been updated for publication.

We analysed data from 3,529 cases in Guinea, 5,343 in Liberia, and 10,746 in Sierra Leone; exposures were reported by 33% of cases. The proportion of cases reporting a funeral exposure decreased over time. We found a positive correlation (*r* = 0.35, *p <* 0.001) between this proportion in a given district for a given month and the within-district transmission intensity, quantified by the estimated reproduction number (*R*). We also found a negative correlation (*r* = −0.37, *p <* 0.001) between *R* and the district proportion of hospitalised cases admitted within ≤4 days of symptom onset. These two proportions were not correlated, suggesting that reduced funeral attendance and faster hospitalisation independently influenced local transmission intensity. We were able to identify 14% of potential source contacts as cases in the case line-list. Linking cases to the contacts who potentially infected them provided information on the transmission network. This revealed a high degree of heterogeneity in inferred transmissions, with only 20% of cases accounting for at least 73% of new infections, a phenomenon often called super-spreading. Multivariable regression models allowed us to identify predictors of being named as a potential source contact. These were similar for funeral and non-funeral contacts: severe symptoms, death, non-hospitalisation, older age, and travelling prior to symptom onset. Non-funeral exposures were strongly peaked around the death of the contact. There was evidence that hospitalisation reduced but did not eliminate onward exposures. We found that Ebola treatment units were better than other health care facilities at preventing exposure from hospitalised and deceased individuals. The principal limitation of our analysis is limited data quality, with cases not being entered into the database, cases not reporting exposures, or data being entered incorrectly (especially dates, and possible misclassifications).

**Conclusions:**

Achieving elimination of Ebola is challenging, partly because of super-spreading. Safe funeral practices and fast hospitalisation contributed to the containment of this Ebola epidemic. Continued real-time data capture, reporting, and analysis are vital to track transmission patterns, inform resource deployment, and thus hasten and maintain elimination of the virus from the human population.

## Abstract 2

In this retrospective observational study, Christophe Fraser and colleagues analyze the exposure patterns associated with Ebola transmission during the 2013-2016 epidemic in West Africa.

## Author Summary

**Why Was This Study Done?:**

- Knowing how and from whom individuals acquire infection can help inform the response to limit the impact of an epidemic; this study presents updated versions of analyses initially performed to assist the international response during the 2013–2016 Ebola epidemic in West Africa.
- Over 19,000 individuals with confirmed or probable Ebola (“cases”) were reported in West Africa by 4 May 2015.
- Cases were asked whether they had exposure to potential Ebola cases (“potential source contacts”) in a funeral or non-funeral context prior to becoming ill.

**What Did the Researchers Do and Find?:**

- We analysed data from 3,529 cases in Guinea, 5,343 in Liberia, and 10,746 in Sierra Leone; exposures were reported by 33% of cases.
- Non-funeral exposures were strongly peaked around the time of death of the contact.
- There was evidence of super-spreading, with only 20% of cases accounting for at least 73% of new infections.

**What Do These Findings Mean?:**

- Safe funeral practices and fast hospitalisation contributed to the containment of this Ebola epidemic.
- The data are highly detailed despite the challenging circumstances in the three countries; however, the analyses were limited by data quality, mostly missing data and incorrect entries.
- In light of viral persistence in reservoirs, it is vital to maintain active surveillance and analysis of Ebola outbreaks to avoid and contain future outbreaks.

## Introduction

**Fig 1:**
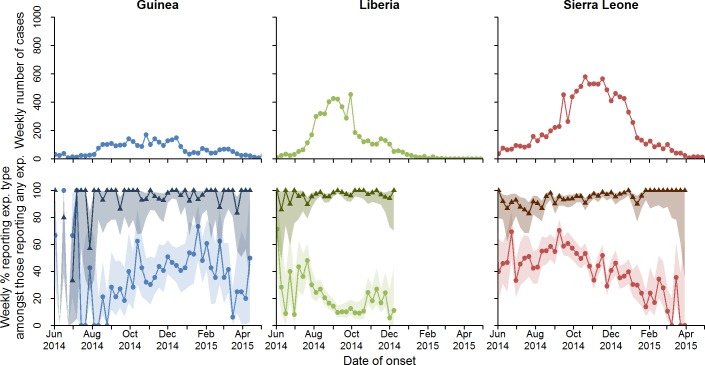
Total number of confirmed and probable cases by week, and percentage who reported funeral and non-funeral exposures. Total number of confirmed and probable cases by week is shown in the top row. Percentage of cases who reported a non-funeral exposure (triangles) or a funeral exposure (circles) is shown in the second row. The shaded regions represent the 95% confidence intervals around the proportions. Note that cases can report more than one exposure, and so percentages need not add to 100%. exp., exposure.

The ongoing West African Ebola epidemic began in December 2013 in Guinea, probably from a single zoonotic introduction [1,2]. As a result of ineffective initial control efforts, an Ebola outbreak of unprecedented scale emerged. As of 4 May 2015, it had resulted in more than 19,000 probable and confirmed Ebola cases, mainly in Guinea (3,529), Liberia (5,343), and Sierra Leone (10,746) (see section 1.3 in S1 Text for WHO case definitions). Control measures for Ebola are well known and based on past experience [3–6]. However, the lack of local experience in handling Ebola outbreaks coupled with severely limited health care resources and poor coordination in the international response led to an initial failure to prevent exponential spread of the outbreak [7,8]. International partners, including the World Health Organization (WHO), emphasised four interventions [9]: (1) prompt identification and isolation of cases, (2) tracing of contacts, (3) safe and dignified burials, and (4) community awareness and social mobilisation. Following several months of intensive efforts to enhance control measures and local community mobilisation, incidence dramatically fell in all three countries [10] (Fig 1, top row).

To quantify the risk factors for transmission, we analysed reported exposure data collected during the epidemic in Guinea, Liberia, and Sierra Leone. We hypothesized that many reported exposures corresponded to transmission events, and that we could therefore discover correlates of transmission and properties of the transmission network by studying available exposure records. Analyses were informed by epidemiological knowledge accrued during previous Ebola outbreaks and by previous expertise in outbreak analysis. The descriptive analyses and association studies were not prespecified, but rather analyses were designed in response to preliminary examination of the accruing data, and in discussion amongst partners in the team. We also hypothesized that if the interventions deployed to interrupt transmission were effective in this epidemic, we should observe an association between the rate of epidemic spread at the district level and the proportion of cases reporting funeral attendance and/or slow hospitalisation. This latter analysis was decided upon before statistical implementation, and was thus hypothesis-driven. We did not have data to assess the effect of contact tracing or community mobilisation.

Data were collated during the outbreak to assist with surveillance and the response. National case databases were shared with WHO and were merged to form a joint line-list (one line of data for each case). The retrospective analyses presented here use data from the three main affected countries (Guinea, Liberia, and Sierra Leone) and were first conducted in real time in September and October 2014 as part of the response to the public health emergency. They were shared with WHO, national authorities, and international agencies working in the three countries and contributed to the planning and monitoring of the control effort.

In summary, the objectives of these analyses are to understand the drivers of local transmission, the timing of transmission events relative to clinical timelines, and the characteristics of the transmission network. These are different from and complement the objectives of our previous papers [11–14]. However, aspects of the process are the same as in those previous analyses: data cleaning, time series of incidence, and method of estimation for the reproduction number. The same exposure data were used to estimate the incubation period and serial interval in previous analyses [11,12]. The dataset analysed is the most comprehensive available on the current epidemic, and represents a unique resource with which to increase our understanding of the outbreak and of Ebola transmission in general. The analysis and presentation of the characteristics of exposures, transmission network, transmission risks, characteristics of potential transmitters and multiple transmitters, health care worker (HCW) exposures, and correlations between epidemic growth and behaviour are all novel.

## Methods

### Ethical Considerations

This paper is based on data collected during surveillance and response activities for Ebola in Guinea, Liberia, and Sierra Leone. The work is based on routine national surveillance for Ebola, for which informed consent is not needed. The surveillance was carried out under WHO Integrated Disease Surveillance and Response guidelines in Africa (the recommended disease notification system). Data were reported to WHO under the obligations of the International Health Regulations. All work was carried out under this legal framework. All information on individual patients has been anonymized for analysis and presentation.

### Data

Information from Ebola cases was collected using a standardised case investigation form [12] and the Epi Info Viral Hemorrhagic Fever Application (https://epiinfovhf.codeplex.com/). The cleaning of the data has been described previously [11,12].

The case investigation form records information on exposures that may have led to the infection of the case. Cases were asked if in the month prior to symptom onset they had had contact with a potential Ebola case and/or had attended a funeral. Cases could report details of up to five exposure events with potential source contacts (up to three exposures with potential source contacts outside of a funeral context, and up to two exposures at funerals of potential source contacts); from Autumn 2014, an abbreviated form was introduced with up to two non-funeral and one funeral exposure (see S2–S6 Texts). Cases reported the name of the person they were exposed to and provided details about their relationship to that person (e.g., family, friend, neighbour) and the nature of the exposure (e.g., touched bodily fluids or shared belongings). All names were anonymized using the SHA-1 hashing algorithm [15]. Here, we use “potential source contact”, “source contact”, or “contact” to describe the person who was named as a potential Ebola source, and “exposure” to describe the potential transmission event that took place between the case and the potential source contact (see Box 1 for terminology). The potential source contact may be alive or dead at the time of the exposure.

**Box 1. Terminology: Cases, Contacts, and Exposures**

- Case: An individual in the line-list with confirmed or probable (CP) Ebola virus disease status using WHO case definitions [16] (see section 1.3 in S1 Text). None of our results changed substantially when analyses were run on confirmed, probable, and suspected cases combined (see section 3 in S1 Text).
- Exposure: An event reported by a case in which the case came into contact with an ill or dead person or attended their funeral. Cases could report more than one exposure.
- Non-funeral exposure: Exposure with an ill person, who may be alive or dead, but not at a funeral. Cases could report up to three non-funeral exposures.
- Funeral exposure: Attendance at a funeral, which could involve touching the corpse. Cases could report up to two funeral exposures.
- Potential source contact, source contact, or contact: The person with whom the exposure was made.
- Matched contact: A potential source contact whom we could identify as a reported case in the line-list.
- Matched exposure: An exposure between a case and his or her matched potential source contact.

Ebola cases are classified as confirmed, probable, or suspected. Here we analysed data from all CP cases unless otherwise stated. In practice, different countries implemented slightly different case definitions; hence, we also performed sensitivity analyses considering confirmed, probable, and suspected cases combined (see section 3 in S1 Text).

### Analysis of Predictors of Naming an Exposure

We assessed whether cases who reported at least one non-funeral or funeral exposure were different from cases who did not report that type of exposure. This allowed us to assess whether cases who report exposures are representative of the line-list. Multivariable logistic regressions were performed using predictors identified as significant (*p <* 0.05) in univariable logistic regressions (see section 1.11 in S1 Text for a list of predictors included in univariable analyses). The most parsimonious yet adequate multivariable model was then identified (using the Akaike information criterion) through backwards stepwise model selection (see final results in Tables b and c in S1 Text).

### Matching Named Potential Source Contacts to the Case Line-List

To identify potential transmission pairs and thus elucidate transmission networks, we examined whether named potential source contacts were themselves Ebola cases. We searched for all named contacts among the names of cases in the line-list to find possible matches. Multiple checks were undertaken to verify the consistency of all matched case-contact pairs according to a set of rules (see section 1.4 and Figure a in S1 Text), in particular comparing dates of symptom onset and exposure.

Importantly, the exposure data analysed here are retrospective and are completely distinct from prospective contact tracing data, where cases are asked who they had contact with *after* they became ill; we do not analyse prospective contact tracing data here, as such data were not available to us, but rather focus on identified potential source contacts.

### Assessing Changes in Infection Control in Health Care Facilities over Time

We hypothesized that the observed decrease in the proportion of HCWs (the positions reported include medical staff and other health-care-facility-based workers) among cases over time (see Figure f in S1 Text) was due to decreased unprotected exposure to patients. To address this issue, we compared the estimated slopes from two linear regression models of (1) the proportion of HCWs among cases over time and (2) the proportion of hospitalised patients over time (see section 1.5 in S1 Text). Note that we define “hospitalisation” as admission to a number of different health care facilities (see Box 2).

**Box 2. Hospitalisation**

We use “hospitalisation” to mean the admission of any patient to a health care facility, as recorded in the case investigation form. This refers to a variety of events in a range of facilities, including

- Ebola treatment centres or units
- Ebola holding centres or units
- Community care centres
- Hospitals
- Health centres, health units, or post-maternal or child health posts (clinics)
- Referral centres

If a case is hospitalised for Ebola, WHO guidelines recommend that the case be isolated. The list above includes a variety of health care facilities where isolation capacity and quality will vary. It should be noted that the meaning of “date of hospitalisation” may be different among cases, depending on how many and which type of facilities they reported visiting in the course of their disease. However, the data do not allow us to distinguish between different meanings of hospitalisation. It should also be noted that not all transfers of patients between different types of health care units may have been recorded in the case investigation form. Data cleaning for hospital type is described in section 1.4 in S1 Text.

### Fitting Distributions to Intervals from Clinical Event to Exposure

Understanding when onward exposure occurred in the clinical course of infection in a primary case could inform the focus of interventions, for instance for early detection of cases or for improving the safety of funerals. We analysed the timing of non-funeral exposure events relative to the following clinical events of the named contacts: onset of symptoms, hospitalisation, and death. Here, we describe the analysis of the time from symptom onset to onward exposure; the analyses for hospitalisation and death were similar. We fitted (by maximum likelihood) a distribution to the observed delays between symptom onset and exposure. In brief (see section 1.8 in S1 Text for more detail), this likelihood accounted for (1) multiple exposures (when multiple exposures were reported, only one was likely to have led to infection, so exposures were weighted accordingly), (2) inaccurate recall of contacts, (3) inaccurate recall of dates of exposures, (4) errors in data entry. In order to address issues 2 to 4, we fitted a mixture of two offset lognormal distributions: the more peaked we regarded as the signal, and the broader we regarded as the noise (see section 1.8 in S1 Text). We then interpreted the signal distribution as our best estimate of actual infection times. Missing delays were imputed by random draws from the observed data.

The analyses described above showed that the non-funeral exposure events were concentrated shortly after the onset of symptoms and around the day of death (see Results). We performed an additional analysis to further explore whether one of these two clinical events was more influential for the timing of onward non-funeral exposure events. We compared the ability to predict (1) the date of death of the source contact based on date of symptom onset of the source contact and the date of exposure reported by the case and (2) the date of symptom onset of the source contact based on date of death of the source contact and date of onward exposure (see Figure o and section 1.9 in S1 Text). These two models assume that the timing of onward exposure is mainly determined by the date of symptom onset (model 1) or by the date of death (model 2).

### Analysis of the Network of Reported Exposures

The transmission network can be described by a network of nodes (cases) linked by directed edges (exposures leading to transmission). In order to characterise the heterogeneities in transmission, we analysed the properties of this network. We looked at separate networks for funeral and non-funeral exposures (see section 1.10 in S1 Text). For each, we analysed the out-degree distribution, i.e., the distribution of the number of cases naming the same matched contact, which can be seen as a proxy for the distribution of the number of secondary cases per index case. Several parametric distributions were fitted to each of the two observed out-degree distributions using maximum likelihood estimation (see section 1.10 in S1 Text for a list of the distributions explored). The Akaike information criterion corrected for finite sample sizes (AICc) [17] was then used to select the best distribution. For the best-fitting distribution, 95% confidence intervals for the distribution parameters were obtained using the likelihood ratio test, and the confidence interval for the corresponding distribution was obtained by numerical sampling.

We derived the variance and the coefficient of variation for the offspring distribution (the distribution of the number of secondary cases infected by each case), based on the assumption that the network of exposures shown in Figure p in S1 Text is a sample of the full transmission network (see section 1.10 in S1 Text).

### Analysis of Predictors of Being Named and Being Named Multiple Times as a Potential Source Contact

To obtain further insight into potential drivers of transmission, we analysed the data to see if there were predictors of being a potential source contact. We performed four analyses: (1) a logistic regression, which identified predictors of being named as a non-funeral contact, one or more times; (2) a logistic regression restricted to cases who died, which identified predictors of being named as a funeral contact, one or more times; (3) a negative binomial regression, which identified predictors of being named multiple times as a non-funeral contact, conditional on being named at least once; and (4) a negative binomial regression restricted to cases who died, which identified predictors of being named multiple times as a funeral contact, conditional on being named at least once. Predictors included in the univariable regressions were as defined in section 1.11 of S1 Text, and the method for identifying the final parsimonious multivariable model is described above. We performed these analyses on confirmed, probable, and suspected contacts who had been named by CP cases. This allowed us to understand the role of suspected contacts in onward transmission compared to CP contacts.

### Correlates of Transmission Intensity

We explored the relationship between district-level (second administrative division) transmission intensity and (1) the proportion of cases reporting funeral attendance amongst those reporting any exposure and (2) the proportion of cases ever hospitalised and the proportion of cases hospitalised ≤4 days (4 days was the median delay from symptom onset to hospitalisation; see Figure m in S1 Text for sensitivity analysis to that threshold). The transmission intensity was quantified by the reproduction number, *R*, which estimates the average number of secondary cases per index case [11,18] (see section 1.6 in S1 Text). We estimated the reproduction number ($R_{m}^{d}$) and the proportion ($p_{m}^{d}$), for every district (*d*) in all three countries over monthly intervals (*m*). ($R_{m}^{d}$ was estimated with the R package “EpiEstim” [19] using district-level incidence, presented in Fig 1 at the country level; see section 1.6 in S1 Text.) The relationship between ($R_{m}^{d}$) and the proportions was explored using linear regressions. As these quantities were estimated from a sample of data with a level of uncertainty, we used a custom linear regression method that accounts for measurement error [20,21]. The method to compute the correlation coefficient was also adapted to account for measurement error (see section 1.7 in S1 Text).

All data cleaning and analyses were performed using *R* software [22].

## Results

### Characteristics of Exposures

Of the 19,618 CP Ebola cases in the line-list, 6,403 (33%) cases reported one or more exposures (Table 1), giving a total of 9,711 reported exposures. Temporal trends in case incidence and reported exposures by country are shown in Fig 1. The proportion of cases reporting exposures varied by country (Table 1); in general, we found that exposure data were less complete for Guinea. Cases reporting exposures were broadly representative of all cases in the line-list (see Tables b and c and Figure c in S1 Text for a comparison of characteristics between cases who reported exposures and those who did not).

**Table 1:** Number of confirmed or probable (CP) cases, exposures, and matched CP-CP contacts and details of the type of exposure and the reported relationship between the case and potential source contact.

Detail	All	Guinea	Liberia	Sierra Leone
***Numbers of cases*, *exposures*, *and matched contacts***				
**Total cases**	19,618	3,529	5,343	10,746
**Cases reporting an exposure**	6,403 (32.6%)	892 (25.3%)	2,078 (38.9%)	3,433 (31.9%)
Only non-funeral	4,183 (65.3%)	571 (64.0%)	1,717 (82.6%)	1,895 (55.2%)
Only funeral	247 (3.9%)	40 (4.5%)	49 (2.4%)	158 (4.6%)
Both	1,973 (30.8%)	281 (31.5%)	312 (15.0%)	1,380 (40.2%)
**Total reported exposures**	9,711	1,366	2,803	5,542
Funeral	2,382 (24.5%)	325 (23.8%)	396 (14.1%)	1,661 (30.0%)
Non-funeral	7,329 (75.5%)	1,041 (76.2%)	2,407 (85.9%)	3,881 (70.0%)
**Total matched exposures**	1,352 (13.9%)	319 (23.4%)	345 (12.3%)	688 (12.4%)
Funeral	243 (18.0%)	68 (21.3%)	24 (7.0%)	151 (21.9%)
Non-funeral	1,109 (82.0%)	251 (78.7%)	321 (93.0%)	537 (78.1%)
**Total number of matched potential source contacts (cases who were named as contacts multiple times are only counted once)**	753	163	237	353
***Details about types of exposures***				
**Funeral, with type of exposure reported**	1,657 (69.6%)	216 (66.5%)	273 (68.9%)	1,168 (70.3%)
Touched corpse	1,071 (64.6%)	154 (71.3%)	167 (61.2%)	750 (64.2%)
Did not touch corpse	586 (35.4%)	62 (28.7%)	106 (38.8%)	418 (35.8%)
**Non-funeral, with type of exposure reported**	2,461 (33.6%)	102 (9.8%)	1,430 (59.4%)	929 (23.9%)
Belongings	1,379 (56.0%)	30 (29.4%)	757 (52.9%)	592 (63.7%)
Bodily fluids	1,318 (53.6%)	35 (34.3%)	711 (49.7%)	572 (61.6%)
Within same household	937 (38.1%)	31 (30.4%)	492 (34.4%)	414 (44.6%)
Direct physical	2,136 (86.8%)	72 (70.6%)	1,262 (88.3%)	802 (86.3%)
**Funeral, with the relationship reported**	1,952 (81.9%)	53 (16.3%)	360 (90.9%)	1,539 (92.7%)
Close family	1,079 (55.3%)	34 (64.2%)	194 (53.9%)	851 (55.3%)
Extended family	550 (28.2%)	11 (20.8%)	96 (26.7%)	443 (28.8%)
Friend	121 (6.2%)	1 (1.9%)	50 (13.9%)	70 (4.5%)
Neighbour	154 (7.9%)	1 (1.9%)	9 (2.5%)	144 (9.4%)
Health care	6 (0.3%)	0 (0%)	0 (0%)	6 (0.4%)
Other	42 (2.2%)	6 (11.3%)	11 (3.1%)	25 (1.6%)
**Non-funeral, with the relationship reported**	6,105 (83.3%)	242 (23.2%)	2,249 (93.4%)	3,614 (93.1%)
Close family	3,610 (59.1%)	148 (61.2%)	1,336 (59.4%)	2,126 (58.8%)
Extended family	1,435 (23.5%)	48 (19.8%)	483 (21.5%)	904 (25.0%)
Friend	335 (5.5%)	10 (4.1%)	182 (8.1%)	143 (4.0%)
Neighbour	431 (7.1%)	12 (5.0%)	113 (5.0%)	306 (8.5%)
Health care	103 (1.7%)	6 (2.5%)	43 (1.9%)	54 (1.5%)
Other	191 (3.1%)	18 (7.4%)	92 (4.1%)	81 (2.2%)

Not all cases who reported funeral exposure explicitly reported whether they had touched the corpse. Cases who reported non-funeral exposure could report multiple types of exposure: belongings—“touched or shared the linens, clothes, or dishes/eating utensils of the case [contact]”; bodily fluids—“touched the body fluids of the case (blood, vomit, saliva, urine, feces)”; in same household—“slept, ate, or spent time in the same household or room as the case”; direct physical—“had direct physical contact with the body of the case”. Relationship was not reported for every exposure. We grouped reported relationships into classes: “close family” is defined as siblings, marital, and parent-child relationships; other family members are considered “extended family”; “neighbour” is defined as tenants, lodgers, landlords, and neighbours; “health care” is defined as HCW-patient relationships and caregivers, or any reference to a patient; “other” includes traditional healers, contacts through religious practice, and transport contacts. Type of exposure and relationship type are illustrated graphically in Figure d in S1 Text.

Exposure at funerals is a known risk factor for Ebola infection [23–25], and 25% of cases who reported any exposure in the current outbreak reported exposures at funerals. Most cases (89%) reporting a funeral exposure also reported one or more non-funeral exposures.

Cases were asked to provide details on the nature of their exposures and their relationships with the contacts, which are shown in Table 1. Overall, 87% of exposures occurred between family members (of those where the relationship was reported). Up until the introduction of the new case investigation form (see S2–S6 Texts), non-funeral exposures were recorded as one or more types (e.g., an exposure could simultaneously involve shared belongings and exposure to bodily fluids). Of those non-funeral exposures for which the type of exposure was reported, over 90% were reported to involve contact with bodily fluids and/or direct physical contact, and 38% were reported as occurring in a household (defined as having slept, eaten, or spent time in the same household or room as the contact). These patterns did not change significantly over time (see Figure e in S1 Text). For funeral exposures, cases were asked whether they had touched the corpse. Of those giving a response, 65% reported having touched the corpse, with this proportion being greatest for Guinea (71%) and least for Liberia (61%). This proportion declined significantly after October 2014 (*p <* 0.001; see Figure h in S1 Text), most notably in Sierra Leone.

### Matching Named Potential Source Contacts to the Case Line-List

We were able to identify 14% of potential source contacts as cases in the line-list. Our ability to match contacts did not vary substantially over time (see Figure i in S1 Text) or geographically (see Figure c in S1 Text).

### Characteristics of the Transmission Network

**Fig 2:**
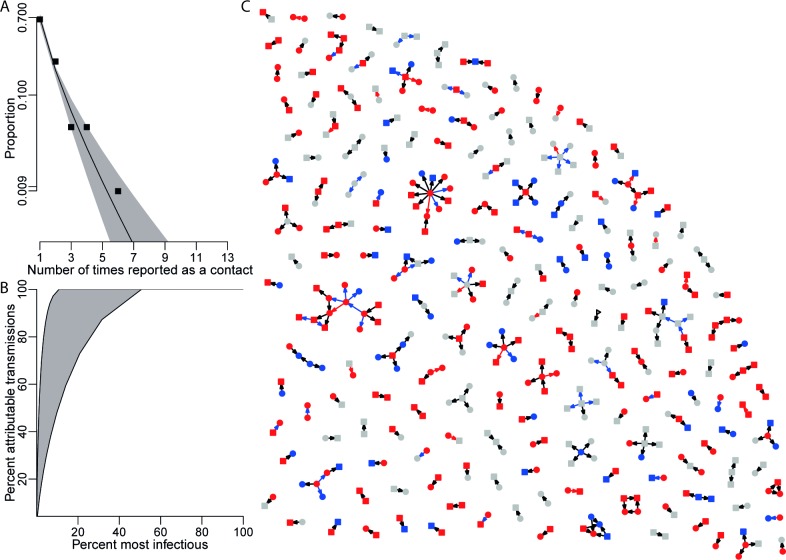
Contact network. (A) The out-degree distribution of the network of exposures shows the probability that a named contact is named as an exposure by a certain number of cases. The squares represent the observed data. The black line shows the maximum likelihood logarithmic distribution, with 95% confidence interval in grey. (B) The cumulative density function of the derived offspring distribution. The two black lines (edges) show two scenarios (see Figure q in S1 Text for details). (C) A sample of the network of all cases (see Figure p in S1 Text for full network) and the contacts they have named as having been exposed to. Individuals (cases and contacts) are shown as nodes, and exposures as directed arrows from contacts to cases. Arrows are red for funeral exposures, black for non-funeral exposures, and blue for multiple exposures involving both non-funeral and funeral exposures. Square nodes are males, round nodes females, and triangles unknown. Red nodes are cases who have died, blue nodes are cases who have survived, and grey nodes are cases with no recorded outcome.

The analysis of matched exposures provides information on the transmission network underpinning the Ebola epidemic (Fig 2). There was evidence of modest assortativity in exposure patterns by sex (see Table i in S1 Text) and country-specific patterns by age (see Table j in S1 Text). The most important statistic characterising the transmission network is the out-degree distribution, the number of times each person was named as a contact by other Ebola cases. The observed out-degree distribution was best fitted by a logarithmic probability distribution for both funeral and non-funeral contacts (Fig 2A; see sections 1.10 and 2.8 in S1 Text). Since the network is not known in its entirety, but only through a sample of cases and their matched contacts, additional assumptions were needed to infer the true offspring distribution (the distribution of the number of secondary cases infected by each case) from the observed out-degree distribution. Under the assumption that the matched exposures are representative of the underlying transmission network, we find high to extreme variability in the offspring distribution (Fig 2B; see section 1.10 in S1 Text). The estimated coefficient of variation for the offspring distribution ranges from 1.6 to 5.6 depending on assumptions (see section 1.10 in S1 Text). This implies that 5% of cases accounted for at least 30% of all new infections and that 20% of cases accounted for at least 73% of new infections (Fig 2B; see Figure q in S1 Text), a phenomenon termed super-spreading [26]. Super-spreading was found to affect both non-funeral and funeral transmissions equally (see Tables f and g in S1 Text).

### Transmission Risk Relative to Onset of Symptoms and Death

**Fig 3:**
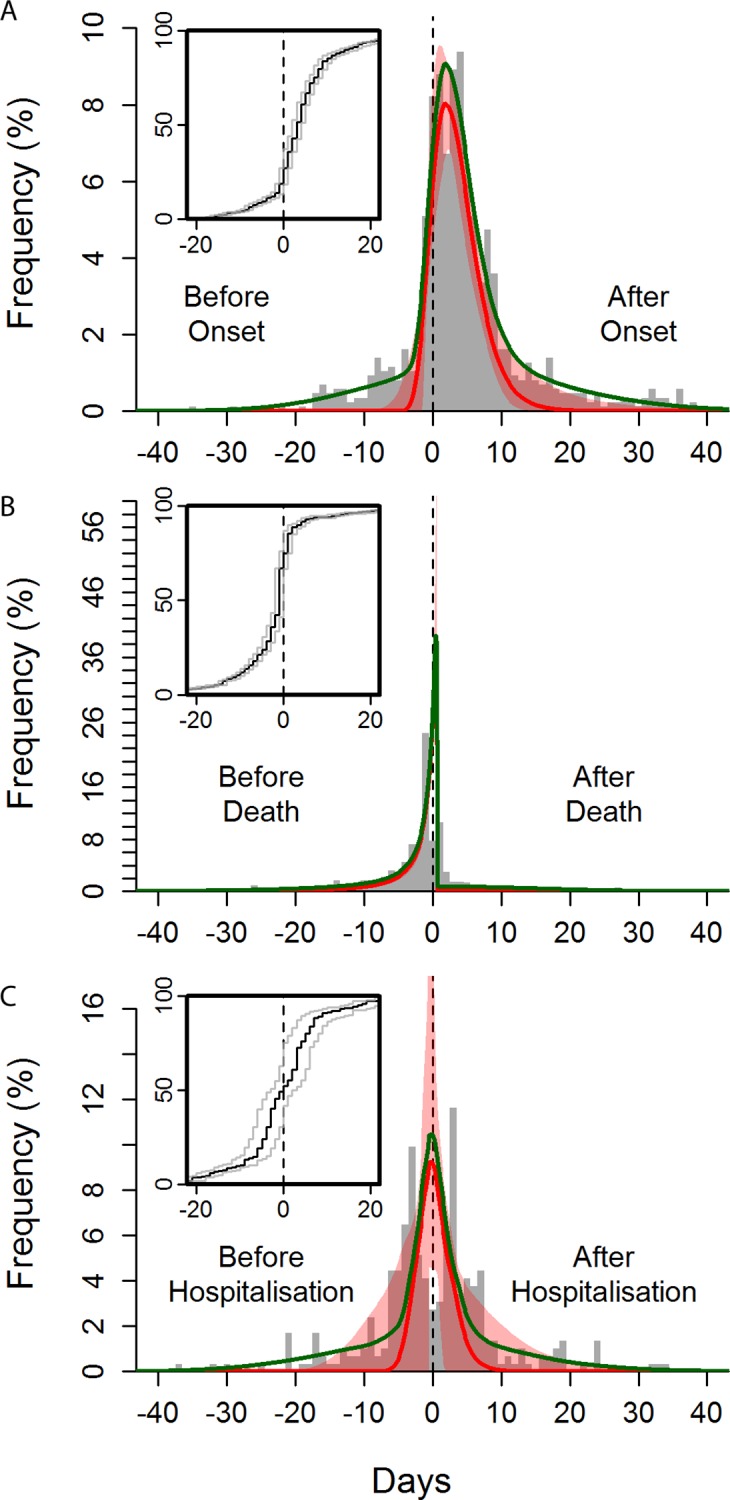
Observed and fitted distribution of reported time to non-funeral exposure from symptom onset, hospitalisation, and death of the contact. Time from symptom onset (A), death (B), and hospitalisation (C) of the contact to time of exposure. The green curves show the overall best fits, and the red curves show the best fits for the “signal” distribution (all obtained by maximum likelihood). The red-shaded areas indicate the 95% confidence intervals of the fitted “signal” distribution. The histogram shows a random set of exposure midpoints (in some instances, only a start or an end date of exposure is recorded; in those instances, the missing date is numerically imputed). Note that the fitting procedure is not performed on the midpoints but fully incorporates the exposure window (see section 1.8 in S1 Text). The inset panels are the observed cumulative distribution functions for the midpoint (black line) and start and end (grey lines) of the exposures.

One key potential determinant of the risk of onward transmission is the stage of progression of clinical illness. The risk of transmission was found to increase over time since symptom onset (Fig 3A), peaking 2 days after onset, with some exposures estimated to have occurred more than 2 weeks after onset. Our model estimates a small probability of transmission before symptom onset; however, we do not regard this as strong evidence for pre-symptomatic transmission: all reported dates are prone to recall bias, but it is likely that the date of symptom onset is more uncertain than dates of hospitalisation and death, as it is subjective, so individuals may interpret symptom onset differently.

Transmission events from non-funeral exposures were estimated to be strongly peaked on the day of and the day after the death of the contact (Fig 3B). In all, 44% of non-funeral exposures to potential source contacts who died were estimated to occur on or after the date of death of the contact. Furthermore, individuals who died were more likely to be named as non-funeral contacts (univariable odds ratio [OR] = 2.10 [95% CI: 1.68, 2.66]).

We sought to determine whether date of death or date of symptom onset was the stronger predictor of transmission risk. We found that date of death was the stronger predictor, indicating that the timing of death is the strongest determinant of when transmission occurs (see Figure o in S1 Text).

### Exposures Occurring in Hospitals and after Hospitalisation

Hospitalisation (see Box 2 for definition of hospitalisation, which in this context is isolation from the community by admission to any health care facility) aims to improve the clinical outcomes of patients with Ebola virus disease, and also to reduce onward exposures. We found that hospitalised cases (who were hospitalised at any stage of their infection) had a reduced risk of being named as a non-funeral source contact (univariable OR = 0.78 [95% CI: 0.66, 0.92]) and as a funeral source contact (OR = 0.43 [95% CI: 0.29, 0.63]) compared to cases never hospitalised. This indicates that hospitalisation reduced but did not eliminate onward exposures. The impact of hospitalisation on limiting onward exposure was stronger in Guinea than in Liberia (significantly so for non-funeral exposures, see Tables m and n in S1 Text). The protective effect of hospitalisation on exposure in Sierra Leone was not statistically significant. There was no significant increase in the effect of hospitalisation over time for any of the three countries (see Tables m and n in S1 Text). Previously, we examined the timing of transmission risk relative to symptom onset and death. Using the same method to characterise the timing of exposure events relative to hospitalisation, we found that a substantial proportion (42% [95% CI: 12%, 54%]) of non-funeral exposures from hospitalised source contacts occurred after their hospitalisation (Fig 3C).

Our definition of hospitalisation covers a broad range of health care facility types (Box 2), each with varying levels of infection control [27]. In particular, as part of the international response to the epidemic, an unprecedented number of Ebola treatment units (ETUs) were built to care for patients. The previous analysis considered all types of hospitalisation together. When we disaggregated hospitalisation by ETU versus other facilities, we found that ETUs were better than other health care facilities at preventing exposure from hospitalised cases and at providing safer management of corpses. In ETUs, the observed proportion of non-funeral exposures from hospitalised cases occurring after hospitalisation was significantly lower than in non-ETU facilities (*p <* 0.001, see Figure n in S1 Text). Moreover, hospitalisation in an ETU reduced the risk of being named as a funeral contact compared with non-hospitalised cases (univariable OR = 0.30 [95% CI: 0.16, 0.50]) more than hospitalisation in a non-ETU facility did (OR = 0.72 [95% CI: 0.39, 1.23]; also see multivariable analyses in Table l in S1 Text and the next section). Although for non-funeral contacts univariable ORs were not significant, a multivariable analysis indicated similar effects (see Table k in S1 Text).

Overall, our analyses demonstrate that hospitalisation reduced infectious exposures to cases, but that the effectiveness of hospital isolation measures varied by country and facility type, with substantial scope for improvements.

### Epidemiological Characteristics of Potential Transmitters

To further explore the characteristics of individuals who were named as potential source contacts (and therefore potential transmitters), we also performed systematic multivariable logistic regressions on the risk of being named as a potential source contact (see Tables k and l in S1 Text). Based on the final multivariable model, individuals significantly more likely to be named as a non-funeral contact were those who were more severely affected at presentation (with fever [versus no fever], OR = 1.52 [95% CI: 1.20, 1.95]; with bleeding [versus no bleeding], OR = 1.40 [95% CI: 1.07, 1.81]; unconscious [versus conscious], OR = 1.98 [95% CI: 1.26, 3.03]); those who died (versus survivors, OR = 2.04 [95% CI: 1.50, 2.79]; confirmed cases (versus suspected cases, OR = 2.45 [95% CI: 2.01, 2.99]); and probable cases (versus suspected cases, OR = 1.91, [95% CI: 1.51, 2.41]). In addition, cases who were never hospitalised (versus cases hospitalised in an ETU) were more likely to be named as a non-funeral contact (OR = 1.61 [95% CI: 1.30, 2.00]). Finally, adults (≥16 versus <16 years old, OR = 1.87 [95% CI: 1.52, 2.34]) and those who reported travelling outside of their village before they became ill (versus not travelling, OR = 1.73 [95% CI: 1.35, 2.18]) were also more likely to be named as a non-funeral potential source contact.

Similar predictors were found for individuals being named as funeral contacts: more severely affected cases (fever versus no fever, OR = 1.81 [95% CI: 1.08, 3.18]; respiratory versus no respiratory symptoms, OR = 1.65 [95% CI: 1.09, 2.54]), adults (≥16 versus <16 years old, OR = 2.44 [95% CI: 1.47, 4.36]), cases not hospitalised (versus hospitalised in an ETU, OR = 5.56 [95% CI: 2.94, 11.11]), those who reported travelling before they became ill (versus not travelling, OR = 2.47 [95% CI: 1.50, 3.90]), confirmed cases (versus suspected cases, OR = 1.98 [95% CI: 1.28, 3.11]), probable cases (versus suspected cases, OR = 2.03 [95% CI: 1.21, 3.42]), and those who were reported as Ebola cases after death (versus before death, OR = 1.64 [95% CI: 1.12, 2.40]).

### Epidemiological Factors Associated with Being a Multiple Transmitter

To determine whether there were any factors associated with being multiply identified as a potential source contact, we conducted a multivariable negative binomial regression to explore predictors of the number of times cases were named as non-funeral and funeral source contacts, conditional on being named at least once (see Tables p and q in S1 Text). Cases with reported haemorrhagic symptoms were found to be named as non-funeral contacts 1.67 times more often than cases not reporting these symptoms (95% CI: 1.07, 2.64). Cases hospitalised in an ETU were found to be named as non-funeral contacts 0.50 as many times as non-hospitalised cases (95% CI: 0.33, 0.73). Also, HCWs were found to be named as non-funeral contacts 0.48 as many times as non-HCWs (95% CI: 0.23, 0.96). Travel outside one’s village of residence prior to becoming ill was the only significant predictor for a case to be named multiple times as a funeral contact (relative number of times being named compared to cases who did not report travelling was on average 4.41 [95% CI: 2.01, 10.55]).

### No Difference in Exposure Risk between the Sexes

Sex did not appear as an important predictor of exposure risk in any of the analyses that we performed.

### Exposures Involving Health Care Workers

HCWs have been at particularly high risk of infection, especially during the early part of the epidemic [28]. HCWs reported that 49% of their non-funeral exposures were with other HCWs or patients, versus 0.6% reported by non-HCWs. Consequently, HCWs reported a lower percentage of exposures with family members (44%) compared to non-HCWs (91%; see Figure f in S1 Text). This demonstrates that HCWs were often infected whilst working. Consistent with these data, the matched contacts reported by HCWs were more likely to have evidence of hospitalisation at some point in their illness than the matched contacts of non-HCWs (OR = 2.91 [95% CI: 1.57, 5.81]).

In the absence of data on the total number of HCWs working in each of the three countries over time, we could not estimate the relative risk of infection of HCWs compared to non-HCWs. However, several indicators point to falling risk through time. Since June 2014, the proportion of HCWs among all cases has decreased over time (see Figure f in S1 Text). The rate of decrease in this proportion (estimated monthly relative decrease since June: 21% [95% CI: 18%, 24%]) was faster than that seen in the incidence of hospitalised cases (estimated monthly relative decrease: 12% [95% CI: 10%, 14%]; see also Figures f and g in S1 Text), suggesting an improvement in infection control measures and increased awareness of Ebola among HCWs over time.

### Population Correlates of Epidemic Intensity

The analyses presented so far describe individual risks of infection. We next explored, in a hypothesis-driven manner, whether local epidemic transmission intensity was correlated with local population measures of presumed heightened risk of infection.

**Fig 4:**
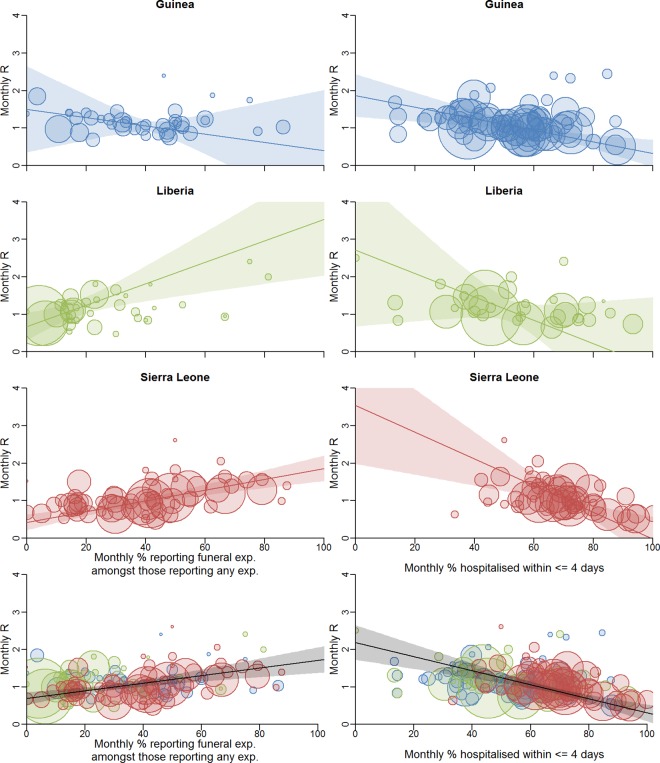
Correlation between local transmission intensity and local population measures of presumed heightened risk of infection. Correlation between local transmission intensity and proportion of cases reporting funeral exposures among those reporting any exposure (left) and proportion of cases hospitalised within ≤4 days of symptom onset among those hospitalised (right). The scatter plots show these monthly proportions against monthly estimated reproduction numbers *R* (method as previous [11]) for the supplemented incidence (i.e., incidence based on two data sources including the line-list, see [12]). Each point is a district-month. Trend lines are shown with 95% confidence intervals (shaded areas). We use a weighted regression method that takes account of the uncertainties in the data [20]. The area of each circle is proportional to the weight of that point (see section 1.7 in S1 Text). In the bottom row, the black trend line is for the whole dataset. See Figures j and l and Table d in S1 Text for details, including trend line parameters. exp., exposure.

We first examined whether the proportion of cases reporting funeral exposures in each district by month was correlated with the estimated reproduction number *R* in that district at that time. Having accounted for uncertainty in the estimates of both quantities [20] (see section 1.7 in S1 Text), we found a positive correlation for Liberia (*r* ^2^ *=* 0.30, *p =* 0.013) and Sierra Leone (*r* ^2^ *=* 0.23, *p <* 0.001) and no significant correlation for Guinea (*r* ^2^ *=* 0.001, *p =* 0.667) (Fig 4; see Figure j and Table d in S1 Text). This analysis suggests that across the three countries an absolute reduction of 10% in the proportion reporting funeral exposures would lead to an absolute reduction of 0.10 in *R* (95% CI: 0.05, 0.15; *p <* 0.001). In particular, reducing the proportion reporting funeral exposures to less than 29% (95% CI: 21%, 38%) would be sufficient to reduce *R* below 1, the threshold value for achieving control of the epidemic. However, this result varies by country, and is an association that does not prove causation, since reductions in funeral exposures could have been accompanied by changes in other control measures and other effects of community mobilisation. In that case, the measure is a surrogate for the total effect of interventions that correlate with reduced funeral exposures.

Another factor potentially influencing changes in transmission intensity is how promptly cases are hospitalised and isolated. We found no association between the proportion of cases that were hospitalised and the estimated district-level reproduction number *R* (see Figure k in S1 Text), likely because the line-list predominantly includes cases who have been hospitalised. However, we found a negative association between the estimated district-level *R* and the proportion of hospitalised cases admitted within ≤4 days after onset of symptoms in Liberia (*r* ^2^ = 0.22, *p =* 0.039) and Sierra Leone (*r* ^2^ = 0.32, *p <* 0.001). No significant correlation was found in Guinea (*r* ^2^ = 0.02, *p =* 0.377) (Fig 4; see Figures l and m in S1 Text). This analysis quantifies the impact that earlier hospitalisation has in reducing transmission. Across all three countries we estimate that increasing the proportion of cases hospitalised within ≤4 days of symptom onset by 10% would reduce *R* by 0.19 (95% CI: 0.12, 0.25; *p <* 0.001). In particular, if 64% (95% CI: 59%, 68%) of hospitalised cases are admitted within ≤4 days, *R* is predicted to be <1. Again, the effect varied by country.

We found no correlation between the two predictors we analysed, i.e., the proportion of cases reporting funeral exposure and the proportion of cases hospitalised within ≤4 d of symptom onset (naïve *r* ^2^ *=* 0.001, *p =* 0.711; see section 2.5 in S1 Text). This shows that both factors are independently associated with the level of transmission, at least in Liberia and Sierra Leone, and, together, they explain a substantial proportion (approximately 55%) of the variance in epidemic transmission intensity observed in these two countries.

## Discussion

The analyses in this paper draw on 9,711 exposures to potential sources of infection, to provide a quantitative basis for understanding Ebola epidemiology and to validate the choice of interventions used to combat Ebola in the current and future outbreaks. These analyses were initially performed in September/October 2014 as part of the WHO Ebola response during the epidemic and have been updated to be shared with the scientific community.

The current Ebola outbreak has been of unprecedented scale, occurring across three countries of diverse cultures with varying levels of urbanisation. Our analysis provides a unique overview of Ebola transmission that complements detailed investigations that have been performed in localised settings in this and previous outbreaks [29–33]. Small studies may allow more detailed reconstruction of transmission and identification of risk factors for infection by comparing cases and non-cases, which is not feasible at a larger scale. However, analyses such as identifying district-level correlates for transmission intensity and determining when exposures occur with respect to clinical events are only possible with this unique large-scale dataset.

Our analysis confirms that exposure to Ebola cases at funerals is an important amplifier of Ebola transmission, in line with a study focused in Sierra Leone [32]. The significant correlation we found between the district-level reported frequency of funeral exposures and local transmission intensity provides support for the policy emphasis on safe and dignified burials. This effect was not replicated in Guinea, which suggests we have less understanding of drivers of transmission in that country.

Our results also highlight the importance of exposure to individuals who are dead or dying from Ebola outside the funeral context, as has been observed in past outbreaks [4,23–25,34,35]. Cases who died were more likely to be named as contacts than those who survived, with most transmission occurring within a few days on either side of the reported date of death—coincident with the timing of peak viral load [34,36]. This reinforces existing evidence [4,23–25,34,35] that exposures to individuals who are dead or dying from Ebola contribute to transmission, even where those exposures occur outside the specific context of funerals. As in past outbreaks [3,4,23,25], and consistent with other analyses of the current outbreak [29], such exposures have most often been between close family members, perhaps explaining why most such contacts have occurred in the household.

Early hospitalisation in facilities with the ability to isolate patients effectively—a key element of control efforts for Ebola—is clearly a priority to reduce community transmission. Investigations of the initial phase of this epidemic, as well as previous outbreaks, have highlighted the potential role of within-hospital transmission before or shortly after Ebola is identified as the causative agent [37,38]. Here we found that hospitalisation, defined broadly as anything from visiting a clinic to admission to an ETU, reduced transmission risk, showing that prompt hospitalisation may be an effective intervention as long as appropriate control measures are applied. However, hospitalisation did not eliminate transmission risk, indicating that improvements in infection control are needed in many health care settings. It should be noted that the meaning of date of hospitalisation may be different among cases, depending on how many and which types of facilities they visited in the course of their disease, and furthermore that there is potential for misclassification in the recording or interpretation of information on hospitalisation. In particular, while the health care facility of first admission was commonly reported, patient transfers to other facilities may not have always been recorded. Thus, there may be ambiguity about the type of health care facility a case was in at a particular time. Such missing information reduces our ability to resolve differences between types of facilities in their effectiveness at implementing case isolation, which could lead to underestimation of differences between facility types in isolation effectiveness. However, our results are broadly consistent with analyses of the outbreak in Conakry [29]. Half of reported non-funeral exposures where the matched contact was eventually hospitalised occurred after the reported date of hospitalisation. These results underscore the importance of further improving infection control in all health care facilities, but particularly in non-ETUs. However, in contexts where hospital bed capacity is insufficient or infection control in health care settings is imperfect, greater consideration might be given to measures to reduce within-family exposure—for example, via education and/or providing a home protection kit with hand disinfectant, protective equipment, and clear guidance for those caring for sick family members.

Another striking feature of the epidemic revealed by our analysis is the high level of heterogeneity in the number of times a case is named as a potential source contact. Such heterogeneity has been observed for some other emerging infectious disease epidemics [26,39–41], in particular the Middle East respiratory syndrome coronavirus outbreaks [42]. In principle, understanding the drivers of this heterogeneity might allow for the design of targeted interventions. However, our analysis found very few epidemiological predictors for being named as a source contact multiple times, suggesting that simple demographic characteristics are unlikely to pinpoint those most at risk of super-spreading. Heterogeneity in transmission, particularly when associated with transmission in close communities, implies that epidemic trajectories may be difficult to predict at a local level [43,44]. Local flare-ups are possible when case numbers are low and declining. Continued vigilance during the ongoing declining phase of the epidemic is essential.

The data we have analysed have several limitations. Not all Ebola cases are recorded in the national databases shared with WHO. Moreover, data on exposures were reported by only approximately one-third of cases. The remainder of cases may not have had the chance to report exposures or may have been unable to recall any. Additionally, data collection teams and methods varied by country, district, and hospital facility, meaning observational or collection bias may have affected the data. All data (e.g., symptoms at presentation) were either self-reported or reported by friends/family or inferred by the interviewer and may therefore suffer from subjectivity and recall bias. In particular, there may be biases in the exposures reported—for example, cases may recall funerals or exposures to family in more detail or have different perceptions of what constitutes exposure. Recall and data entry errors (e.g., spelling mistakes, mistyped dates, and misclassification) and missing data may have affected our results, for example, they limited our ability to match the contacts named in the reported exposures. We accounted for this in our analyses as much as possible by discarding inconsistent data, imputing missing data, or explicitly accounting for noise. However, some biases may remain. Performing these quantitative analyses on such a large scale was only possible due to the enormous commitment of Ebola responders in the region throughout the epidemic, which is particularly remarkable given the very challenging circumstances in the affected countries.

In generalising our findings, we need to recognise the relatively unique nature of this crisis. Measures that were promoted, including emphasising bed capacity, safe funerals, behaviour change, and community mobilisation, followed the recognition that targeted case finding and contact tracing could not be scaled to meet the exponentially growing burden during the early phase of the epidemic. However, these latter measures, supported by continued community mobilisation, should be prioritised during new Ebola outbreaks and during the end phase of the current epidemic, when capacity can meet need.

Our analyses provide a quantitative basis for prevention measures against the spread of Ebola, but also highlight the challenges faced in the field. A compassionate response protocol needs to acknowledge that, as we found, most reported potential transmissions occurred between family members close to the time of death of the case. This provides evidence that ring vaccination methods, such as those trialled in Guinea [45], may be an effective way of delivering vaccines against Ebola in the context of limited supply or in hard-to-reach populations. Hospitalisation was found to be protective, but left substantial room for improvement. The association between reported funeral attendance and district-level epidemic trends highlights the continued need for improving access to and acceptability of safe burials of Ebola cases. More robust associations could be detected using case-control studies, but these are hard to coordinate during an emergency situation. In their absence, the analyses reported here have already provided key insights into the drivers of the epidemic. Heterogeneities in transmission made the road to Ebola elimination likely to be marked by episodic flare-ups [46,47]. Ebola is controllable using the simple measures that have already been implemented in this outbreak, but complacency as case numbers decline could prolong the epidemic for months. Continued real-time data capture, reporting, and analysis are vital to track transmission patterns, inform resource deployment, and thus hasten elimination of the virus from the human population.

## Supporting Information

S1 STROBE Checklist (DOCX)

S1 Text Additional methods and results. (DOCX)

S2 Text Long version of the case investigation form used in Sierra Leone and Liberia. (PDF)

S3 Text Long version of the case investigation form used in Guinea. (PDF)

S4 Text Short version of the case investigation form used in Sierra Leone. (PDF)

S5 Text Short version of the case investigation form used in Liberia. (PDF)

S6 Text Short version of the case investigation form used in Guinea. (PDF)

## Abbreviations

CP: confirmed or probable
ETU: Ebola treatment unit
HCW: health care worker
OR: odds ratio
