# Supplementary material for: Exposure Patterns Driving Ebola Transmission in West Africa: A Retrospective Observational Study
Source: PLoS Med. 2016 Nov 15;13(11):e1002170. doi: 10.1371/journal.pmed.1002170 (PMC5112802; doi:10.1371/journal.pmed.1002170)
Supplement: S1 STROBE Checklist — (DOCX) [file pmed.1002170.s001.docx]

STROBE Statement—checklist of items that should be included in reports of observational studies

|  | **Item No.** | **Recommendation** | **Relevant text from manuscript** |
| --- | --- | --- | --- |
| **Title and abstract** | 1 | (a) Indicate the study’s design with a commonly used term in the title or the abstract | ***Title:*** *“Exposure patterns driving Ebola transmission in West Africa,* ***a retrospective observational study****”* |
|  |  | (b) Provide in the abstract an informative and balanced summary of what was done and what was found | ***Abstract, Methods and Findings:***  *“Over 19,000 Confirmed and Probable Ebola cases were reported in West Africa by 4th May 2015. Cases were asked if they had exposure to potential Ebola cases (‘potential source contacts’) in a funeral or non-funeral context prior to becoming ill. We performed retrospective analyses of a case line-list, collated from national - databases of case report forms that have been reported to the WHO. These analyses were initially performed to assist WHO’s response during the epidemic, and have been updated for publication.*  *We analysed data from 3,529 cases in Guinea, 5,343 in Liberia and 10,746 in Sierra Leone; exposures were reported by 33% of cases. The proportion of cases reporting a funeral exposure decreased over time. We found a positive correlation (r=0.35, p<0.001) between this proportion in a district for a given month and the within-district transmission intensity, quantified by the estimated reproduction number (R). We also found a negative correlation (r=-0.37, p<0.001) between R and the district’s proportion of hospitalised cases admitted within ≤4 days of symptoms onset. These two proportions were not correlated, suggesting that reduced funeral attendances and faster hospitalisation independently influenced local transmission intensity. We were able to identify 14% of potential source contacts as cases in the case line-list. Linking cases to the contacts who potentially infected them provided information on the transmission network. This revealed a high degree of heterogeneity in inferred transmissions, with only 20% of cases accounting for at least 73% of new infections, a phenomenon often called super-spreading. Multivariable regression models allowed us to identify predictors of being named as a potential source contact. These were similar for funeral and non-funeral contacts: severe symptoms, death, non-hospitalization, older age and travelling prior to onset. Non-funeral exposures were strongly peaked around the death of the contact, with very few (<1%) exposures occurring after hospitalization. We found that Ebola Treatment Units were better than other healthcare facilities at preventing exposure from hospitalised and deceased individuals. The principal limitation of our analysis is limited data quality, with cases either not being entered into the database, not reporting exposures, or data being entered incorrectly (especially dates, and possible misclassifications).”* |
| **Introduction** |  |  |  |
| Background/rationale | 2 | Explain the scientific background and rationale for the investigation being reported | ***Introduction, Opening paragraphs:***  *“The ongoing West African Ebola epidemic began in December 2013 in Guinea, probably from a single zoonotic introduction [1, 2]. As a result of ineffective initial control efforts, an Ebola outbreak of unprecedented scale emerged. As of 4th May 2015, it had resulted in more than 19,000 Probable and Confirmed Ebola cases, mainly in Guinea (3,529), Liberia (5,343) and Sierra Leone (10,746) (see section 1.3 in S1 Text for WHO case definitions). Control measures for Ebola are well known and based on past experience [3-6]. However, the lack of local experience in handling Ebola outbreaks coupled with severely limited health care resources and poor coordination in the international response led to an initial failure to prevent exponential spread of the outbreak [7, 8]. International partners, including the World Health Organization (WHO), emphasised four interventions [9]: 1. Prompt identification and isolation of cases; 2. Tracing of contacts; 3. Safe and dignified burials; and 4. Community awareness and social mobilization. Following several months of intensive efforts to enhance control measures and local community mobilization, incidence dramatically fell in all three countries [10].”* |
| Objectives | 3 | State specific objectives, including any prespecified hypotheses | ***Introduction, following paragraphs:***  *“To quantify the risk factors for transmission, we analysed reported exposure data collected during the epidemic in Guinea, Liberia, and Sierra Leone. We hypothesized that many reported exposures corresponded to transmission events, and that we could therefore discover correlates of transmission and properties of the transmission network by studying available exposure records. Analyses were informed by epidemiological knowledge accrued during previous Ebola outbreaks and by previous expertise in outbreak analysis. The descriptive analyses and association studies were not pre-specified, but rather analyses were designed in response to preliminary examination of the accruing data, and in discussion amongst partners in the team. We also hypothesised that if the interventions deployed to interrupt transmission were effective in this epidemic, we should observe an association between the rate of epidemic spread at the district level and the proportion of cases reporting funeral attendance and/or slow hospitalisation. These latter analyses were decided upon before statistical implementation, and were thus hypothesis-driven. We did not have data to assess the effect of contact tracing or community mobilization.”* |
| **Methods** |  |  |  |
| Study design | 4 | Present key elements of study design early in the paper. | All methods used are presented in sequence in the **Methods section** of the paper. We have made every effort to provide all information for reproducibility and for the intent of analyses to be clear, and provided many additional details in supplementary file **S1_text.doc** |
| Setting | 5 | Describe the setting, locations, and relevant dates, including periods of recruitment, exposure, follow-up, and data collection. | The information on setting, dates, and details of data recorded is presented in **table 1** and **figure 1**, and **supplementary table a**. |
| Participants | 6 | (a) Cohort study—Give the eligibility criteria, and the sources and methods of selection of participants. Describe methods of follow-up.  Case-control study—Give the eligibility criteria, and the sources and methods of case ascertainment and control selection. Give the rationale for the choice of cases and controls Cross-sectional study—Give the eligibility criteria, and the sources and methods of selection of participants. | **Not applicable:** This is an observational study of all reported cases during this outbreak. |
|  |  | (b) Cohort study—For matched studies, give matching criteria and number of exposed and unexposed  Case-control study—For matched studies, give matching criteria and the number of controls per case | **Not applicable:** This is an observational study of all reported cases during this outbreak. |
| Variables | 7 | Clearly define all outcomes, exposures, predictors, potential confounders, and effect modifiers. Give diagnostic criteria, if applicable. | Diagnostic criteria for cases are clearly defined, in **Introduction, First paragraph.**  *“As of 4^th^ May 2015, it had resulted in more than 19,000 Probable and Confirmed Ebola cases, mainly in Guinea (3,529), Liberia (5,343) and Sierra Leone (10,746) (see section 1.3 in S1 Text for WHO case definitions).”* |
| Data sources/measurement | 8 | For each variable of interest, give sources of data and details of methods of assessment (measurement). Describe comparability of assessment methods if there is more than one group. | **Materials and Methods. Data.**  *Information from Ebola cases are collected using a standardized case investigation form [12] and the Epi Info VHF application (*[*https://epiinfovhf.codeplex.com/*](https://epiinfovhf.codeplex.com/)*). Cleaning of the data has been described previously [11, 12].*  **Introduction**  *“Data was collated during the outbreak to assist with surveillance and the response. National case databases were shared with the WHO and merged to form a joint line-list (one line of data for each case). The retrospective analyses presented here use data from the three main affected countries (Guinea, Liberia and Sierra Leone) and were first conducted in real-time in September and October 2014 as part of the response to the public health emergency. They were shared with WHO, national authorities and international agencies working in the three countries and contributed to the planning and monitoring of the control effort.”*  All data were sourced from the merged line-list. Differences between countries are described in the paper. |
| Bias | 9 | Describe any efforts to address potential sources of bias. | **Materials and Methods. Data.**  One potential source of bias is the case definition.  *“Ebola cases are classified as Confirmed, Probable or Suspected. Here we analysed data from all Confirmed and Probable cases unless otherwise stated. In practice, different countries implemented slightly different case definitions, hence we also performed sensitivity analyses considering Confirmed, Probable and Suspected cases combined (see section 3 in S1 Text).”* |
| Study size | 10 | Explain how the study size was arrived at | **Not applicable:** This is an observational study of all reported cases during this outbreak. |
| Quantitative variables | 11 | Explain how quantitative variables were handled in the analyses. If applicable, describe which variables groupings were chosen and why. | **Supplement S1_Text.doc**  *“The following predictors were included in this univariate analysis: case definition (Confirmed, Probable); sex; age (<16yrs, ≥16 yrs, based on common epidemiological definition of adulthood); whether individuals were hospitalized; the date of report (Dec13-May14, Jun14-Nov14, Dec14-May15, Missing, chosen to represent early, peak and declining phases of the epidemic, respectively); the delays that individuals experienced between onset to hospitalization (≥ 4 days or <4 days, chosen as 4 days was the median delay), onset to death (≥ 6 days or <6 days, chosen as 6 days was the median delay), onset to discharge (≥ 16 days or <16 days, chosen as 16 days was the median delay) and report to death (≥ 0 days or <0 days, chosen to distinguish cases reported before or after death); whether individuals were health care workers (HCWs); the country database they belonged to; the final clinical outcome for the case (dead, alive, unknown); whether the case reported to have travelled outside their village / town recently; whether they reported having attended a funeral (and touched a corpse) and the following recorded symptoms that the case had experienced at the time of completion of the Case Report Form: fever, diarrhea, vomiting (+- blood), signs of bleeding (unexplained, gums, at injection site, nose, stool, vaginal, skin, urine or other signs of bleeding), unconsciousness, confusion, respiratory illness (chest pain, cough (+- blood), difficulty breathing and hiccups), and other (fatigue, anorexia, abdominal pain, muscle pain, joint pain, headache, difficulty swallowing, jaundice, conjunctivitis, rash, pain in eyes and sore throat). Information was available on the occupation of 41% cases but the number of cases in each category was too small to assess whether an occupation type was a significant predictor, apart from HCW status.* *Missing values were treated as separate predictors, since missing data may be informative about how cases were detected.”* |
| Statistical methods | 12 | (a) Describe all statistical methods, including those used to control for confounding | All details are provided.  **Supplement S1_Text.doc**  As is standard, univariate predictors were included in multivariate regressions to help control for confounding. |
|  |  | (b) Describe any methods used to examine subgroups and interactions | **Not applicable** |
|  |  | (c) Explain how missing data were addressed | **Supplement S1_Text.doc**  *“Missing values were treated as separate predictors, since missing data may be informative about how cases were detected. “* |
|  |  | (d) Cohort study—If applicable, explain how loss to follow-up was addressed  Case-control study—If applicable, explain how matching of cases and controls was addressed Cross-sectional study—If applicable, describe analytical methods taking account of sampling strategy | **Not applicable** |
|  |  | (e) Describe any sensitivity analyses | A sensitivity analysis to case definition was carried out. Other minor sensitivity analyses are described throughout the text. |
| Results |  |  |  |
| Participants | 13 | (a) Report numbers of individuals at each stage of study—eg numbers potentially eligible, examined for eligibility, confirmed eligible, included in the study, completing follow-up, and analysed | The information on setting, dates, and details of data recorded is presented in **table 1** and **figure 1**, and supplementary **table a**. All data were included in analyses. |
|  |  | (b) Give reasons for non-participation at each stage | **Not applicable** |
|  |  | (c) Consider use of a flow diagram | We decided this would not clarify the analyses presented here, since all patients were included. |
| Descriptive data | 14 | (a) Give characteristics of study participants (eg demographic, clinical, social) and information on  exposures and potential confounders | Study participants have been described in two previous publications (**References 11 and 12**). In the interests of space, these details were not reproduced in this paper. |
|  |  | (b) Indicate number of participants with missing data for each variable of interest | In the interests of space, we did not feel it practicable to report these here given how many tables this would represent, however we would include these in results available on request (see point 16 below). |
|  |  | (c) Cohort study—Summarise follow-up time (eg, average and total amount) | **Not applicable.** |
| Outcome data | 15 | Cohort study—Report numbers of outcome events or summary measures over time | We are not sure how this is applicable to this observational study of an outbreak, but we have reported the numbers of exposures of different types that were analysed. |
|  |  | Case-control study—Report numbers in each exposure category, or summary measures of exposure |  |
|  |  | Cross-sectional study—Report numbers of outcome events or summary measures |  |
| Main results | 16 | (a) Give unadjusted estimates and, if applicable, confounder-adjusted estimates and their precision (eg, 95% confidence interval). Make clear which confounders were adjusted for and why they were included. | In the interests of space, we did not present all univariable and multivariable regression results. These are supplemental results, and we felt that including dozens of pages of results would not aid comprehension or reproducibility. We state:  **Supplement S1_text.doc**  *“Table b: Predictors of being a named non-funeral contact (logistic regression) multivariable analysis: All Countries. The most parsimonious yet adequate model (determined by the AIC, see methods section) is presented. Covariates that do not contribute to this model are not presented.”*  **Univariable regression results for this and subsequent regressions are available on request.** |
|  |  | (b) Report category boundaries when continuous variables were categorized | See 11 above. |
|  |  | (c) If relevant, consider translating estimates of relative risk into absolute risk for a meaningful time period | **Not applicable.** |
| Other analyses | 17 | Report other analyses done—eg analyses of subgroups and interactions, and sensitivity analyses | **Not applicable.** |
| Discussion |  |  |  |
| Key results | 18 | Summarise key results with reference to study objectives | **Discussion, part 1**  *“Our analysis confirms that exposure at funerals of cases is an important amplifier of Ebola transmission, in line with a study focused in Sierra Leone [32]. The significant correlation we found between the district-level reported frequency of funeral exposures and local transmission intensity provides support for the policy emphasis on safe and dignified burials. This effect was not replicated in Guinea, which suggests we have less understanding of drivers of transmission in that country.*  *Our results also highlight the importance of exposure to dead or dying Ebola cases outside the funeral context, as has been observed in past outbreaks [4, 23-25, 34, 35]. Cases that died were more likely to be named as contacts than those who survived, with most transmission occurring within a few days either side of the reported date of death – coincident with the timing of peak viral load [34, 36]. This reinforces existing evidence [4, 23-25, 34, 35] that exposures to dead or dying cases contribute to transmission, even where those exposures occur outside the specific context of funerals. As in past outbreaks [3, 4, 23, 25], and consistent with other analyses of the current outbreak [29], such exposures have most often been between close family members, perhaps explaining why most such contacts have occurred in the household.*  *Early hospitalization in facilities with the ability to isolate patients effectively – a key element of control efforts for Ebola – is clearly a priority to reduce community transmission. Investigations of the initial phase of this epidemic, as well as previous outbreaks, have highlighted the potential role of within hospital transmission before or shortly after Ebola is identified as the causative agent [37, 38]. Here we found that hospitalization, defined broadly as anything from visiting a clinic to admission to an ETU, reduced transmission risk, showing prompt hospitalization may be an effective intervention as long as appropriate control measures are applied. However, hospitalization did not eliminate transmission risk, indicating that improvements in infection control are needed in many health care settings. It should be noted that the meaning of date of hospitalization may be different among cases, depending on how many and which type of facilities they visited in the course of their disease, and furthermore that there is potential for misclassification in the recording or interpretation of information on hospitalization. In particular, while the healthcare facility of first admission was commonly reported, patient transfers to other facilities may not have always been recorded. Thus there may be ambiguity about the type of healthcare facility a case was in at a particular time. Such missing information reduces our ability to resolve differences between types of facility in their effectiveness at implementing case isolation, which could lead to underestimation of differences between facilities types in isolation effectiveness. However our results are broadly consistent with analyses of the outbreak in Conakry [29]. Half of reported non-funeral exposures where the matched contact was eventually hospitalized occurred after the reported date of hospitalization. These results underscore the importance of further improving infection control in all health care facilities, but particularly in non-ETUs. However, in contexts where hospital bed capacity is insufficient or infection control in health care settings is imperfect, greater consideration might be given to measures to reduce within-family exposure – e.g. via education and/or providing a home protection kit with hand disinfectant, protective equipment and clear guidance for those caring for sick family members.*  *Another striking feature of the epidemic revealed by our analysis is the high level of heterogeneity in the number of times a case is named as a potential source contact. Such heterogeneity has been observed for some other emerging infectious disease epidemics [26, 39-41], in particular the Middle East Respiratory Syndrome Coronavirus (MERS-CoV) outbreaks [42]. In principle, understanding the drivers of this heterogeneity might allow for the design of targeted interventions. However, our analysis found very few epidemiological predictors for being named multiple times, suggesting that simple demographic characteristics are unlikely to pinpoint those most at risk of super-spreading. Heterogeneity in transmission, particularly when allied with transmission in close communities, implies that epidemic trajectories may be difficult to predict at a local level [43, 44]. Local flare-ups are possible when case numbers are low and declining. Continued vigilance during the ongoing declining phase of the epidemic is essential.*  *The data we have analysed have several limitations. Not all Ebola cases are recorded in national databases communicated to the WHO. Moreover, data on exposures was only reported by approximately one third of cases. The remainder of cases may not have had the chance to report exposures or alternatively may have been unable to recall any. Additionally, data collection teams and methods varied by country, district and hospital facility, meaning observational or collection bias may have affected the data. All data (e.g. symptoms at presentation) were either self-reported or reported by friends/family or inferred by the interviewer and may therefore suffer from subjectivity and recall bias. In particular there may be biases in the exposures reported – for example, cases may recall funerals or exposures to family in more detail or have different perceptions of what constitutes exposure. Recall and data entry errors (e.g. spelling mistakes, mistyped dates and misclassification) and missing data may have affected our results, e.g. they limited our ability to match the contacts named in the reported exposure. We accounted for this in our analyses as much as possible by discarding inconsistent data, imputing missing data, or by explicitly accounting for noise. However, some biases may remain. One needs to recognize that performing these quantitative analyses on such a large scale was only possible due to the enormous commitment of Ebola responders in the region throughout the epidemic, which is particularly remarkable given the very challenging circumstances in the affected countries.*  *In generalizing our findings, we need to recognize the relatively unique nature of this crisis. Measures that were promoted, including emphasising bed capacity, safe funerals, behaviour change and community mobilization, followed the recognition that targeted case finding and contact tracing could not be scaled to meet the exponentially growing burden during the early phase of the epidemic. However, these latter measures, supported by continued community mobilization, should be prioritised during new Ebola outbreaks, and during the end phase of the current epidemic, when capacity can meet need.*  *Our analyses provide a quantitative basis for prevention measures against the spread of Ebola, but also highlight the challenges faced in the field. A compassionate response protocol needs to acknowledge that, as we found, most reported potential transmissions occurred between family members close to the time of death of the case. This provides evidence that ring vaccination methods, such as those trialled in Guinea [45] may be an effective way of delivering vaccines against Ebola in the context of limited supply or in hard-to-reach populations. Hospitalization was found to be protective, but left substantial room for improvement. The association between reported funeral attendance and district-level epidemic trends highlights the continued need for improving access to and acceptability of safe burials of Ebola cases. More robust associations could be detected using case-control studies, but these are hard to coordinate during an emergency situation, and in their absence the analyses reported here have already provided key insights into the drivers of the epidemic. Heterogeneities in transmission indicate that the road to elimination of Ebola may be marked by episodic flare-ups [46, 47]. Ebola is controllable using the simple measures which have already been implemented in this outbreak, but complacency as case numbers decline could prolong the epidemic for months. Continued real-time data capture, reporting, and analysis are vital to track transmission patterns, inform resource deployment, and thus hasten elimination of the virus from the human population.”* |
| Limitations | 19 | Discuss limitations of the study, taking into account sources of potential bias or imprecision. Discuss both direction and magnitude of any potential bias. | **Discussion, part 2**  *“The data we have analysed have several limitations. Not all Ebola cases are recorded in national databases communicated to the WHO. Moreover, data on exposures was only reported by approximately one third of cases. The remainder of cases may not have had the chance to report exposures or alternatively may have been unable to recall any. Additionally, data collection teams and methods varied by country, district and hospital facility, meaning observational or collection bias may have affected the data. All data (e.g. symptoms at presentation) were either self-reported or reported by friends/family or inferred by the interviewer and may therefore suffer from subjectivity and recall bias. In particular there may be biases in the exposures reported – for example, cases may recall funerals or exposures to family in more detail or have different perceptions of what constitutes exposure. Recall and data entry errors (e.g. spelling mistakes, mistyped dates and misclassification) and missing data may have affected our results, e.g. they limited our ability to match the contacts named in the reported exposure. We accounted for this in our analyses as much as possible by discarding inconsistent data, imputing missing data, or by explicitly accounting for noise. However, some biases may remain. One needs to recognize that performing these quantitative analyses on such a large scale was only possible due to the enormous commitment of Ebola responders in the region throughout the epidemic, which is particularly remarkable given the very challenging circumstances in the affected countries.*  *In generalizing our findings, we need to recognize the relatively unique nature of this crisis. Measures that were promoted, including emphasising bed capacity, safe funerals, behaviour change and community mobilization, followed the recognition that targeted case finding and contact tracing could not be scaled to meet the exponentially growing burden during the early phase of the epidemic. However, these latter measures, supported by continued community mobilization, should be prioritised during new Ebola outbreaks, and during the end phase of the current epidemic, when capacity can meet need.“* |
| Interpretation | 20 | Give a cautious overall interpretation of results considering objectives, limitations, multiplicity of analyses, results from similar studies, and other relevant evidence | **Discussion, part 3**  *“Our analyses provide a quantitative basis for prevention measures against the spread of Ebola, but also highlight the challenges faced in the field. A compassionate response protocol needs to acknowledge that, as we found, most reported potential transmissions occurred between family members close to the time of death of the case. This provides evidence that ring vaccination methods, such as those trialled in Guinea [45] may be an effective way of delivering vaccines against Ebola in the context of limited supply or in hard-to-reach populations. Hospitalization was found to be protective, but left substantial room for improvement. The association between reported funeral attendance and district-level epidemic trends highlights the continued need for improving access to and acceptability of safe burials of Ebola cases. More robust associations could be detected using case-control studies, but these are hard to coordinate during an emergency situation, and in their absence the analyses reported here have already provided key insights into the drivers of the epidemic. Heterogeneities in transmission indicate that the road to elimination of Ebola may be marked by episodic flare-ups [46, 47]. Ebola is controllable using the simple measures which have already been implemented in this outbreak, but complacency as case numbers decline could prolong the epidemic for months. Continued real-time data capture, reporting, and analysis are vital to track transmission patterns, inform resource deployment, and thus hasten elimination of the virus from the human population.”* |
| Generalisability | 21 | Discuss the generalisability (external validity) of the study results | **Discussion part 5**  *“One needs to recognize that performing these quantitative analyses on such a large scale was only possible due to the enormous commitment of Ebola responders in the region throughout the epidemic, which is particularly remarkable given the very challenging circumstances in the affected countries.*  *In generalizing our findings, we need to recognize the relatively unique nature of this crisis. Measures that were promoted, including emphasising bed capacity, safe funerals, behaviour change and community mobilization, followed the recognition that targeted case finding and contact tracing could not be scaled to meet the exponentially growing burden during the early phase of the epidemic. However, these latter measures, supported by continued community mobilization, should be prioritised during new Ebola outbreaks, and during the end phase of the current epidemic, when capacity can meet need.”* |
| Funding |  |  |  |
| Other information | 22 | Give the source of funding and the role of the funders for the present study and, if applicable, for the original study on which the present article is based | *Supported by the Medical Research Council, the Bill and Melinda Gates Foundation, the Models of Infectious Disease Agent Study of the National Institute of General Medical Sciences (National Institutes of Health), the National Institute for Health Research Health Protection Research Unit for Modelling Methodology, the European Union PREDEMICS consortium, Wellcome Trust, and Fogarty International Center.* |
