# Supplementary material for: Exposure Patterns Driving Ebola Transmission in West Africa: A Retrospective Observational Study
Source: PLoS Med. 2016 Nov 15;13(11):e1002170. doi: 10.1371/journal.pmed.1002170 (PMC5112802; doi:10.1371/journal.pmed.1002170)
Supplement: S1 Text — (DOCX) [file pmed.1002170.s002.docx]

# S1 Text: Exposure patterns driving Ebola transmission in West Africa, a retrospective observational study

The International Ebola Response team

**Table of Contents**

S1 Text: Exposure patterns driving Ebola transmission in West Africa, a retrospective observational study 1

1. Materials and Methods 3

1.1. Country specific dates of data 3

1.2. Case investigation form 3

1.3. WHO Ebola case definitions 4

1.4. Data Processing 5

1.5. Comparing the decreases in the proportions of HCWs and hospitalized cases over time 9

1.6. Estimation of the reproduction number by district 9

1.7. Correlates for transmission intensity 10

1.8. Fitting distributions to intervals from clinical event to exposure 13

1.9. Relative importance of onset and death in the timing of exposure events 15

1.10. Analysis of the network of reported exposures 16

1.11. List of possible predictors of naming an exposure, or being named and being named multiple times as a contact 19

2. Additional results 21

2.1. Number of reported exposures 21

2.2. Representativeness of the exposure data 22

2.3. Patterns in reported exposures 26

2.4. Decreases in the proportions of HCWs and hospitalized cases over time 30

2.5. District-level transmission intensity and relationship to funeral exposure and hospitalizations 31

2.6. Timing of exposure events relative to the clinical timelines of the contact 37

2.7. Relative importance of onset and death in the timing of exposure events 39

2.8. Analysis of the network of reported exposures 40

2.9. Case-contact pairs by age and sex 42

2.10. Predictors of being a named contact 44

2.11. Predictors of being a multiply named contact 48

3. Sensitivity Analysis for Case Definition 50

3.1. Numbers of CPS cases and exposures 50

3.2. Main text figures, for the subset of data with Confirmed, Probable or Suspected (CPS) case definition 51

4. References 56

# Materials and Methods

## Country specific dates of data

The line-list we use was dated 4^th^ May 2015 for Liberia and Sierra Leone and 27^th^ April 2015 for Guinea. Based on the analysis of delays between symptom onset and reporting of a case into the line-list, we considered the line-list to be reliable only up to 5th April 2015 for Sierra Leone, 19^th^ April 2015 for Guinea and 28^th^ December 2014 for Liberia. In Liberia, the reporting system changed and data from Liberia has been unreliable since late 2014. Note that to better capture incidence trends up to early May in the three countries, we also used supplemented incidence data [[1](#_ENREF_1)] (shown in Fig 1, top) for estimation of the reproduction number.

## Case investigation form

Data on exposures were collected using VHF case investigation forms; we have included these as separate files. We present five versions of the form, and have highlighted in red boxes the sections particularly relating to exposure reports:

(1) The English version of a Viral Haemorrhagic Fever (VHF) Case Investigation form. This was used throughout most of 2014.

(2) The above, in French (used in Guinea). Note that the wording

(3) A shorter revised version of the VHF form (in English) which was introduced into Sierra Leone from late Autumn 2014.

(4) A shorter revised version of the VHF form (in English) which was introduced into Liberia from late Autumn 2014.

(5) A shorter version of the VHF form (in French) which was introduced into Guinea in late 2014.

## WHO Ebola case definitions

Ebola cases are classified as Confirmed, Probable or Suspected with the following definitions recommended by the WHO [[2](#_ENREF_2)]:

- *Confirmed*: Any Suspected or probably cases with a positive laboratory result.
- *Probable*: Any Suspected case evaluated by a clinician OR: Any deceased Suspected case (where it has not been possible to collect specimens for laboratory confirmation) having an epidemiological link with a Confirmed case
- *Suspected*: Any person, alive or dead, suffering or having suffered from a sudden onset of high fever and having had contact with: a Suspected, Probable or Confirmed Ebola; or a dead or sick animal (for Ebola) OR: any person with sudden onset of high fever and at least three of compatible symptoms OR: any person with inexplicable bleeding OR: any sudden, inexplicable death

Note that cases can be reclassified over time.

Different case classifications were used in different countries and at different times. To avoid potential biases, we applied our analyses to all Confirmed and Probable cases, and analysed Confirmed, Probable and Suspected cases as a sensitivity analysis.

## Data Processing

This section describes the data cleaning and processing we performed, in particular to match the named contacts to cases in the WHO line-list.

### Matching – identifying named contacts in the line-list

All data were anonymized through encryption of all fields containing names. Names were cleaned before encryption by removing prefixes (e.g. Dr), extra punctuation, accents and whitespace, and all names were converted to lower case.

Matching was done using full name (clean encrypted surname and first name); if a unique match was found in the line-list it was accepted automatically. A considerable challenge was that names of individuals were not unique and for many named contacts several possible candidates were found (sometimes as many as >10 options). Names were sometimes misspelt or had several possible spellings, which our cleaning did not address. Contacts with misspelt names were not matched automatically.

Up to October 14^th^ 2014, all possible candidates for a given exposure were examined manually, and, if possible, one candidate was chosen based on closest acceptable match in terms of location, type of contact, timing of onset of symptoms, date of death and age and relationship of contacts. Candidates were only chosen if the match was unambiguous; otherwise the contact was not matched. After this date, manual matching was only performed on named contacts with just two potential candidates.

After the matching process we further cleaned the dates to correct for possible data entry errors in the month (see below). Once the matching process was complete, all matches were checked for consistency (see below). **Only pairs where both case and probably source contact were confirmed or probable (CP-CP pairs) were used in the main analysis, whilst confirmed, probable or suspected (CPS-CPS) pairs were used in a sensitivity analysis.**

### Adjusting the exposure dates

After the matching process we further cleaned the dates to correct for possible data entry errors in the month. We defined Delay1 as the interval between the reported dates of symptom onset of the contact and exposure of the case. Delay2 was defined as the interval between the reported dates of exposure and symptom onset of the case. For pairs for which both symptom onset dates and an exposure date were reported, we adjusted the exposure dates as follows:

- If Delay1 < 0 and Delay2 ≥ 30 days, we moved the exposure date forward one month.
- If Delay2 < 0 and Delay1 ≥ 30 days, we moved the exposure date back one month.

### When only considering confirmed and probable pairs, only 2 (0.2% of matched CP-CP pairs) have their exposure date changed following these checks. For confirmed, probable and suspected case-contact pairs, 39 pairs have their exposure date changed (2.5% of matched CPS-CPS pairs).

### Consistency checks on matched contacts

Once the matching process was complete, all matches were checked for consistency according to a set of rules. The rules we implemented were a careful balance between sensitivity and specificity: we allowed for some flexibility due to recall bias in dates, but we would rather exclude matches than have the chance of retaining an incorrect match, particularly when a large amount of data was missing. The rules were as follows:

*General Rules:*

1. No self-referencing was allowed.
2. Symptom onset of the contact must be before the death of the case.
3. If there are no reported onset, death, or hospitalization dates for either the case or the contact then the cleaned district field [[3](#_ENREF_3)] of the two individuals must be the same or adjacent to each other. (Note that here we use “district” to refer to the level of prefecture in Guinea, county in Liberia and district in Sierra Leone. Also, we aggregated the “districts” Ratoma, Dixinn, Kaloum, Matam and Matoto to Conakry and aggregated Freetown and Western Rural into Western Area because the districts within these two areas were not specified by some cases).
4. If the exposure is reported as non-funeral with a dead individual or as a funeral exposure the contact cannot have “Alive” as their final status.
5. If the case and contact are reported as a parent-offspring relationship, then their age difference must be at least 12 years.
6. If the case and contact are reported as a grandparent-grandchild relationship, then their age difference must be at least 24 years.
7. If contact and case are in different countries and the type of contact included “in same household”, then the match is deleted.

*Funeral exposure rules:*

1. The funeral of the contact must occur less than 7 days before the exposure. If the funeral date is missing, the date of death of the contact must have occurred a maximum of 14 days before exposure.
2. The date of symptom onset of the contact must be before the exposure date.

*Date rules (if symptom onset of case is reported) (see Figure a in S1 Text):*

1. The date of symptom onset of the contact must be within 42 days before and less than 7 days after the date of symptom onset of the case (Rule A in the figure).
2. If the date of symptom onset of the contact was missing, we used the date of hospitalization and if missing, then the date of death of the contact. If the date of hospitalization is used, it must be within 42 days before and less than 27 days after the exposure (Rule B in the figure). This interval was based on the 97.5 quantile of the hospitalization-to-death distribution plus 7 days, at the end of 2014. If date of death is used, it must be within 42 days before and less than 35 days after exposure (Rule C in the figure). This interval was based on the 97.5 quantile delay of the onset-to-death distribution plus 7 days, at the end of 2014.

*Date rules (if symptom onset of case is unknown) (see Figure a in S1 Text):*

1. For non-funeral exposure: the death of the contact must be less than 7 days before the exposure occurred (Rule D in the figure).
2. For non-funeral exposure: if the date of death of contact is missing, the hospitalization date of the contact must be a maximum of 27 days before exposure (Rule E in the figure). This interval was based on the 97.5 quantile of the hospitalization-to-death distribution plus 7 days, at the end of 2014.
3. For non-funeral exposure: if the date of death and the date of hospitalization of contact is missing, symptom onset of the contact must be a maximum of 35 days before exposure (Rule F in the figure). This interval was based on the 97.5 quantile of the onset-to-death distribution plus 7 days, at the end of 2014.
4. For funeral exposures: the funeral date of the contact must be no more than 7 days before the exposure and must not be after (Rule G in the figure).
5. For funeral exposures, rules 1-3 were applied, however the lower interval was 7 days earlier than for non-funeral exposures (Rules H-J in the figure).


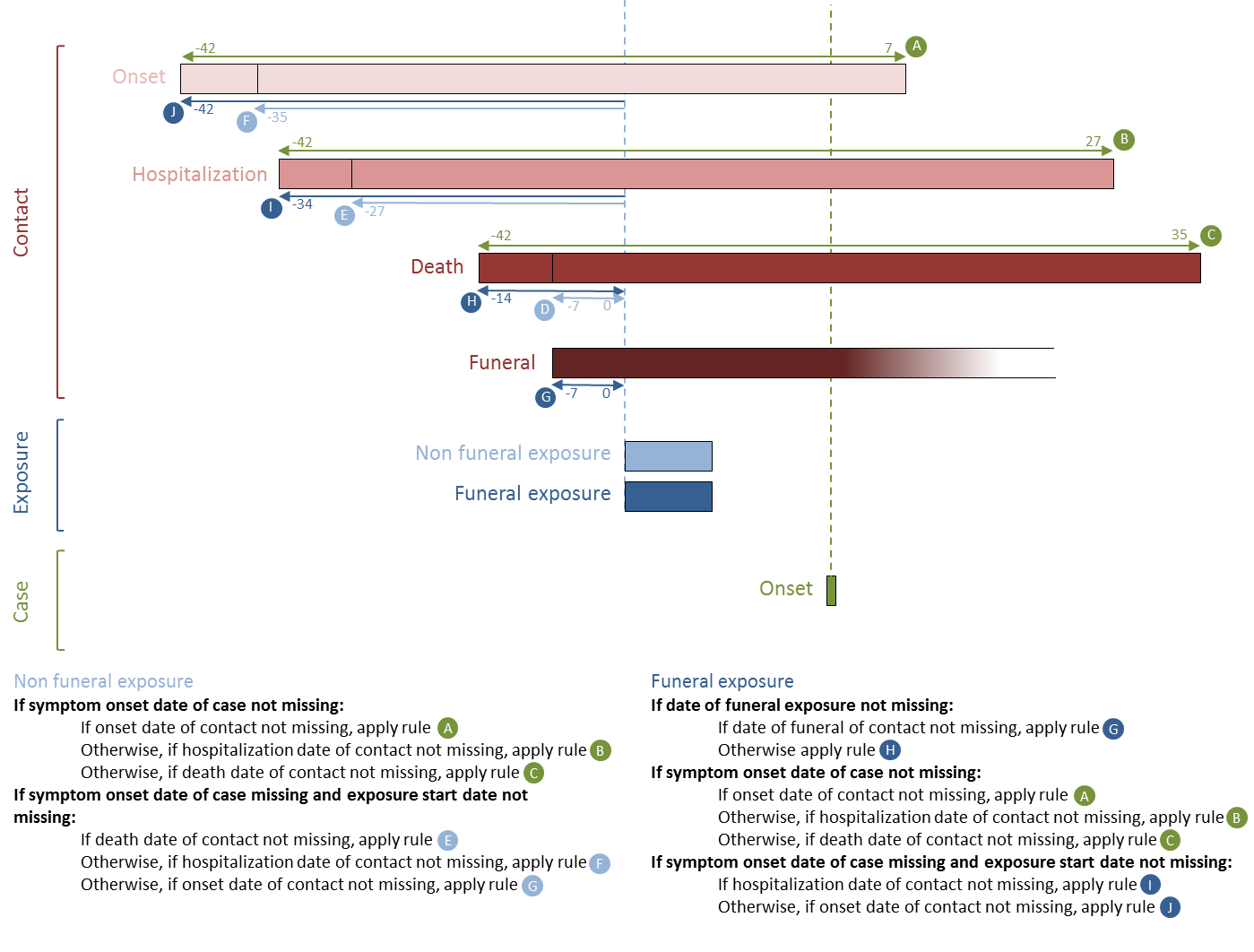


Figure a: Diagram explaining consistency checks comparing the clinical timelines of the contact to the symptom onset and dates of exposure of the case. All delays are in days.

### Hospital cleaning

Cases included in the line list were assigned a treatment centre type including (the list below) from information contained in several variables in the VHF country database.  The hospital name recorded in the VHF line lists was cross referenced between the WHO list of ETUs, holding centers and community care centers, and lists of hospitals, health centers, clinic etc available in the public domain (e.g., Ministry of Health websites or other UN sources).

### Country-specific data issues

Although we have accounted for biases and noise when analyzing the data (also see methods below), these issues vary between countries. We found that the data from Sierra Leone were more difficult to analyze. For example, it appeared that many groups of individuals had exactly the same first and last name, making identifying contacts in the line-list difficult. In addition, records from Sierra Leone, particularly in the early phase of the epidemic, seemed to generally be less complete and with more apparent errors, for instance in dates.

The data from Liberia were more complete, however the country database was several weeks behind the others.

Data on exposures in Guinea were very incomplete. Low numbers prevented us from analyzing these data in a meaningful way. More complete data, especially on exposures, may help better understand the epidemic in this country and interrupt transmission.

## Comparing the decreases in the proportions of HCWs and hospitalized cases over time

We assessed whether the decrease in the proportion of HCW among cases over time was due to decreased exposure to hospitalized patients. We used logistic regressions to model

1. The proportion of HCWs among cases over time (by month of onset),
2. The proportion of hospitalized patients over time (by month of onset).

We compared the estimated slopes of the two regressions to assess whether the decrease in the proportion of HCWs was faster than that in the proportion of hospitalized cases, which would indicate improvements in the protection of HCWs within the health care facilities over time.

To further understand the dependency between these two trends, we used a third logistic regression to model the proportion of HCWs as a function of both the proportion of hospitalized cases and time (by month of onset). This allowed us to assess whether the decrease in the proportion of HCWs over time was still significant, once adjusting for the proportion of hospitalized patients.

The analyses were performed on the time period from June 2014 to April 2015 and then repeated for July 2014 to April 2015.

## Estimation of the reproduction number by district

We estimated the monthly instantaneous reproduction number $R$ for each district, using the method described in [[3](#_ENREF_3)], and the district-level supplemented incidence data (as described in *[*[*1*](#_ENREF_1)*]*). In short, the estimates are obtained by averaging the daily reproduction number over a month. The daily estimates are based on the renewal equation $I\left( t \right)\sim Pois(R\left( t \right)\sum_{s=1}^{t} w\left( s \right)I\left( t-s \right))$ where $I(t)$ is the incidence of cases with symptoms onset on day $t$ in that district and $w$ is the distribution of the serial interval. $R(t)$ was only estimated when there had been at least 1 incident case in that district in the last 28 days preceding day $t$. In this method, the epidemics in each district are considered independent. The distribution of the serial interval was taken from early analysis of the epidemic [[3](#_ENREF_3)].

## Correlates for transmission intensity

Our aim was to explore the relationship between district level transmission intensity and other variables: proportion reporting funeral attendance amongst those reporting any exposure, proportion ever hospitalized and the proportion hospitalized within 4 days (4 days was the median delay from symptom onset to hospitalization, sensitivity analysis is in Figure m). We denote $p$ to be that district level variable. The transmission intensity was quantified by the reproduction number, $R$, which estimates the average number of secondary cases per index case *[*[*4*](#_ENREF_4)*]*. We estimate the reproduction number ($R_{m}^{d}$) and the proportion ($p_{m}^{d}$), for every district (d) in all three countries over monthly intervals (m).

### Estimating the true correlation between $\tilde{\mathbf{R}}$ and $\tilde{\mathbf{p}}$

We assume that the two variables are estimated with uncertainty, which we represent as follows:

$$(1)\left\{ \begin{aligned} R_{m}^{d}= \tilde{R_{m}^{d}}+\epsilon_{m}^{d} \\ p_{m}^{d}= \tilde{p_{m}^{d}}+ \delta_{m}^{d} \end{aligned} \right.$$

where $\tilde{R_{m}^{d}}$and $\tilde{p_{m}^{d}}$ are the true values with errors $\epsilon_{m}^{d}$ and $\delta_{m}^{d}$, respectively.

We compute the correlation coefficient between the true values $\tilde{R_{m}^{d}}$ and $\tilde{p_{m}^{d}}$ as:

$\left( 2 \right)Cor\left( \tilde{R_{m}^{d}}, \tilde{p_{m}^{d}} \right)=\frac{Cov (\tilde{R_{m}^{d}},\tilde{p_{m}^{d}})}{\sqrt{Var\left( \tilde{R_{m}^{d}} \right)*Var(\tilde{p_{m}^{d}})}}$

This correlation coefficient is different to that between the observed $R_{m}^{d}$and $p_{m}^{d}$, and explicitly accounts for the errors $\epsilon_{m}^{d}$ and $\delta_{m}^{d}.$

The numerator in the right-hand side of equation (2) simplifies to:

$$Cov (\tilde{R_{m}^{d}},\tilde{p_{m}^{d}})=Cov (R_{m}^{d}- \epsilon_{m}^{d}, p_{m}^{d}-\delta_{m}^{d})=Cov (R_{m}^{d}, p_{m}^{d})$$

assuming the errors $\epsilon_{m}^{d}$ and $\delta_{m}^{d}$ are independent of each other, $\epsilon_{m}^{d}$ is independent of $p_{m}^{d}$ and $\delta_{m}^{d}$ is independent of $R_{m}^{d}$.

We then compute the variances as:

$$Var\left( \tilde{R_{m}^{d}} \right)=Var\left( R_{m}^{d} \right)-Var(\epsilon_{m}^{d})$$

$$Var\left( \tilde{p_{m}^{d}} \right)=Var\left( p_{m}^{d} \right)-Var(\delta_{m}^{d})$$

assuming that the errors $\epsilon_{m}^{d}$ and $\delta_{m}^{d}$ are each independent of the true values $\tilde{R_{m}^{d}}$ and $\tilde{p_{m}^{d}}$.

${Var(\epsilon}_{m}^{d})$ is the median of the variances of the posterior distributions of $R_{m}^{d}$, computed as in *[*[*5*](#_ENREF_5)*].*

${Var(\delta}_{m}^{d})$ is the median of the variances of binomial proportions estimated from their exact binomial confidence intervals.

${Var(R}_{m}^{d})$ is the variance of the observed $R_{m}^{d}$ across month and district (the estimates are also derived as in [[3](#_ENREF_3)])*.*

${Var(p}_{m}^{d})$ is the variance of the observed proportions across month and district.

We computed p-values for this correlation coefficient using a two-sided permutation test, i.e. by randomly reordering the $R_{m}^{d}$ 50,000 times, computing the corresponding correlation coefficients and comparing the observed correlation coefficient to the distribution of permuted ones.

### Regression of $\tilde{\boldsymbol{R}}$ as a function of $\tilde{\boldsymbol{p}}$

We hypothesised that the correlation above resulted from hospitalization or funeral attendance directly affecting transmission intensity. In order to explore the nature of these trends, we explored linear regressions between the true reproduction number ($\tilde{R_{m}^{d}}$ ) and the true proportions ($\tilde{p_{m}^{d}}$), with the following underlying model:

$\tilde{R_{m}^{d}}=\alpha+\beta\tilde{p_{m}^{d}}$.

We used the method described by Ripley and Thompson *[*[*6*](#_ENREF_6)*]* which explicitly accounts for measurement errors as in Equation (1) to estimate the trend line, as implemented in the *deming* package in R *[*[*7*](#_ENREF_7)*]*. In brief, the method assumes that the observational errors $\epsilon_{m}^{d}$ and $\delta_{m}^{d}$ are independent and normally distributed. Hence the likelihood ($L$) is written as:

$$L=\prod_{m,d} L_{m}^{d}=\prod_{m,d} \frac{1}{Var\left( \epsilon_{m}^{d} \right)\sqrt{2\pi}}e^{-\frac{\left( \epsilon_{m}^{d} \right)^{2}}{Var\left( \epsilon_{m}^{d} \right)}}\frac{1}{Var\left( \delta_{m}^{d} \right)\sqrt{2\pi}}e^{-\frac{\left( \delta_{m}^{d} \right)^{2}}{Var\left( \delta_{m}^{d} \right)}}$$

$$=\prod_{m,d} \frac{1}{Var\left( \epsilon_{m}^{d} \right)\sqrt{2\pi}}e^{-\frac{\left( R_{m}^{d}- \tilde{R_{m}^{d}} \right)^{2}}{Var\left( \epsilon_{m}^{d} \right)}}\frac{1}{Var\left( \delta_{m}^{d} \right)\sqrt{2\pi}}e^{-\frac{\left( p_{m}^{d}- \tilde{p_{m}^{d}} \right)^{2}}{Var\left( \delta_{m}^{d} \right)}}$$

$$=\prod_{m,d} \frac{1}{Var\left( \epsilon_{m}^{d} \right)\sqrt{2\pi}}e^{-\frac{\left( R_{m}^{d}-(\alpha+\beta\tilde{p_{m}^{d}}) \right)^{2}}{Var\left( \epsilon_{m}^{d} \right)}}\frac{1}{Var\left( \delta_{m}^{d} \right)\sqrt{2\pi}}e^{-\frac{\left( p_{m}^{d}- \tilde{p_{m}^{d}} \right)^{2}}{Var\left( \delta_{m}^{d} \right)}}$$

L is maximised with respect to $\alpha$, $\beta$, and the $\tilde{p_{m}^{d}}$ using a numerical approach.

Confidence intervals for the regression line were estimated using the jackknife estimate of the variance-covariance matrix between the estimated intercept and slope of the regression.

The analysis was carried out by country (each point was a district-month) and overall.

The areas of the symbols on the plots are proportional to the point weights, calculated as $W_{m}^{d}= \frac{1}{Var(\epsilon_{m}^{d})+\beta^{2}Var(\delta_{m}^{d})}$ where $\beta$ is the slope of the regression line *[*[*6*](#_ENREF_6)*]*. These weights correspond to reciprocal of the variance of the error term in the linear regression, when seen as a regression between the observed $R_{m}^{d}$and $p_{m}^{d}$:

$\tilde{R_{m}^{d}}=\alpha+\beta\tilde{p_{m}^{d}}\underset{\Leftrightarrow}{}R_{m}^{d}-\epsilon_{m}^{d}=\alpha+\beta\left( p_{m}^{d}-\delta_{m}^{d} \right)\underset{\Leftrightarrow}{}R_{m}^{d}=\alpha+\beta p_{m}^{d}+(\epsilon_{m}^{d}-{\beta\delta}_{m}^{d})$

The main results we present in the main text and below restrict the analysis to district months where the denominator of $p_{m}^{d}$ is >4.

We performed a set of sensitivity analyses to assess the robustness of our findings (see below and section 2.5)

### Sensitivity analyses

We performed a set of sensitivity analyses to assess the robustness of our findings. Results of these sensitivity analyses are presented in section 2.5.

*Analysis based on district-months* where the denominator of $p_{m}^{d}$ *is >0, >4 (main analysis) or >9, and where np>5 & n(1-p)>5:*

First, our regression analysis relies on the assumption that the errors, in particular $\delta_{m}^{d}$, are normally distributed. Yet $\delta_{m}^{d}$ is the difference between an observed and a true proportion, namely $p_{m}^{d}$ and $\tilde{p_{m}^{d}}$. Therefore the normality assumption may only hold when the denominator of the observed proportion, $p_{m}^{d}$, is large. To assess the impact of this assumption on the estimated trend line, we therefore repeated the analyses omitting all the district-months where the denominator of $p_{m}^{d}$ was

- < 1,
- < 5 (main analysis),
- < 10
- np > 5 & n(1-p )> 5 (standard case in which binomial can be approximated by a normal distribution).

*Naïve analyses:*

Finally, we computed the “naïve” correlation coefficient and linear regression between the observed $R_{m}^{d}$ and $p_{m}^{d}$, not accounting for the errors $\epsilon_{m}^{d}$ and $\delta_{m}^{d}$. This allowed to us to investigate whether our findings were driven by explicitly accounting for these errors $\epsilon_{m}^{d}$ and $\delta_{m}^{d}$. The naïve analysis was only performed on district months where the denominator of $p_{m}^{d}$ *is >4.*

*Joint analyses:*

The method presented above does not easily generalize to more than one regressor. Therefore we could not analyze the joint influence of various regressors on local transmissibility whilst accounting for uncertainty.

To assess whether a joint model would be preferable, we considered the three following simple linear models, which do not account for uncertainty:

$$R_{m}^{d}=\alpha_{p}+\beta_{p}p_{m}^{d}+\epsilon_{p}$$

$$R_{m}^{d}=\alpha_{q}+\beta_{q}q_{m}^{d}+\epsilon_{q}$$

$$R_{m}^{d}=\alpha+\beta_{1}p_{m}^{d}+\beta_{2}q_{m}^{d}+\beta_{3}p_{m}^{d}q_{m}^{d}+\epsilon$$

where $p_{m}^{d}$ is the observed district level monthly proportion of cases reporting funeral exposure (among those reporting any exposure), $q_{m}^{d}$ is the observed district level monthly proportion of hospitalized cases who are hospitalized within 4 days of symptoms onset.

To exclude district months with very large uncertainty in $R_{m}^{d}, p_{m}^{d}$ or $q_{m}^{d}$, we restricted this analyses to district months with the denominator of $p_{m}^{d}$ and $q_{m}^{d}$>4..

We compared the adjusted r^2^ of the third model to the sum of the adjusted r^2^ of the first two. A higher r^2^ for the third model would indicate that a joint model is preferable.

*Sensitivity to measurement error assumption*

Our main analysis assumes that the errors $\epsilon_{m}^{d}$ and $\delta_{m}^{d}$ are each independent of the true values $\tilde{R_{m}^{d}}$ and $\tilde{p_{m}^{d}}$, which leads to:

$Var\left( \tilde{R_{m}^{d}} \right)=Var\left( R_{m}^{d} \right)-Var(\epsilon_{m}^{d})$

$Var\left( \tilde{p_{m}^{d}} \right)=Var\left( p_{m}^{d} \right)-Var(\delta_{m}^{d})$

As a sensitivity analysis we explored the alternative assumption that the errors $\epsilon_{m}^{d}$ and $\delta_{m}^{d}$ are each independent of the observed values $R_{m}^{d}$ and $p_{m}^{d}$, so that:

$Var\left( \tilde{R_{m}^{d}} \right)=Var\left( R_{m}^{d} \right)+Var(\epsilon_{m}^{d})$

$Var\left( \tilde{p_{m}^{d}} \right)=Var\left( p_{m}^{d} \right)+Var(\delta_{m}^{d})$

Results are presented in the last column of Table d.

## Fitting distributions to intervals from clinical event to exposure

Understanding when onwards exposure occurred in the clinical course of infection in a primary case could inform the focus of interventions, for instance on early detection of cases, or on improving safe funerals. We analyzed the timing of non-funeral exposure events relative to the following clinical events of the named contacts: onset of symptoms (referred to simply as onset here), hospitalization, and death. Here, we describe in detail the analysis of the time from onset to exposure; the analysis for hospitalization and death were similar.

We selected all matched non-funeral exposure events for which a start and/or end date of exposure was reported, and for which the contact had a reported onset date. Of these exposure events, we used those (13%) which had both start and end dates reported to compute an observed distribution of exposure durations. This distribution was then used to impute (by random draws) the missing dates of start or end of exposure. All exposure dates were rescaled to the number of days relative to the contact’s onset date, so that an exposure date on the day of onset became 0. Note that cases reporting exposures events with both start and end date were mainly from Liberia (78%), and were all reported before the end of 2014 (because the form changed afterwards to a single exposure date). Although the reported exposure windows were occasionally long (> 1 month), a large proportion of reported exposures (39%) were a single day exposure and a majority of the reported exposure windows (54%) were ≤ 3 days (see Figure n, panel A). There was no significant difference between the distribution of observed exposure durations in Liberia and Guinea (p=0.279), Liberia and Sierra-Leone (p=0.524) or Guinea and Sierra Leone (p=0.504). There was also no difference when comparing Liberia to Guinea and Sierra Leone combined (p=0.237).

We then fitted a distribution to the dates of exposure relative to onset allowing us to filter out the noise inherent in the data and focus on the signal present in the data. We fitted a mixture of two offset lognormal distributions: the more peaked we regarded as ‘the signal’, and the broader we regarded as ‘the noise’. Using single (non-mixture) distributions fitted the data much less well therefore the choice of a mixture of distributions was driven by data. Interpreting the mixture as a noise plus signal was a natural choice. There should be no reason why the error component would be particularly peaked, whereas variation in viral load, symptoms, behaviour, etc. over the course of illness would suggest that any peak observed is due to a real effect (the signal).

Each exposure event *k* contributed a factor *L_k_* in the likelihood, which quantified the probability that this exposure occurred between the corresponding start (*S_k_*) and end (*E_k_*) dates (relative to onset): $L_{k} = F\left( E_{k} \right) - F\left( S_{k} \right)$where F is defined by $F(x) = pF_{1}(x) + (1-p)F_{2}(x)$, with $F_{1}$ and $F_{2}$ the cumulative density functions of the two offset lognormal distributions, and p the mixture parameter. The contribution of a given exposure event *k* was weighted by 1/w_k_, where w_k_ was the overall number of exposures reported by the case. This accounted for the fact that only one of the reported exposures may have led to infection. Overall the likelihood was:

$$L = \prod_{k=1}^{N} L_{k}/w_{k}$$

Note that this formulation of the likelihood implicitly assumes independence between the contributions of each “pair” contact-case. However, 31% of pairs involved contacts who were named multiple (2 (17%) or more (14%)) times. When a contact is named multiple times, the timing of the corresponding onwards exposures might not be independent on each other. Accounting for this dependency is not straightforward. To assess the implications of our assumption, we ran a sensitivity analysis in which, when a contact belonged to several pairs, we randomly sampled one of these pairs. Otherwise, when a contact belonged only to one pair, we kept that pair. The results of that sensitivity analysis are extremely similar (for all three delays: onset to exposure, hospitalization to exposure, and death to exposure) and therefore not shown.

To quantify the variability in the timing of exposure events across pairs of individuals, a bootstrap procedure was applied. 3000 sets of N exposure events were sampled with replacement (N = total number of exposure events considered). For each set, the corresponding likelihood was calculated with respect to a new set of imputed dates, and maximized using the L-BFGS-B method of the ‘optim’ function in R *[*[*8*](#_ENREF_8)*]*.

The seven parameters we estimated were the offsets, means and variances of the two lognormal distributions as well as the mixture parameter *p*.

Characteristics of the fitted distribution (e.g. the mode or the probability density function) were derived from the bootstrap estimates of these seven parameters: here we report the median and 95% confidence intervals.

The analysis of timing of exposure events relative to hospitalization date of the contact was the same, albeit restricted to hospitalized matched contacts who had a reported hospitalization date.

The analysis of timing of exposure events relative to death date of the contact was similar but restricted to deceased matched contacts who had a reported death date. For this analysis, we used a mixture of two “reversed” lognormal distributions, i.e. $F(x) = pF_{1}(-x) + \left( 1-p \right)F_{2}(-x)$. This allowed us to model exposures that occurred potentially a long time before death but stopping shortly after death.

## Relative importance of onset and death in the timing of exposure events

The analyses described above showed that the non-funeral exposure events were concentrated shortly after onset of symptoms and around the day of death (see main text Fig 3). We performed an additional analysis to further explore whether one of these two clinical events was more influential for the timing of onwards non-funeral exposure events.

To address this question, we looked at whether the estimated Death-to-Exposure (DtoE) delay (see previous section), combined with the observed Onset-to-Death (OtoD) delay, was a good proxy of the estimated Onset-to-Exposure (OtoE) delay. A good match between these two distributions would indicate that the estimated Death to Exposure delay distribution is sufficiently informative to also be a good predictor of the Onset to Exposure delay distribution.

Similarly we compared the estimated Onset-to-Exposure delay, combined with the observed Onset-to-Death delay, to the estimated Death-to-Exposure delay. A good match between these two distributions would indicate that the estimated Onset-to-Exposure delay distribution is sufficiently informative to also be a good predictor of the Death-to-Exposure delay distribution.

We used numerical samples of size 5000 for all distributions. We constructed a sample OtoD from the observed Onset-to-Death delay distribution, as well as two samples OtoE and DtoE from the fitted mixture of offset lognormal distributions described in the previous section. We then compared (1) (OtoD + DtoE) and (OtoE) and (2) (OtoD – OtoE) to (DtoE). The ability of the fitted Death-to-Exposure delay distribution to reproduce the Onset-to-Exposure delay distribution was measured by computing the Kullback-Leibler divergence of (OtoD + DtoE) from (OtoE) *[*[*9*](#_ENREF_9)*]*. Similarly, the ability of the fitted Onset-to-Exposure delay distribution to reproduce the Death-to-Exposure delay distribution was measured by computing the Kullback-Leibler divergence of (OtoD – OtoE) from (DtoE).

The Kullback-Leibler divergence of a distribution P from a distribution Q measures the dissimilarity between the two distributions, with lower values indicating less disparity between the distributions.

## Analysis of the network of reported exposures

In order to characterize the heterogeneities in transmission, we analyzed the properties of the network formed by the links between the cases and the matched potential source contacts they reported. We looked at overall network made by all exposures together (counting only once multiple exposures between individuals) and separate networks for funeral and non-funeral exposures. Each of these networks is a directed network where nodes are Ebola patients (either cases or matched potential source contacts). Edges represent exposures from matched potential source contacts towards cases who reported them.

Separation of networks into funeral and non-funeral networks is not unambiguous, since cases sometimes name the same potential source contact as a funeral and non-funeral exposure. In those instances we counted exposures involving both funeral and non-funeral exposures to the funeral network, implicitly assuming, the funeral exposure was more likely to have led to infection. However, results were largely unchanged when we varied this assumptions (not shown), a result that can be explained by the fact that the different sub-networks represented by different types of exposure are very similar to each other.

For each of the three networks (overall, funeral and non-funeral), we analyzed the out-degree distribution, i.e. the distribution of the number of cases naming the same matched potential source contact, which can be seen as a proxy for the distribution of the number of secondary cases per index case. Several parametric distributions were fitted to each of the three observed out-degree distributions using maximum likelihood (ML) estimation (see later for a list of the distributions explored). The Akaike Information Criterion corrected for finite sample sizes (AICc) *[*[*10*](#_ENREF_10)*]* was then used to select the best distribution (with lowest AICc). For the best fitting distribution, 95% confidence intervals $[\theta_{inf};\theta_{sup}]$ on the parameter(s) $\theta$ were obtained using the likelihood ratio test. Confidence intervals on the corresponding distribution, $p^{'\left( k \right)}$, were then obtained numerically by sampling 5000 times from the Normal distribution with mean the ML estimate $\hat{\theta}$ and standard deviation ${(\theta}_{sup}- \theta_{inf})/(2*1.96)$, and computing the corresponding 95% quantiles of the corresponding distributions $p^{'\left( k \right)}$.

We derived the variance and the coefficient of variation for the offspring distribution, based on the assumption that the network shown in Figure p is a sample of the full transmission network, see below.

### Parameterizations used for the distributions

Let *a* be the generic first parameter and *b* be the second parameter, *k* be the degree and *p* the standard probability mass function defined over non-negative integers. Because the distributions are conditional on being named at least once as a contact, we use the re-scaled marginal distributions $p^{'\left( k \right)}=\frac{p\left( k \right)}{1-p\left( 0 \right)}$.

**Poisson distribution (1 parameter a)**

$$p'\left( k \right)=\frac{a^{k}e^{-a}}{k!\left( 1-e^{-a} \right)}$$

**Geometric distribution (1 parameter a)**

$$p'\left( k \right)={a\left( 1-a \right)}^{k-1}$$

**Negative binomial distribution (2 parameters a, b)**

$$p'\left( k \right)=\frac{\Gamma\left( b+k \right)}{\Gamma\left( b+1 \right)\Gamma\left( k \right)}\frac{\left( 1-a \right)^{b}a^{k}}{1-\left( 1-a \right)^{b}}$$

**Logarithmic distribution (1 parameter a)**

$$p^{'}\left( k \right)=-\frac{1}{\ln\left( 1-a \right)}\frac{a^{k}}{k}$$

**Zipf distribution (1 parameter a)**

$$p^{'}\left( k \right)=\frac{1/k^{a}}{\sum_{m=0}^{K} 1/m^{a}}$$

All distributions are defined with support on the positive integers, except the Zipf distribution which has support 1,…,K, with K chosen as the largest observed value of *k*.

### Coefficient of Variation of the out-degree distribution

The aim of this section is to derive the variance and the coefficient of variation for the offspring distribution, based on the assumption that the network shown in Figure p is a random sample of the full transmission network. In [12], Stumpf et al, explore the relationship between true networks and random subsamples. They highlight that the degree distribution of a samples subnet may be different and even have a different functional form to the original underlying network. For this reason, we instead estimate only the variance of the underlying network, which we can do for known mean (the mean is the reproduction number). We make varying assumptions about the mean (either 1 or 1.5) to obtain a range of estimates.

Let $p\left( \nu\right)$ be the offspring distribution, i.e. the probability that an Ebola case infected $\nu$ other people, where $\nu$ can be any non-negative integer. The mean of the offspring distribution $p\left( \nu\right)$ is the reproduction number *R*. Denote $\sigma^{2}$ its variance.

Let $\varphi\left( m \right)$ be the probability that an individual is named *m* times as a contact by cases. If we assume that our data are a random sample of a proportion *s* of all cases, then

$$\varphi\left( m \right)=\sum_{\nu\geq m}^{\infty} Bin\left( m|s,\nu\right) p\left( \nu\right)$$

where *Bin* denotes the standard binomial distribution.

The distribution that we observed is not $\varphi\left( m \right)$ but rather the distribution $\varphi^{+}\left( m \right)=\varphi\left( m \right)/\left( 1-\varphi(0) \right)$ which is conditional on a case being named at least once as a contact, so with *m* strictly positive and

$$\varphi(0)=\sum_{\nu\geq0}^{\infty} \left( 1-s \right)^{\nu} p\left( \nu\right)$$

is the probability that a case is not named as a contact by anyone.

Denote the mean of the distribution $\varphi^{+}\left( m \right)$ as *T* and its variance as *W*. From the network shown in Figure p, we find that *T*=1.56 and *W*=1.35.

Using standard properties of the binomial distribution, we find after some algebraic manipulation, that

$$T=\frac{sR}{1-\varphi\left( 0 \right)}$$

and

$$W=T\left( 1-s-\varphi\left( 0 \right)T \right)+\frac{s^{2}\sigma^{2}}{1-\varphi\left( 0 \right)}$$

We explore two sets of assumptions to estimate $\sigma^{2}$. First, we assume that the network represents an almost complete part of the epidemic, so that the mean reproduction number is close to one (which is the case for a completed epidemic), i.e. $R\approx1$. Second, and more conservative from the perspective of estimating $\sigma^{2}$, we assume that this is a sample from the growing epidemic, when $R\approx1.5$.

In terms of the sampling fraction *s*, our best estimate is that *s*=0.326*0.139=0.045 (32.6% of cases in the line-list report an exposure, and 13.9% of named contacts can be matched). We obtain a more conservative estimate (from the perspective of estimating $\sigma^{2}$), by assuming that if contacts can be matched for one reported exposure, then they can be matched for all reported exposures, in which case *s*=0.326.

We then estimate $\varphi\left( 0 \right)$, $\sigma^{2}$ and the coefficient of variation, $\sigma/R$.

For the first set of assumptions we obtain, that,$\varphi\left( 0 \right)=0.971$ that is that 97.1% of cases were named by no-one. In this case $\sigma^{2}$=31.5 and the coefficient of variation is 5.61. Note that as 753 named contacts were matched amongst 19,618 cases, the empirical estimate of $\varphi\left( 0 \right)$ is 96.2%, i.e. very similar to the expectation derived here. For the second set of assumptions we obtain, that,$\varphi\left( 0 \right)=0.686$ , $\sigma^{2}$=5.81 and the coefficient of variation is 1.61.

The negative binomial offspring distributions is often used to quantify super-spreading *[*[*11*](#_ENREF_11)*]*; the coefficient of dispersion *k* for the negative binomial distribution is related to the mean *R* and variance $\sigma^{2}$ by

$$k=\frac{R^{2}}{\sigma^{2}-R}$$

so we estimate that *k*=0.03 for the first set of assumptions and *k*=0.52 for the second. The lower the estimate of *k*, the more transmission is characterized by super-spreading. To place this in context, for SARS the central estimate was *k*=0.16 with 90% confidence intervals 0.11-0.64). These estimates for Ebola, though less well defined than for SARS due to the limited sampling of the transmission network and limited ability to match cases and contacts, imply that super-spreading has been a feature of the Ebola epidemic to date.

## List of possible predictors of naming an exposure, or being named and being named multiple times as a contact

The following predictors were included in this univariate analysis: case definition (Confirmed, Probable); sex; age (<16yrs, ≥16 yrs, based on common epidemiological definition of adulthood); whether individuals were hospitalized; the date of report (Dec13-May14, Jun14-Nov14, Dec14-May15, Missing, chosen to represent early, peak and declining phases of the epidemic, respectively); the delays that individuals experienced between onset to hospitalization (≥ 4 days or <4 days, chosen as 4 days was the median delay), onset to death (≥ 6 days or <6 days, chosen as 6 days was the median delay), onset to discharge (≥ 16 days or <16 days, chosen as 16 days was the median delay) and report to death (≥ 0 days or <0 days, chosen to distinguish cases reported before or after death); whether individuals were health care workers (HCWs); the country database they belonged to; the final clinical outcome for the case (dead, alive, unknown); whether the case reported to have travelled outside their village / town recently; whether they reported having attended a funeral (and touched a corpse) and the following recorded symptoms that the case had experienced at the time of completion of the Case Report Form: fever, diarrhea, vomiting (+- blood), signs of bleeding (unexplained, gums, at injection site, nose, stool, vaginal, skin, urine or other signs of bleeding), unconsciousness, confusion, respiratory illness (chest pain, cough (+- blood), difficulty breathing and hiccups), and other (fatigue, anorexia, abdominal pain, muscle pain, joint pain, headache, difficulty swallowing, jaundice, conjunctivitis, rash, pain in eyes and sore throat). Information was available on the occupation of 41% cases but the number of cases in each category was too small to assess whether an occupation type was a significant predictor, apart from HCW status. Missing values were treated as separate predictors, since missing data may be informative about how cases were detected.

# Additional results

## Number of reported exposures

Table a: Number of Confirmed and Probable (CP) cases naming 0, 1, 2, or 3 non-funeral exposures and 0, 1, or 2 funeral exposures.

|  | | **Number of funeral exposures reported** | | | | | | | | | | | |
| --- | --- | --- | --- | --- | --- | --- | --- | --- | --- | --- | --- | --- | --- |
|  |  | **All** | | | **Guinea** | | | **Liberia** | | | **Sierra Leone** | | |
|  |  | **0** | **1** | **2** | **0** | **1** | **2** | **0** | **1** | **2** | **0** | **1** | **2** |
| **Number of non-funeral exposures reported** | **0** | 13215 | 238 | 9 | 2637 | 39 | 1 | 3265 | 47 | 2 | 7313 | 152 | 6 |
|  | **1** | 3503 | 1592 | 49 | 451 | 231 | 0 | 1465 | 250 | 14 | 1587 | 1111 | 35 |
|  | **2** | 576 | 217 | 73 | 106 | 44 | 2 | 185 | 25 | 14 | 285 | 148 | 57 |
|  | **3** | 104 | 19 | 23 | 14 | 3 | 1 | 67 | 5 | 4 | 23 | 11 | 18 |


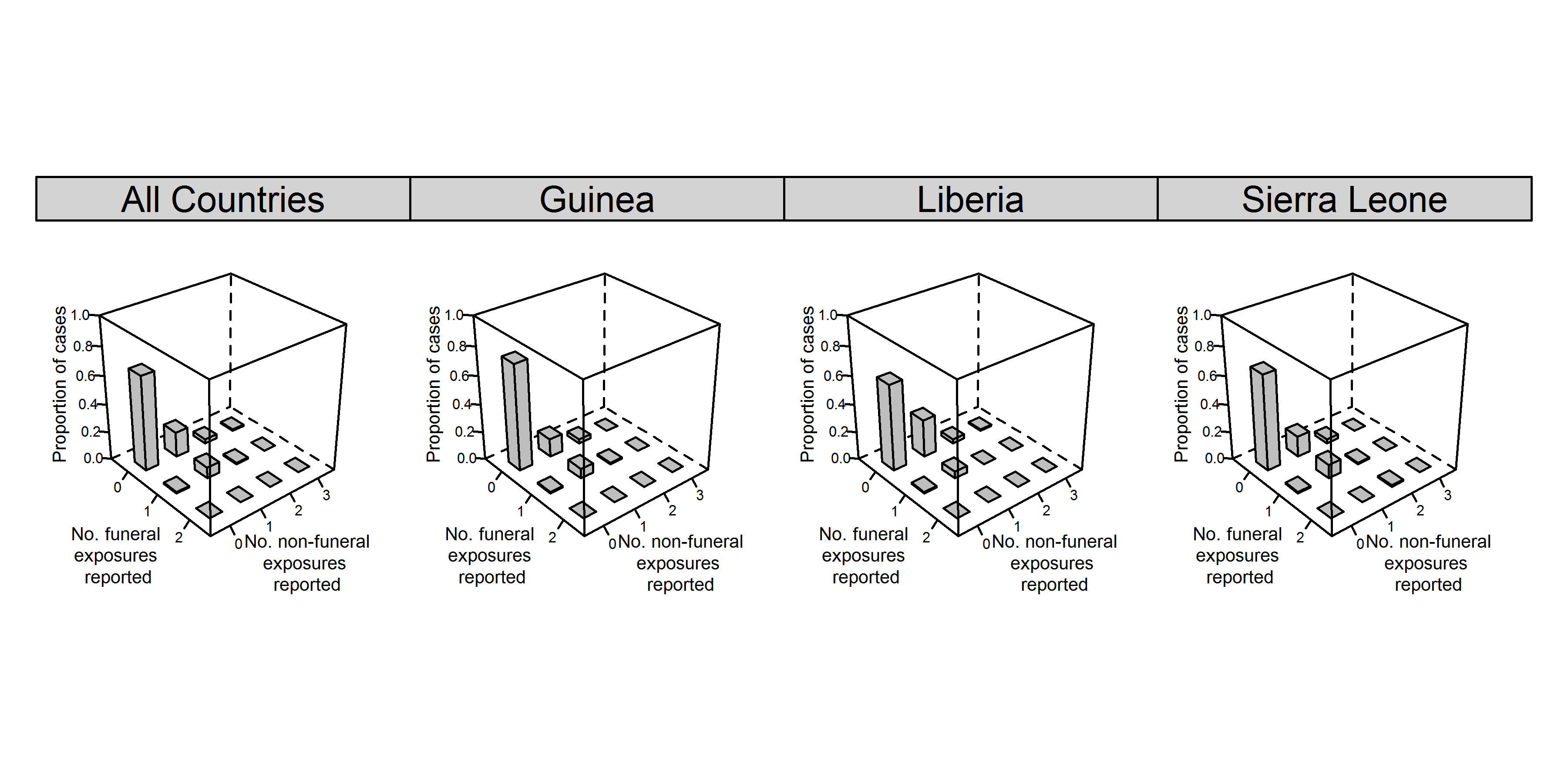


Figure b: Proportion of CP cases naming 0,1,2, or 3 non-funeral exposures and 0, 1, or 2 funeral exposures.

## Representativeness of the exposure data

In Liberia and Sierra Leone, cases who reported exposures were roughly a constant fraction of all cases within a district (18% of all cases in Liberia and 33% in SL) (Figure c, panel A). In Guinea the pattern is less clear with overall ~15% of cases reporting exposures but larger variability by district.

In all three countries our ability to match contacts didn’t vary substantially by district, but was overall higher in Guinea, with ~36% of cases reporting exposures for which we could match at least one exposure, vs 21% in Liberia and 20% in SL (Figure c, panel B).


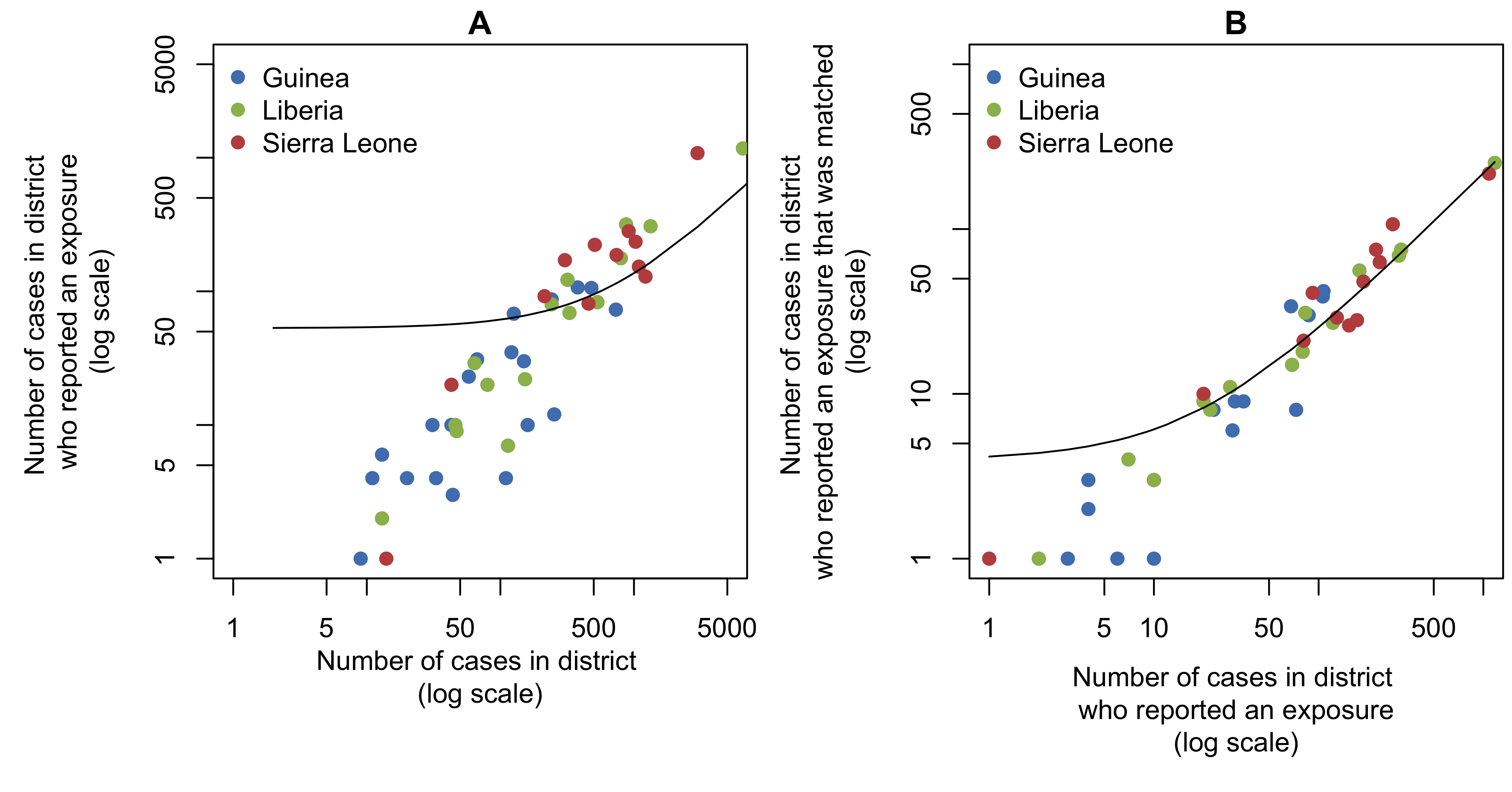


Figure c: Illustrating the representativeness of the exposure data. A: Correlation between cumulative number of cases by district and cumulative number of cases reporting an exposure. The slope of the regression is 0.08 and the adjusted r^2^ is 0.31, note that the plot is on a log-log scale but that the regression was done on the natural scale. By country, the slopes are 0.15, 0.18 and 0.33 for Guinea, Liberia and Sierra Leone respectively, with adjusted r^2^ 0.60, 0.97 and 0.83. B: Correlation between cumulative number of cases reporting an exposure and cumulative number of cases reporting a contact that was identified. The slope of the regression is 0.21 and the adjusted r^2^ is 0.96, again the plot is on a log-log scale but the regression was on the natural scale. By country the slopes are 0.36, 0.21 and 0.20 for Guinea, Liberia and Sierra Leone, with adjusted r^2^ of 0.88, 0.99 and 0.91.

### Predictors for reporting non-funeral exposures

Individuals more likely to report non-funeral exposures were: those who had symptoms, those who survived, females, those who were hospitalized within 4 days of onset, and those under 16 years of age (Table b). Additionally, cases who were reported in June to November 2014 or and December 2014 – May 2015 were less likely to report a non-funeral exposure than cases reported in December 2013 – May 2014. Cases who died before they were reported were less likely to report non-funeral exposures.

Table b: Multivariable logistic regression, comparing the odds of CP cases naming a non-funeral exposure compared to CP cases answering “no” to non-funeral exposures and those not asked the question about exposure. Ref denotes reference group. The most parsimonious yet adequate model (determined by the AIC, see methods section) is presented. Covariates that do not contribute to this model are not presented.

| **Category** | **Name** | **Odds Ratio** | **95% CI** | **p** |
| --- | --- | --- | --- | --- |
| Country | Guinea | Ref |  |  |
|  | Liberia | 1.39 | 1.21 - 1.61 | <0.001 |
|  | Sierra Leone | 1.01 | 0.89 - 1.15 | 0.880 |
| Case Definition | Confirmed | Ref |  |  |
|  | Probable | 2.03 | 1.85 - 2.23 | <0.001 |
| Sex | Male | Ref |  |  |
|  | Female | 1.20 | 1.12 - 1.29 | <0.001 |
|  | Missing | 1.08 | 0.61 - 1.86 | 0.782 |
| Fever | No | Ref |  |  |
|  | Yes | 1.79 | 1.59 - 2.01 | <0.001 |
|  | Missing | 0.96 | 0.79 - 1.16 | 0.672 |
| Diarrhea | No | Ref |  |  |
|  | Yes | 1.19 | 1.09 - 1.29 | <0.001 |
|  | Missing | 1.29 | 1.12 - 1.48 | <0.001 |
| Unconscious | No | Ref |  |  |
|  | Yes | 0.89 | 0.68 - 1.15 | 0.367 |
|  | Missing | 0.70 | 0.64 - 0.77 | <0.001 |
| Final Outcome | Alive | Ref |  |  |
|  | Dead | 0.74 | 0.64 - 0.87 | <0.001 |
|  | Missing | 0.74 | 0.66 - 0.82 | <0.001 |
| Hospitalized | No | Ref |  |  |
|  | Yes ETU | 0.78 | 0.67 - 0.92 | 0.002 |
|  | Yes not ETU | 0.68 | 0.58 - 0.80 | <0.001 |
|  | Yes, unknown hospital type | 1.04 | 0.93 - 1.17 | 0.482 |
|  | Missing | 0.52 | 0.45 - 0.61 | <0.001 |
| Travelled+ | No | Ref |  |  |
|  | Yes | 2.04 | 1.75 - 2.38 | <0.001 |
| Onset to hospitalisation (days) | ≥ 4 | Ref |  |  |
|  | < 4 | 1.29 | 1.15 - 1.43 | <0.001 |
|  | Missing | 1.32 | 1.15 - 1.52 | <0.001 |
| Onset to death (days) | ≥ 6 | Ref |  |  |
|  | < 6 | 0.81 | 0.71 - 0.93 | 0.002 |
|  | Missing | 0.72 | 0.57 - 0.91 | 0.006 |
| Number of days between report and death++ | ≥ 0 | Ref |  |  |
|  | < 0 | 0.70 | 0.59 - 0.83 | <0.001 |
|  | Missing | 1.60 | 1.27 - 2.01 | <0.001 |
| Bleeding symptoms | No | Ref |  |  |
|  | Yes | 0.86 | 0.73 - 1.00 | 0.051 |
|  | Missing | 0.79 | 0.70 - 0.88 | <0.001 |
| Respiratory symptoms | No | Ref |  |  |
|  | Yes | 1.05 | 0.96 - 1.14 | 0.267 |
|  | Missing | 0.77 | 0.66 - 0.89 | 0.001 |
| Other symptoms | No | Ref |  |  |
|  | Yes | 1.73 | 1.43 - 2.09 | <0.001 |
|  | Missing | 0.27 | 0.20 - 0.36 | <0.001 |
| Age group | < 16 yrs | Ref |  |  |
|  | ≥ 16 yrs | 0.68 | 0.62 - 0.74 | <0.001 |
|  | Missing | 0.43 | 0.31 - 0.59 | <0.001 |
| Date of Report | Dec13-May14 | 1.91 | 1.46 - 2.49 | <0.001 |
|  | Jun14-Nov14 | Ref |  |  |
|  | Dec14-May15 | 1.27 | 1.15 - 1.39 | <0.001 |
|  | Missing | 0.69 | 0.59 - 0.80 | <0.001 |

+ The case reported travelling outside their home or village/town before becoming ill

++ A negative number indicates death precedes reporting of the case

### Predictors for reporting funeral exposures

Individuals more likely to report funeral exposures were: from Guinea or Sierra Leone, Probable cases, females, those with symptoms, those who reported travelling to a different district recently, (Table c). Additionally, cases who were reported in December 2013 to May 2014 were more likely to report funeral exposures than those in June 2014 to November 2014.

Table c: Multivariable logistic regression, comparing the odds of CP cases naming a funeral exposure compared to CP cases answering “no” to funeral exposures and those not asked the question about exposure. The most parsimonious yet adequate model (determined by the AIC, see methods section) is presented. Covariates that do not contribute to this model are not presented.

| **Category** | **Name** | **Odds Ratio** | **95% CI** | **p** |
| --- | --- | --- | --- | --- |
| Country | Guinea | Ref |  |  |
|  | Liberia | 0.30 | 0.24 - 0.36 | <0.001 |
|  | Sierra Leone | 1.00 | 0.85 - 1.18 | 0.959 |
| Case Definition | Confirmed | Ref |  |  |
|  | Probable | 1.47 | 1.31 - 1.66 | <0.001 |
| Sex | Male | Ref |  |  |
|  | Female | 1.20 | 1.09 - 1.31 | <0.001 |
|  | Missing | 0.59 | 0.17 - 1.47 | 0.313 |
| HCW | No | Ref |  |  |
|  | Yes | 0.48 | 0.37 - 0.63 | <0.001 |
| Fever | No | Ref |  |  |
|  | Yes | 1.66 | 1.41 - 1.96 | <0.001 |
|  | Missing | 1.09 | 0.84 - 1.42 | 0.513 |
| Diarrhea | No | Ref |  |  |
|  | Yes | 1.27 | 1.14 - 1.43 | <0.001 |
|  | Missing | 1.31 | 1.08 - 1.58 | 0.005 |
| Unconscious | No | Ref |  |  |
|  | Yes | 1.16 | 0.85 - 1.57 | 0.349 |
|  | Missing | 0.60 | 0.53 - 0.68 | <0.001 |
| Hospitalized | No | Ref |  |  |
|  | Yes ETU | 0.57 | 0.50 - 0.66 | <0.001 |
|  | Yes not ETU | 0.66 | 0.57 - 0.76 | <0.001 |
|  | Yes, unknown hospital type | 0.83 | 0.72 - 0.96 | 0.011 |
|  | Missing | 0.53 | 0.43 - 0.65 | <0.001 |
| Travelled+ | No | Ref |  |  |
|  | Yes | 3.99 | 3.41 - 4.66 | <0.001 |
| Bleeding symptoms | No | Ref |  |  |
|  | Yes | 0.85 | 0.69 - 1.04 | 0.111 |
|  | Missing | 0.81 | 0.70 - 0.94 | 0.007 |
| Respiratory symptoms | No | Ref |  |  |
|  | Yes | 1.30 | 1.16 - 1.45 | <0.001 |
|  | Missing | 0.98 | 0.80 - 1.21 | 0.860 |
| Other symptoms | No | Ref |  |  |
|  | Yes | 1.64 | 1.23 - 2.21 | 0.001 |
|  | Missing | 0.33 | 0.21 - 0.50 | <0.001 |
| Age group | < 16 yrs | Ref |  |  |
|  | ≥ 16 yrs | 1.25 | 1.11 - 1.42 | <0.001 |
|  | Missing | 0.60 | 0.33 - 1.01 | 0.070 |
| Date of Report | Dec13-May14 | 1.44 | 1.02 - 2.02 | 0.036 |
|  | Jun14-Nov14 | Ref |  |  |
|  | Dec14-May15 | 0.91 | 0.80 - 1.03 | 0.136 |
|  | Missing | 0.85 | 0.69 - 1.03 | 0.100 |

+ The case reported travelling outside their home or village/town before becoming ill.

## Patterns in reported exposures

### Reported relationships between case and contact


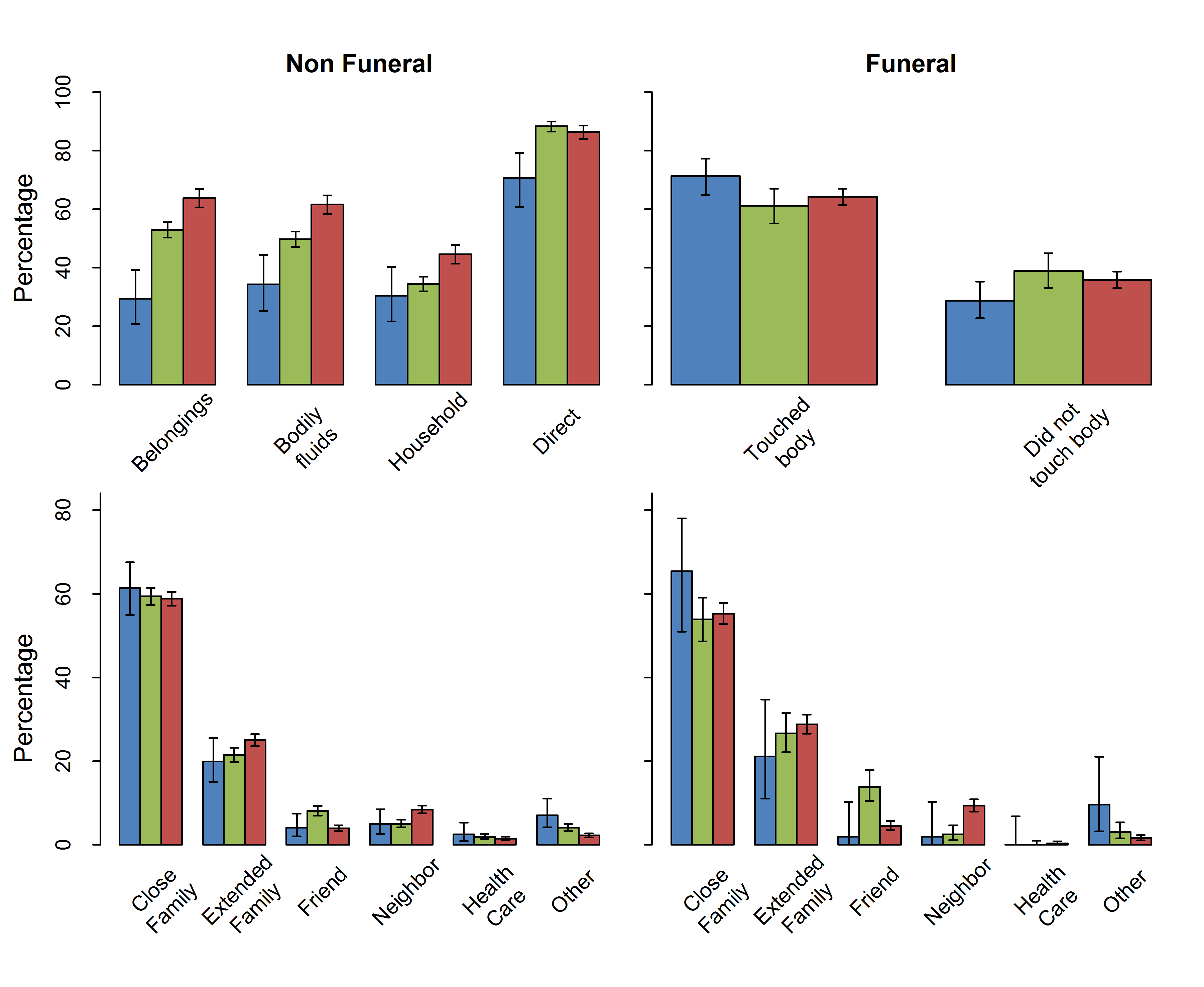


Figure d: Type of exposures (top) and relationship of named contacts (bottom) for non-funeral (left) and funeral (right) exposures. The graphs show the proportion of reported exposures which were reported as involving a particular type of contact. The bars are colored according to country: Guinea (blue), Liberia (green) and Sierra Leone (red). Type of non-funeral contacts are: touched/shared belongings; touched bodily fluids; spent time in the same household; direct physical contact. Type of non-funeral contact is not always reported. Relationship was not reported for every exposure, we grouped reported relationships into types: “Close Family” is defined as siblings, marital and parent-child relationships, other family members are considered “Extended Family”. “Neighbor” is defined as tenants, lodgers, landlords and neighbors; “Health Care” is defined as HCW-patient relationships and caregivers, or any reference to a patient; “Other” includes traditional healers, contacts through religious practice and transport contacts.

### Non-funeral exposures over time


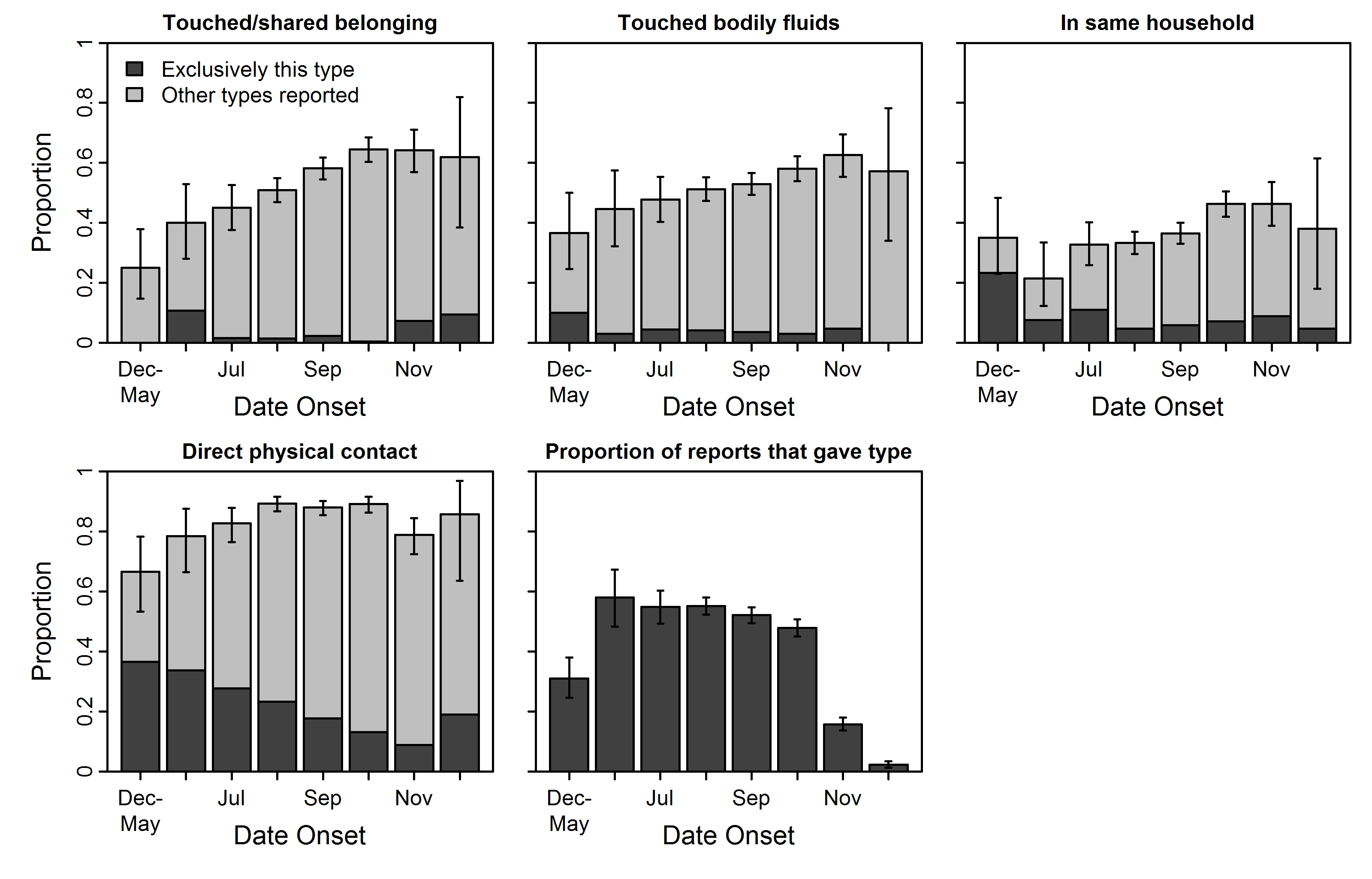


Figure e: Patterns in non-funeral exposure type over time, in all three countries. Up until the new form was introduced (late 2014, as seen in the final plot), individuals could report up to four different types of non-funeral exposure: Touched or shared the linens, clothes, or dishes/eating utensils of the case; Touched the body fluids of the case (blood, vomit, saliva, urine, faeces); Slept, ate, or spent time in the same household or room as the case; Had direct physical contact with the body of the case (alive or dead). Here we report the proportion of exposures reporting each type, either exclusively (dark grey) or with other types (light grey).


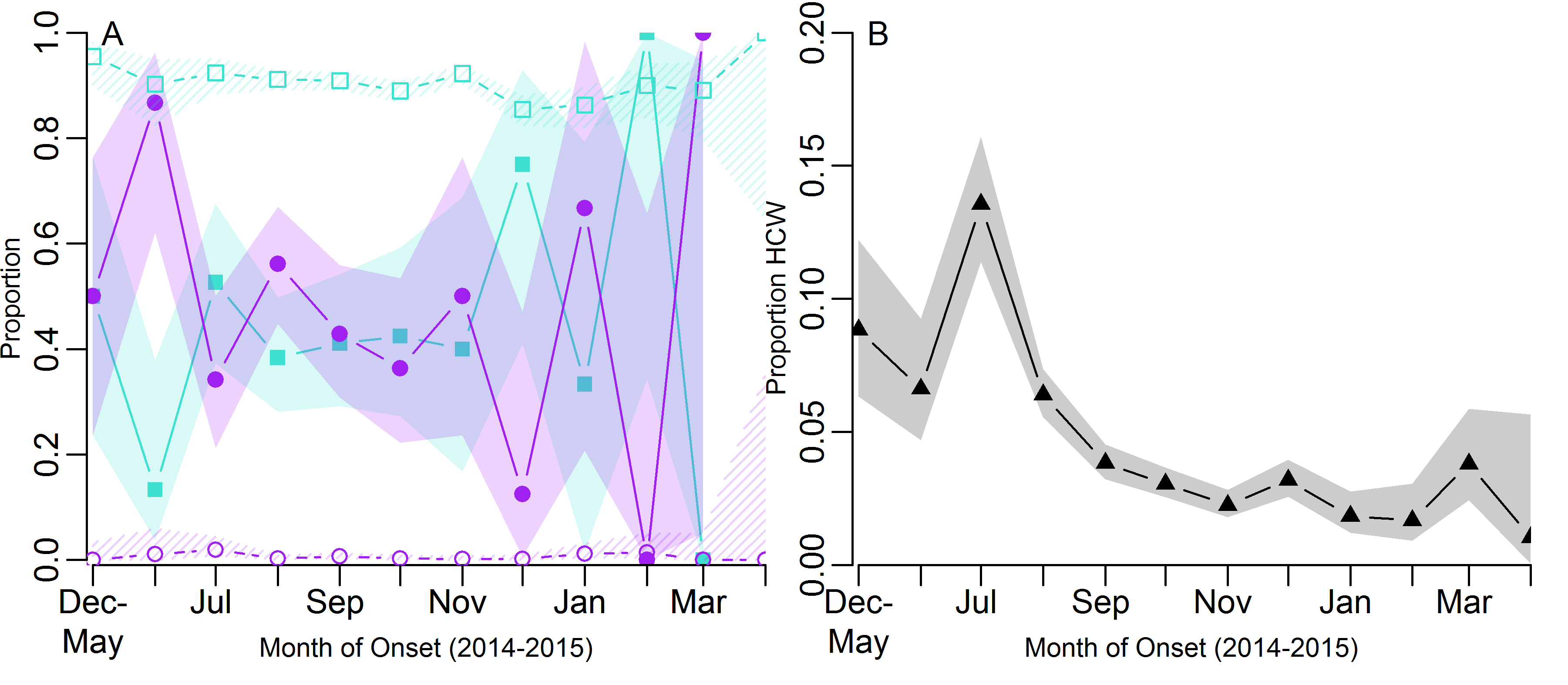


**Figure f: A: Proportion of non-funeral exposures reported by HCWs (solid shading) and non-HCWs (hashed shading) which are with close or extended family or friends (turquoise) and with patients and HCWs (purple), by month. (Note that for HCW we have assumed that exposures reported with colleagues are with a HCW.) B: Proportion of cases who are health care workers (HCWs) in the WHO line-list, by month of symptom onset.**


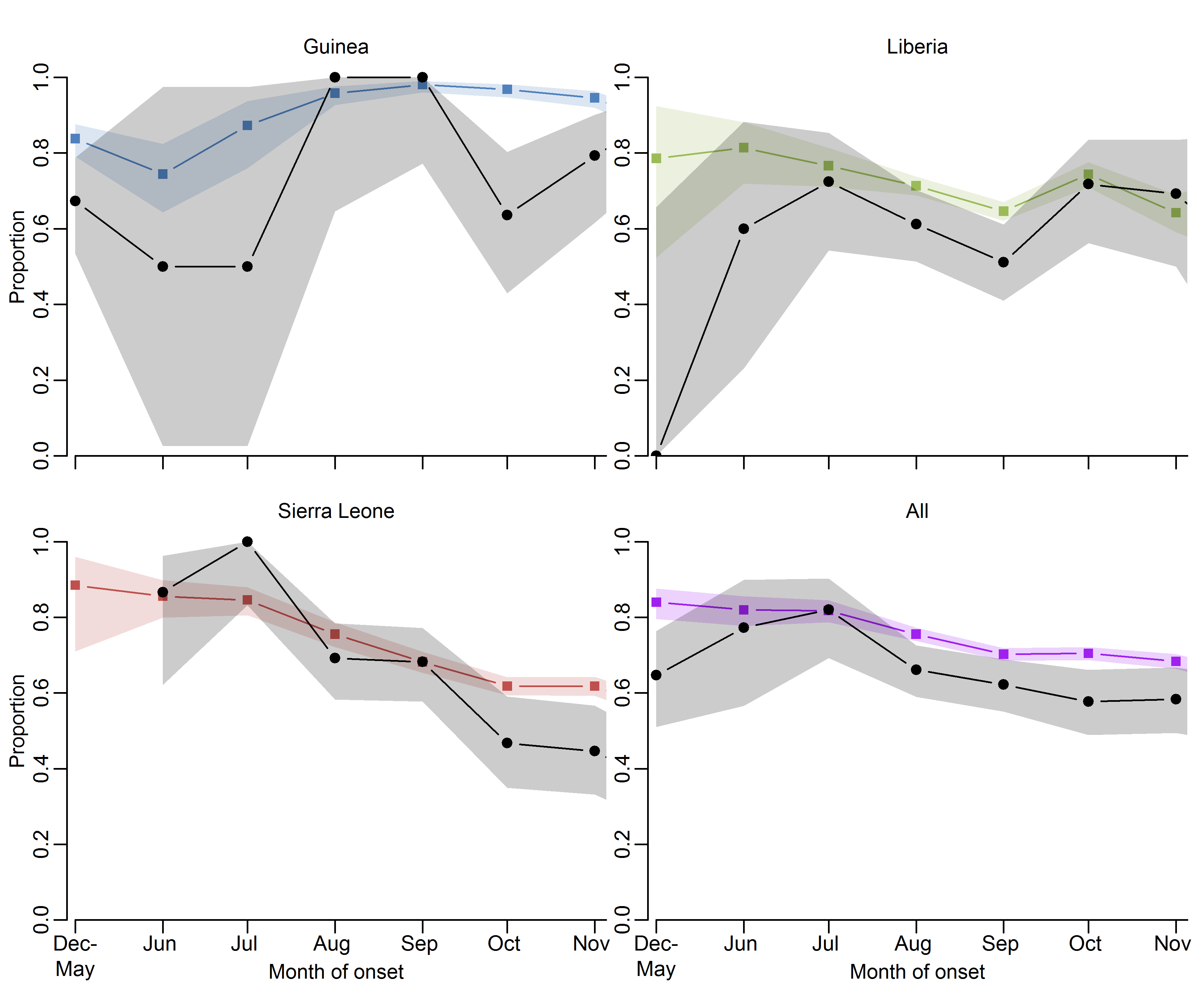


Figure g: Proportion of hospitalized cases, by month of onset, amongst all cases in the WHO line-list (colored lines) and among those named as a non-funeral contact (black lines).

### ****Funeral exposures**** over time


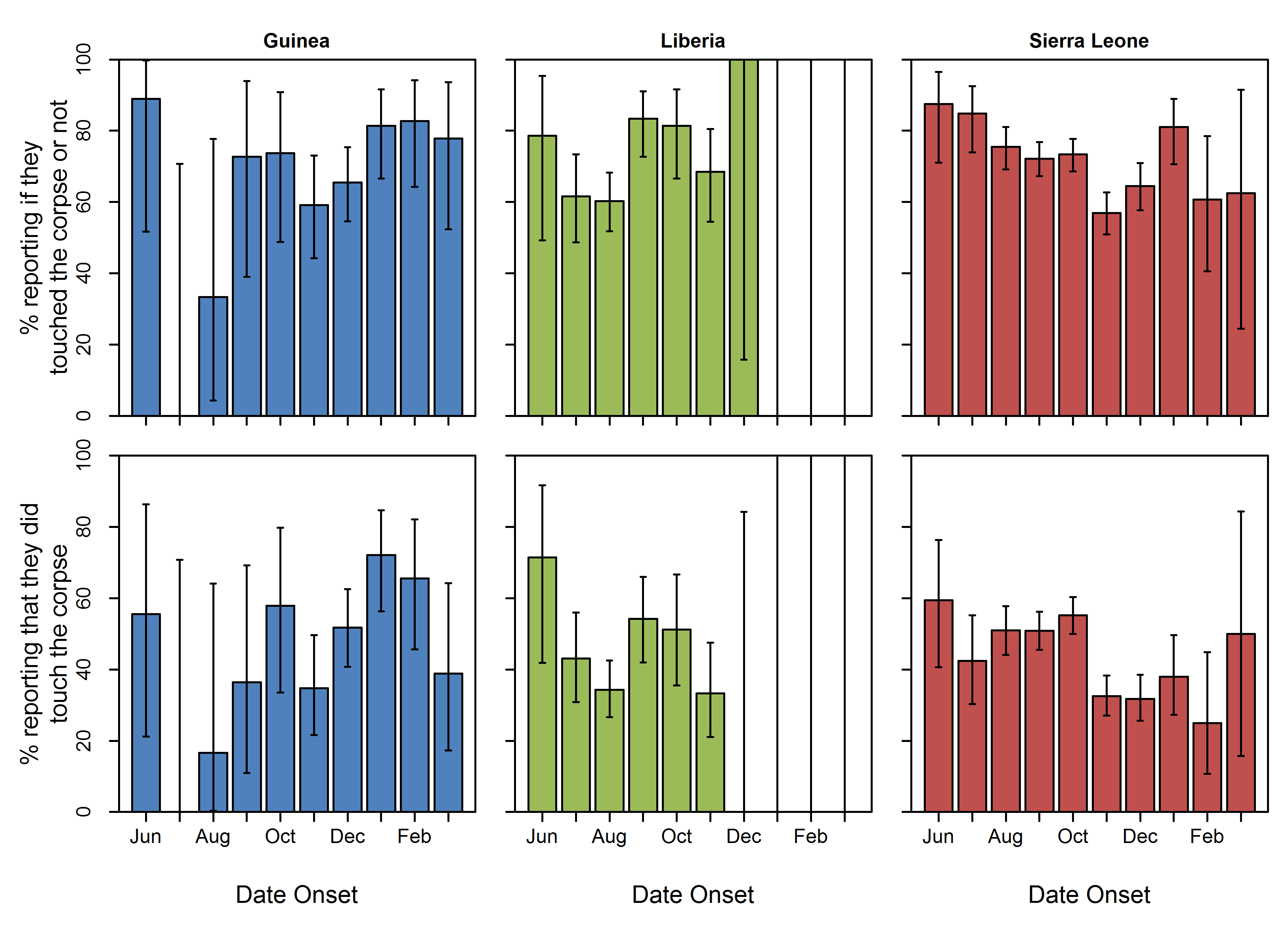


Figure h: Type of funeral exposure over time, by country. Percentage of cases reporting funeral exposure that answered the question on whether they touched or did not touch the corpse (top row). Percentage of cases reporting funeral exposure that answered the question on touching, who did report touching the corpse (bottom row). The proportion reporting they touched the corpse was higher overall between June to October 2014 than in November 2014 to March 2015 (50% vs 38%, p<0.001); this was driven by Sierra Leone (52% vs 33%, p<0.001). Trends were not significant in Liberia and Guinea.

### ****Exposures where the contact could be matched**** over time


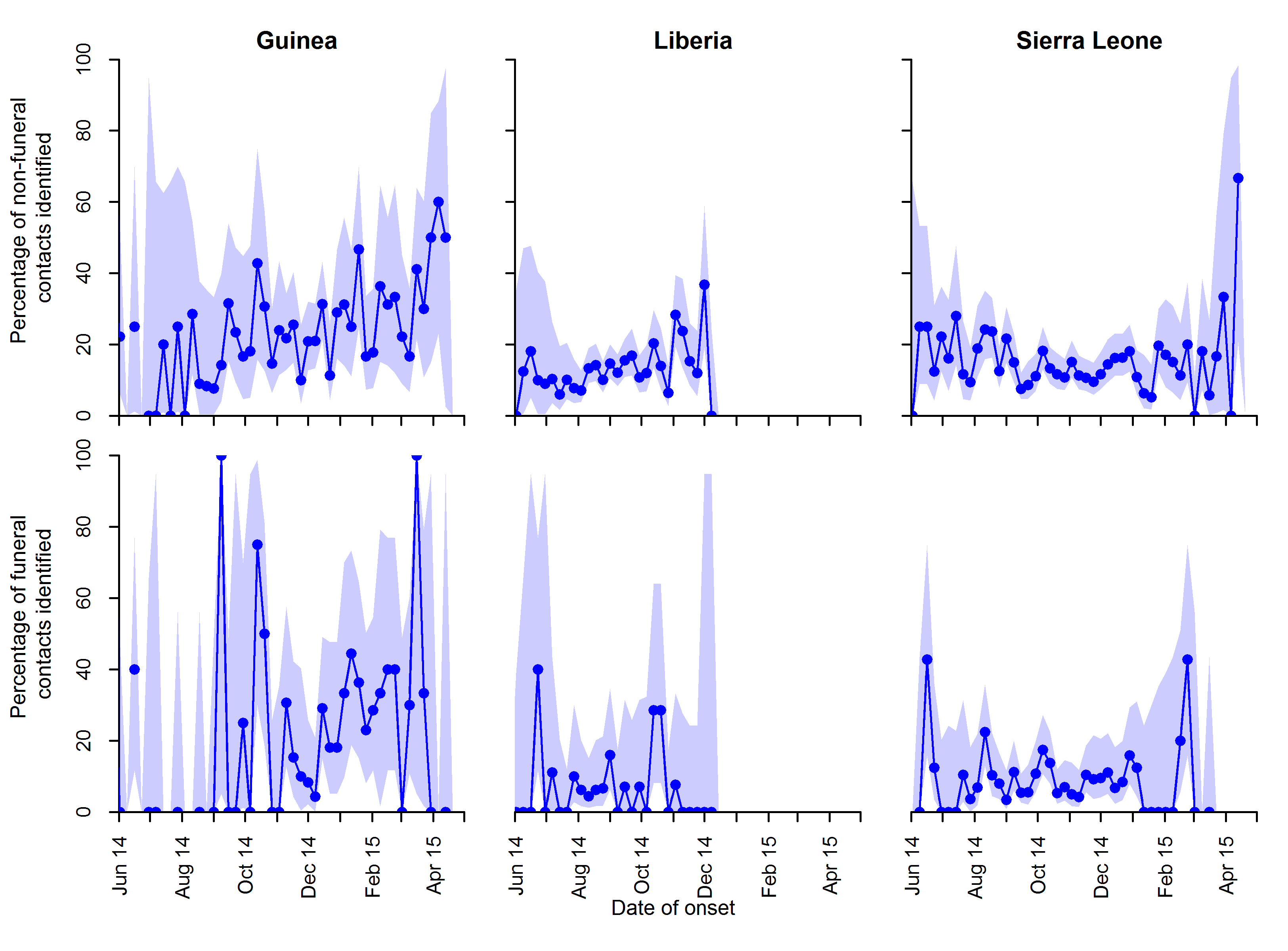


Figure i: Proportion of exposures where the contact has been matched, over time (by week of onset).

## Decreases in the proportions of HCWs and hospitalized cases over time

We found that the relative change in monthly proportions of HCWs among cases after June 2014 (0.79, 95%CI 0.76, 0.82, p<0.001), was significantly larger than that in the monthly proportion of hospitalized cases (0.88, 95%CI 0.87, 0.90, p<0.001), suggesting improved protection of HCWs within the health care facilities over time.

Once adjusting for the decrease of the proportion of hospitalized patients over time, we still found a significant relative change in the monthly proportion of HCWs among cases after June (0.83, 95%CI 0.80, 0.86, p<0.001), confirming our first result.

Results were even more significant when considering the time period from July to November rather than June to November.

## District-level transmission intensity and relationship to funeral exposure and hospitalizations

Table d: Top: Correlation between monthly proportion p of incident cases who reported a funeral exposure among those reporting any exposure and monthly estimated reproduction numbers R, by district (method as previously [[3](#_ENREF_3)]). Bottom: Correlation between monthly proportion p of cases hospitalized within 4 days of symptom onset among all hospitalized cases and monthly estimated reproduction numbers R, by district. The second column presents the main analysis. Columns 1, 3 & 4 show the estimated correlation when restricting the analysis to district months with the denominator of p ≥1, p≥10 and with np>5 & n(1-p)>5, allowing for measurement error. The fifth column presents the “naïve” correlation between the observed p and R, not accounting for uncertainty around the point estimates. The last column presents the correlation as in column 2 and the main analysis, but allows for a different assumption when incorporating measurement error (see “Sensitivity to measurement error assumption” in 1.7 of this S1 Text).

| ***Proportion reporting funeral exposure*** | | | | | | |
| --- | --- | --- | --- | --- | --- | --- |
|  | Estimated correlation for all district-months with ≥ 1cases reporting an exposure | Estimated correlation for all district-months with ≥ 5 cases reporting an exposure | Estimated correlation for all district-months with ≥ 10 cases reporting an exposure | Estimated correlation for all district-months with np>5 and n(1-p)>5 | Naive estimated correlation for all district-months with ≥ 5 cases reporting an exposure | Estimated correlation for all district-months with ≥ 5 cases reporting an exposure (sensitivity to error assumption) |
| Guinea | -0.190 (p=0.229) | -0.098 (p=0.666) | -0.218 (p=0.425) | NA | -0.071 (p=0.661) | -0.055 (p=0.671) |
| Liberia | 0.564 (p=0.004) | 0.546 (p=0.013) | 0.583 (p=0.012) | 0.439 (p=0.143) | 0.428 (p=0.008) | 0.353 (p=0.013) |
| Sierra Leone | 0.534 (p<0.001) | 0.479 (p<0.001) | 0.614 (p<0.001) | 0.546 (p<0.001) | 0.430 (p<0.001) | 0.400 (p<0.001) |
| All countries | 0.249 (p=0.005) | 0.346 (p<0.001) | 0.395 (p<0.001) | 0.393 (p=0.002) | 0.284 (p<0.001) | 0.248 (p<0.001) |
| ***Proportion hospitalized within 4 days*** | | | | | | |
|  | Estimated correlation for all district-months with ≥ 1 hospitalized case | Estimated correlation for all district-months with ≥ 5 hospitalized cases | Estimated correlation for all district-months with ≥ 10 hospitalized cases | Estimated correlation for all district-months with np>5 and n(1-p)>5 | Naive estimated correlation for all district-months with ≥ 5 hospitalized cases | Estimated correlation for all district-months with ≥ 5 hospitalized cases (sensitivity to error assumption) |
| Guinea | -0.303 (p=0.016) | -0.149 (p=0.376) | -0.133 (p=0.479) | -0.575 (p=0.069) | -0.107 (p=0.376) | -0.085 (p=0.376) |
| Liberia | -0.529 (p=0.003) | -0.469 (p=0.039) | -0.367 (p=0.148) | -0.298 (p=0.383) | -0.367 (p=0.033) | -0.303 (p=0.038) |
| Sierra Leone | -0.370 (p=0.011) | -0.565 (p<0.001) | -0.697 (p<0.001) | -0.561 (p=0.018) | -0.447 (p<0.001) | -0.376 (p<0.001) |
| All countries | -0.357 (p<0.001) | -0.369 (p<0.001) | -0.361 (p<0.001) | -0.307 (p=0.031) | -0.293 (p<0.001) | -0.245 (p<0.001) |

The sum of the adjusted r^2^ for the two simple linear models (examining the district level monthly reproduction number as a linear function of either the proportion reporting funeral exposure or the proportion of hospitalized cases who are hospitalized within 4 days without accounting for uncertainty) was 16.6%, and the adjusted r^2^ of the joint linear model was 16.5%, indicating that the joint model was not better than the separate models.


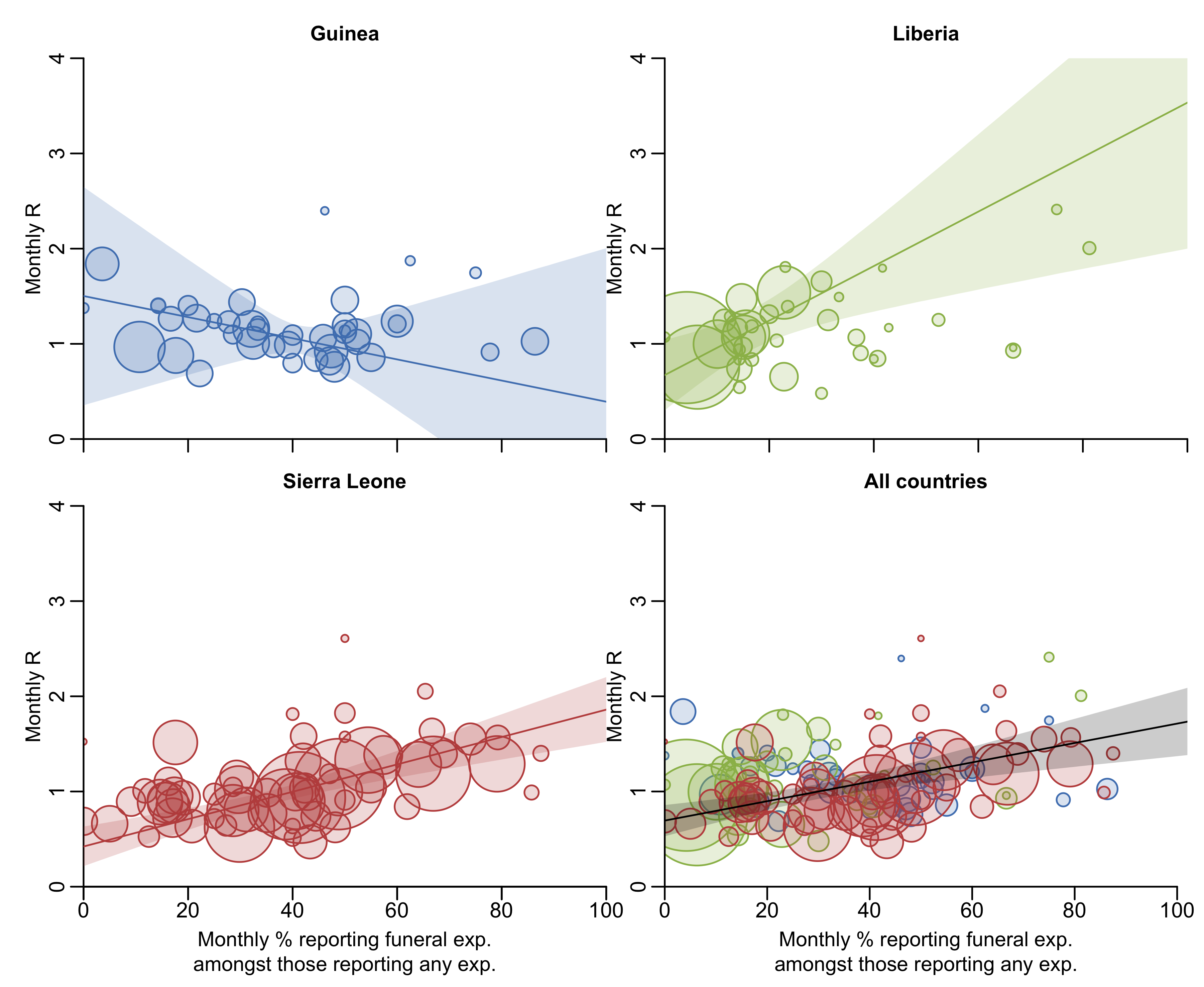


Figure j: Scatter plots of monthly proportion of incident cases who reported funeral exposure among those reporting any exposure and monthly estimated reproduction numbers (method as previously *[*[*1*](#_ENREF_1)*]*). Each point is a district month. We use a weighted regression method which takes account of the uncertainties in the data *[*[*6*](#_ENREF_6)*]*, see methods section; trend lines are shown with confidence intervals. The area of the circle is proportional to the weight of that point (larger points have more weight). The bottom right plot shows a trend line for the whole dataset. The intercepts ($\boldsymbol{\alpha}$) and slopes ($\boldsymbol{\beta}$) for each panel are respectively: Guinea: $\boldsymbol{\alpha}$=1.50 (0.36; 2.65); $\boldsymbol{\beta}$= -1.11 (-3.85; 1.63); Liberia: $\boldsymbol{\alpha}$=0.67 (0.31; 1.04) ; $\boldsymbol{\beta}$=2.86 (1.06; 4.66) ; Sierra Leone: $\boldsymbol{\alpha}$=0.42 (0.22; 0.63) ; $\boldsymbol{\beta}$=1.44 (0.92; 1.96) ; All countries: $\boldsymbol{\alpha}$=0.70 (0.54; 0.85) ; $\boldsymbol{\beta}$=0.99 (0.54; 1.44).


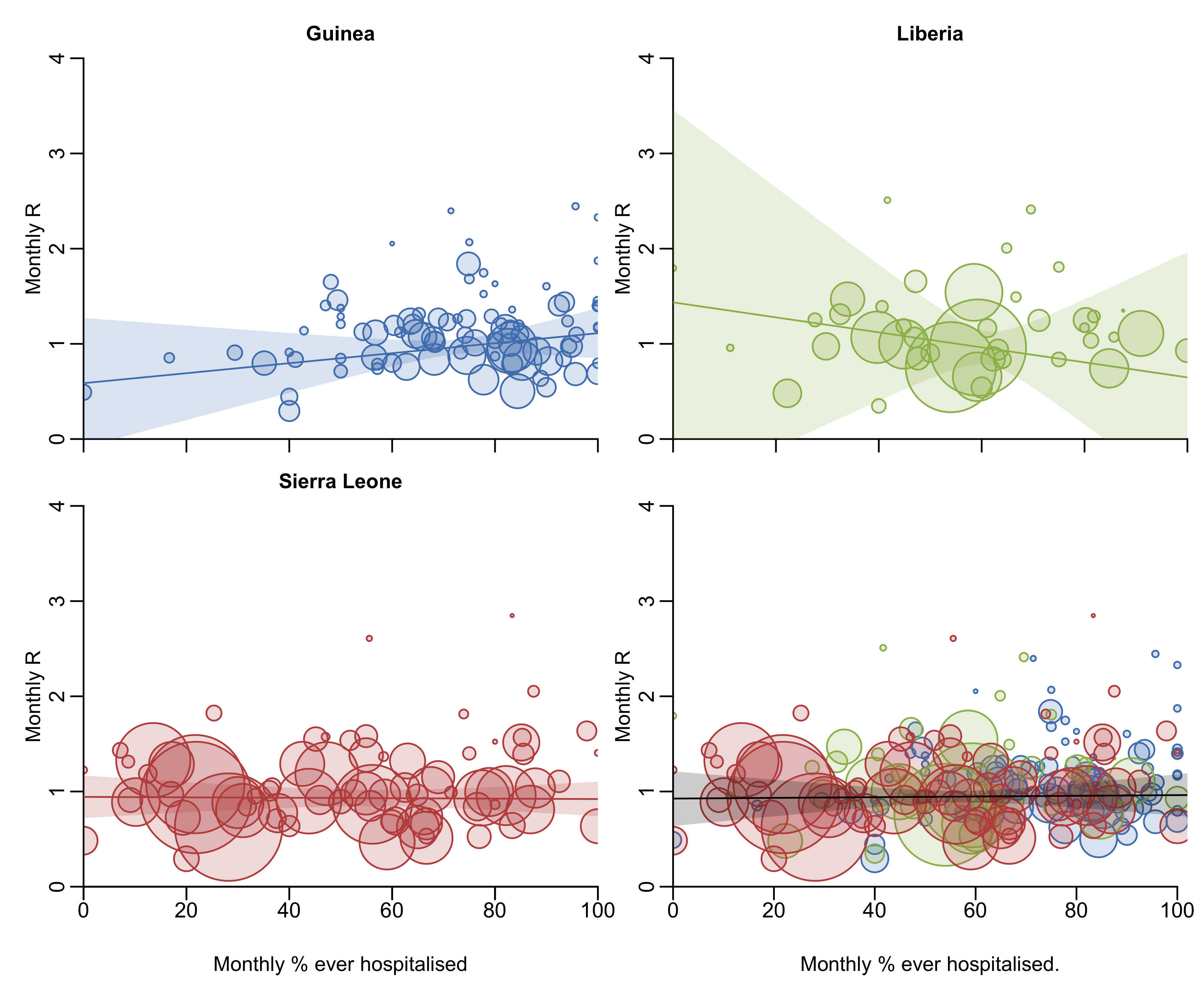


Figure k: Scatter plots of monthly proportion of incident cases hospitalized and monthly estimated reproduction numbers (method as previously *[*[*3*](#_ENREF_3)*]*). Each point is a district month. We use a weighted regression method which takes account of the uncertainties in the data *[*[*6*](#_ENREF_6)*]*, see methods section; trend lines are shown with confidence intervals. The area of the circle is proportional to the weight of that point (larger points have more weight). The bottom right plot shows a trend line for the whole dataset.


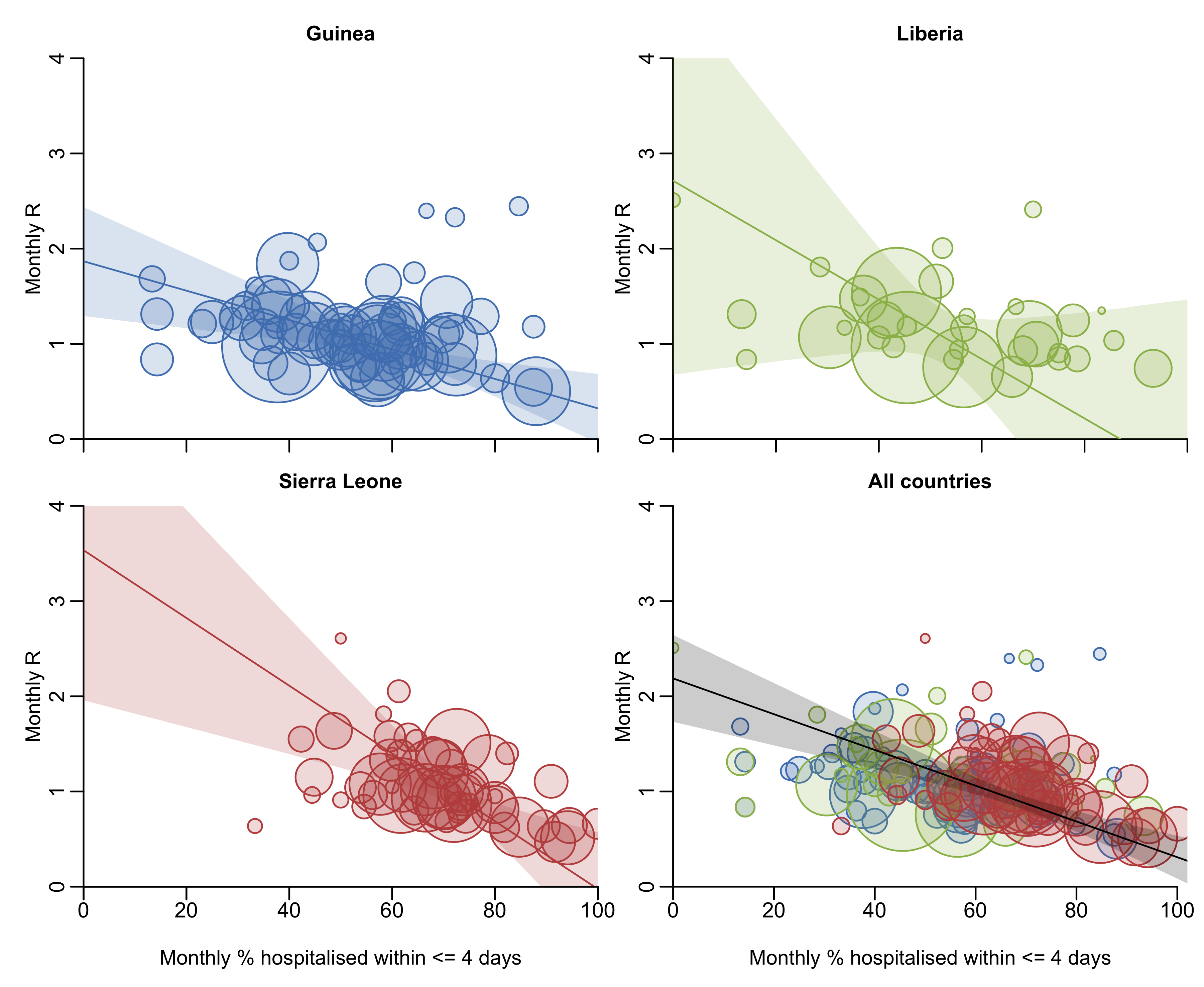


Figure l: Scatter plots of monthly proportion of incident cases hospitalized within <=4 days of symptom onset among those with a reported hospitalization date and monthly estimated reproduction numbers (method as previously *[*[*3*](#_ENREF_3)*]*). Each point is a district month. We use a weighted regression method which takes account of the uncertainties in the data *[*[*6*](#_ENREF_6)*]*, see methods section; trend lines are shown with confidence intervals. The area of the circle is proportional to the weight of that point (larger points have more weight). The bottom right plot shows a trend line for the whole dataset. The intercepts ($\boldsymbol{\alpha}$) and slopes ($\boldsymbol{\beta}$) for each panel are respectively: Guinea: $\boldsymbol{\alpha}$=1.87 (1.30; 2.44); $\boldsymbol{\beta}$=-1.55 (-2.47; -0.62) ; Liberia: $\boldsymbol{\alpha}$=2.71 (0.68; 4.75) ; $\boldsymbol{\beta}$=-3.12 (-6.99; 0.74); Sierra Leone: $\boldsymbol{\alpha}$=3.53 (1.97; 5.10) ; $\boldsymbol{\beta}$=-3.56 (-5.71; -1.41) ; All countries: $\boldsymbol{\alpha}$=2.19 (1.73; 2.65) ; $\boldsymbol{\beta}$=-1.88 (-2.55; -1.21).


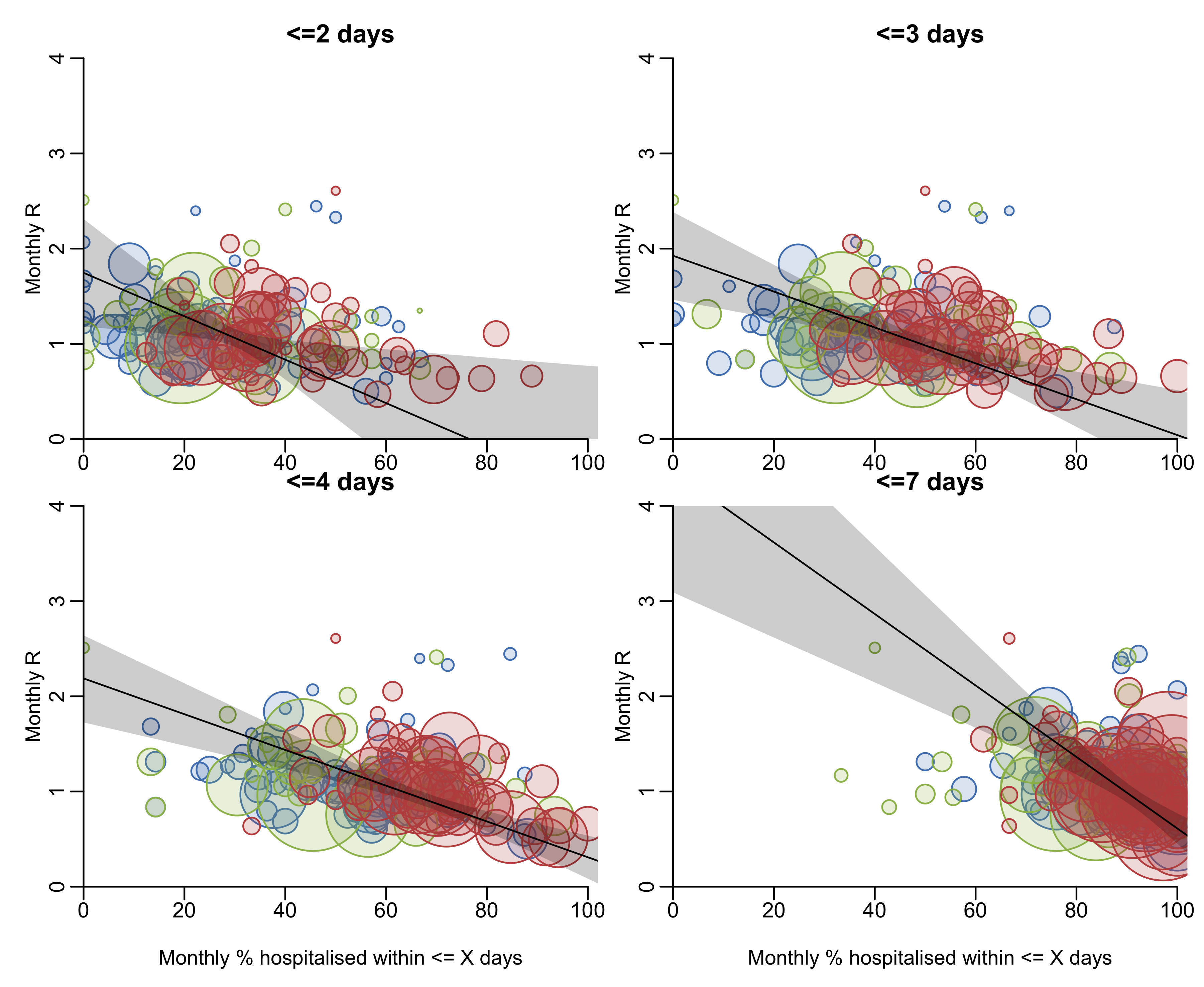


Figure m: Sensitivity analysis around the delay chosen in Figure l. Scatter plots of monthly proportion of incident cases hospitalized within <=2, 3, 4 and 7 days of symptom onset among those with a reported hospitalization date and monthly estimated reproduction numbers (method as previously *[*[*3*](#_ENREF_3)*]*). Each point is a district month. The r^2^ values are 0.06, 0.05, 0.09 and 0.07 respectively.

## Timing of exposure events relative to the clinical timelines of the contact

Figure n: A) Distribution of reported duration of exposures by country B) Observed and fitted distribution of reported exposure times relative to onset (top), death (middle) and hospitalization (bottom) of the contact. The mid-point of the exposure interval is used to generate the histograms, colored by health center type for the contact (left) or by relationship to the contact (right). The red curves show the best fits for the ‘signal’ distribution.


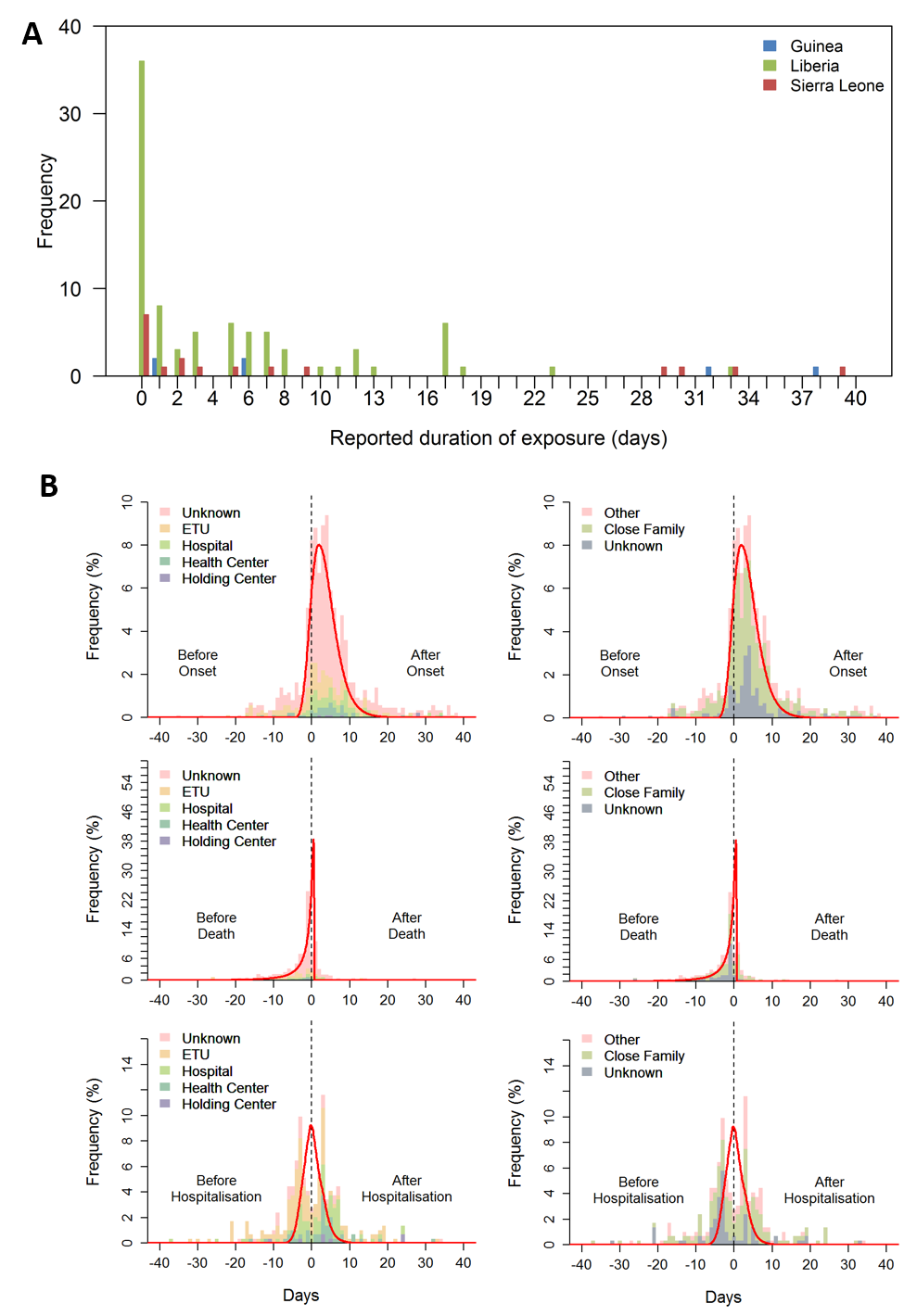


Table e: Table of the estimated parameters for the distributions of the timing of exposure events relative to the clinical timelines of the contact. The distributions are all offset log-normal distributions, the distribution representing the “Signal” is more peaked than the distribution representing the “Noise”. P_1_ is the relative weight of the signal distribution. Note that the distribution relative to death is reversed in time. Estimates shown are median (95% CI).

|  |  | **Signal distribution** | | | **Noise distribution** | | |
| --- | --- | --- | --- | --- | --- | --- | --- |
|  | **p_1_** | **Mean** | **Variance** | **Offset** | **Mean** | **Variance** | **Offset** |
| Onset | 0.694 (0.5646-0.7107) | 9.02 (3.025-9.02) | 109 (9.306-109) | 2.871 (2.057-376.4) | 2.424 (0.815-7.056) | 171.1 (122.5-275.6) | 277 (141.7-466.7) |
| Death | 0.7401 (0.6675-0.8138) | 3.063 (2.2-4.257) | 76.38 (32.48-279.9) | 0.6546 (0.5643-0.8282) | 0.1817 (-3.696-3.228) | 210.7 (148.1-326.1) | 176.2 (150.3-195.9) |
| Hospitalization | 0.549 (0.3265-0.9585) | 0.3457 (-0.5435-2.621) | 7.066 (0.4134-79.91) | 19.05 (0.6007-1746) | -1.485 (-28.48-1.349) | 192.1 (131.5-314.5) | 248.3 (52.21-1176) |

## Relative importance of onset and death in the timing of exposure events


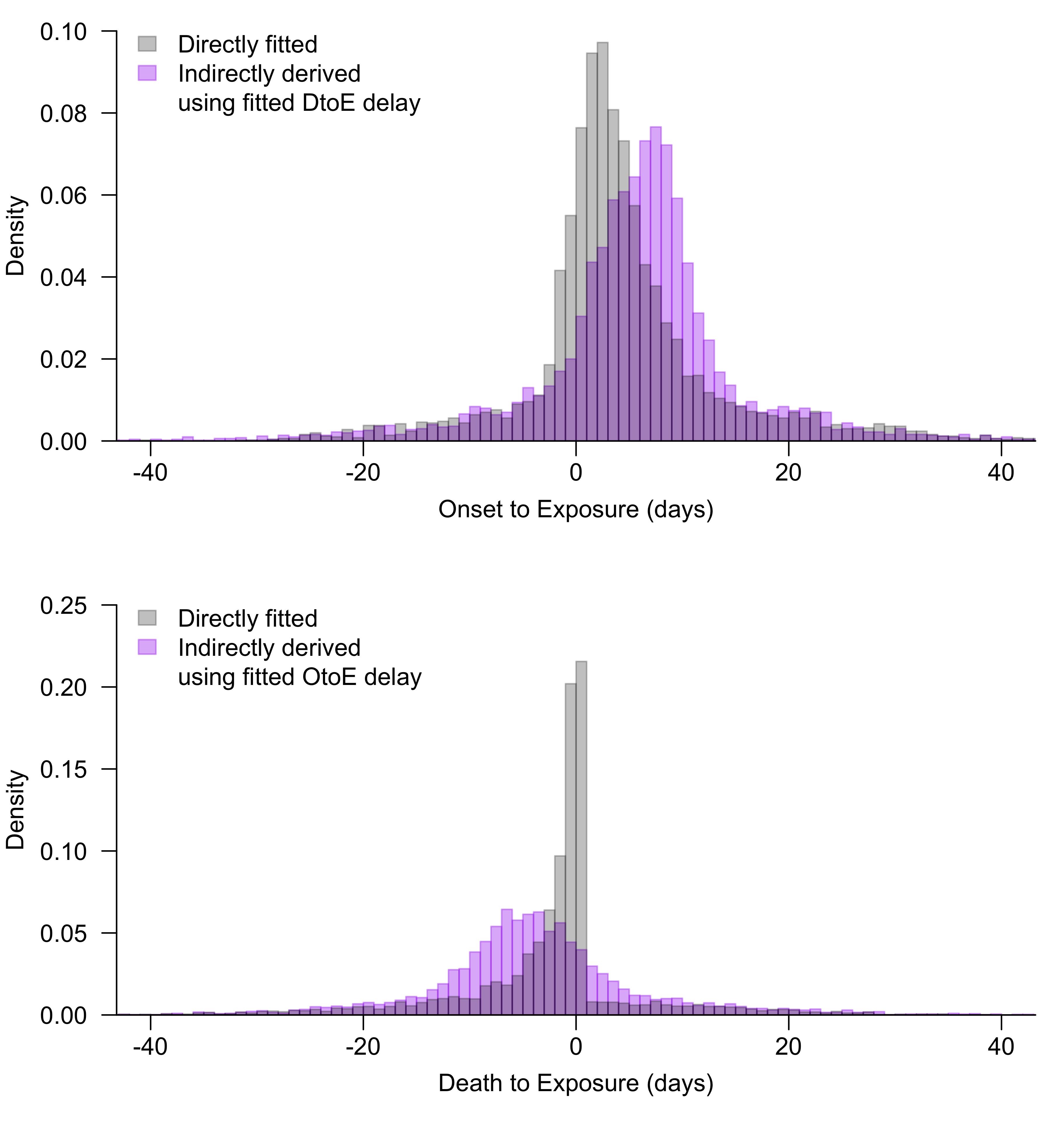


Figure o: Comparing directly fitted and indirectly derived Onset-to-Exposure (OtoE) and Death-to-Exposure (DtoE) delays. The top graph shows that using the fitted DtoE delay to indirectly derive the OtoE delay produces a good match to the directly fitted OtoE delay (Kullback Leibler divergence 0.20). The bottom graph shows that using the fitted OtoE delay to indirectly derive the DtoE delay produces a poor match to the directly fitted DtoE delay (Kullback Leibler divergence 0.50). This suggests that the timing of death is more influential than that of onset in determining when onwards exposure occurs.

##

## Analysis of the network of reported exposures

Figure p: The network of all cases and the contacts they have named as having been exposed to. Individuals (case and contacts) are shown as nodes, and exposures as directed arrows from contacts to cases. If multiple exposures occurred between a cases and a contact, only one arrow is shown. All individuals are Confirmed or Probable Ebola cases. Arrows are red for funeral exposures, black for non-funeral exposures and blue for multiple exposures involving both non-funeral and funeral exposures. Square nodes are males, round nodes females, and triangles unknown. Red nodes are case known to have died, blue nodes are cases known to have survived and grey nodes are cases with no recorded outcome.


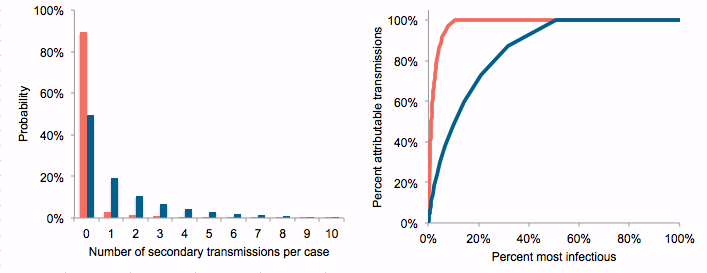


Figure q: The inferred offspring distribution. Blue: conservative assumptions, based on a negative binomial distribution with mean 1.5 and k=0.52; Red: less conservative assumptions, based on a negative binomial distribution with mean 1.0 and k=0.03. Left, the probability mass function for these distributions, and right, the proportion of transmissions attributable to the x% most infectious, as a function of x%, for these two distributions (also shown in Figure 2 panel B).

Table f: Comparison of best-fit model to non-funeral exposures (distribution of number of times a person is named as a non-funeral contact).

| **Model** | **Number of Parameters** | **- Log Likelihood** | **Akaike’s information criterion (AIC)** | **Modified AICc** | **Parameter 1** | **Parameter 2** |
| --- | --- | --- | --- | --- | --- | --- |
| Poisson | 1 | 620.2 | 1242.3 | 1242.3 | 0.80 |  |
| Geometric | 1 | 578.7 | 1159.3 | 1159.3 | 0.69 |  |
| Negative Binomial | 2 | 566.1 | 1136.2 | 1136.2 | 0.51 | 0.001* |
| Logarithmic | 1 | 566.1 | 1134.2 | 1134.2 | 0.51 (0.45-0.57)** |  |
| Zipf | 1 | 566.5 | 1135.0 | 1135.0 | 2.53 |  |

*****Smallest value considered. For the negative binomial model, the distribution becomes approximately equal to the logarithmic distribution for small values of the second parameter.

** 95% Confidence intervals are reported only for the selected distribution

Table g: Comparison of best-fit model to funeral exposures (distribution of number of times a person is named as a funeral contact, or both as a non-funeral and a funeral contact by the same person).

| **Model** | **Number of Parameters** | **- Log Likelihood** | **Akaike’s information criterion (AIC)** | **Modified AICc** | **Parameter 1** | **Parameter 2** |
| --- | --- | --- | --- | --- | --- | --- |
| Poisson | 1 | 156.2 | 314.3 | 314.3 | 0.77 |  |
| Geometric | 1 | 147.8 | 297.6 | 297.6 | 0.70 |  |
| Negative Binomial | 2 | 145.4 | 294.8 | 294.8 | 0.49 | 0.001* |
| Logarithmic | 1 | 145.4 | 292.8 | 292.8 | 0.49 (0.39-0.61)^**^ |  |
| Zipf | 1 | 143.9 | 289.8 | 289.8 | 2.41 |  |

*****As above.

** 95% Confidence intervals are reported only for the selected distribution

Table h: Comparison of best-fit model to all exposures combined (distribution of number of times a person is named as a contact). The line corresponding to the distribution shown in Fig 2A is shown in bold.

| **Model** | **Number of Parameters** | **- Log Likelihood** | **Akaike’s information criterion (AIC)** | **Modified AICc** | **Parameter 1** | **Parameter 2** |
| --- | --- | --- | --- | --- | --- | --- |
| Poisson | 1 | 109.5 | 221.0 | 221.0 | 0.87 |  |
| Geometric | 1 | 106.9 | 215.9 | 215.9 | 0.67 |  |
| Negative Binomial | 2 | 106.9 | 217.8 | 217.9 | 0.38 | 0.70^*^ |
| **Logarithmic** | **1** | **107.4** | **216.8** | **216.9** | **0.53 (0.40-0.67)^**^** |  |
| Zipf | 1 | 108.8 | 219.6 | 219.7 | 2.18 |  |

*****As above.

** 95% Confidence intervals are reported only for the selected distribution

## Case-contact pairs by age and sex

We examined the matched case-contact pairs to determine if there were any age or sex related reporting patterns. Age of cases was recorded in years (or months for infants). In the following analyses, we define age groups into children (<=15 year olds), young adults (16-44) and older adults (>=45). The two adult groups are sometimes merged to allow sufficient sample sizes.

Males were more likely than females to be named by males; this was particularly true in Guinea, Table i. The majority of children (<=15 year olds) are named by adults aged 16-44 (Table j).

Table i: The type of pairs by sex, with percentages per country in brackets. Only in Guinea do males have a significantly higher chance than females of being named by males. NC = Named Contact.

|  | | | **Case naming contact** | | **OR [95%CI]** |
| --- | --- | --- | --- | --- | --- |
|  |  |  | **Male** | **Female** |  |
| **NC** | **All** | **Male** | 264 (26%) | 277 (27.3%) | 1.182 [0.923, 1.515] |
|  |  | **Female** | 212 (20.9%) | 263 (25.9%) |  |
|  | **Guinea** | **Male** | 43 (25.7%) | 45 (26.9%) | 1.648 [0.89, 3.081] |
|  |  | **Female** | 29 (17.4%) | 50 (29.9%) |  |
|  | **Sierra Leone** | **Male** | 146 (28.6%) | 135 (26.4%) | 1.287 [0.908, 1.828] |
|  |  | **Female** | 105 (20.5%) | 125 (24.5%) |  |
|  | **Liberia** | **Male** | 71 (21.7%) | 90 (27.5%) | 0.89 [0.575, 1.376] |
|  |  | **Female** | 78 (23.9%) | 88 (26.9%) |  |

Table j: The type of pairs by age group. The percentages along rows sum to 100%, indicating what percentage of that age group was the named contact (potential infector) to an Ebola case.

|  | | | **Case naming contact** | | |
| --- | --- | --- | --- | --- | --- |
|  |  |  | **<=15** | **16-44** | **>=45** |
| **Named contact** | **All** | **<=15** | 22 (25.6%) | 48 (55.8%) | 16 (18.6%) |
|  |  | **16-44** | 143 (26.1%) | 304 (55.5%) | 101 (18.4%) |
|  |  | **>=45** | 58 (16.7%) | 203 (58.5%) | 86 (24.8%) |
|  | **Guinea** | **<=15** | 4 (36.4%) | 2 (18.2%) | 5 (45.5%) |
|  |  | **16-44** | 10 (12.8%) | 48 (61.5%) | 20 (25.6%) |
|  |  | **>=45** | 5 (6.6%) | 48 (63.2%) | 23 (30.3%) |
|  | **Sierra Leone** | **<=15** | 7 (18.9%) | 25 (67.6%) | 5 (13.5%) |
|  |  | **16-44** | 77 (25.7%) | 175 (58.3%) | 48 (16%) |
|  |  | **>=45** | 35 (22.4%) | 88 (56.4%) | 33 (21.2%) |
|  | **Liberia** | **<=15** | 11 (28.9%) | 21 (55.3%) | 6 (15.8%) |
|  |  | **16-44** | 56 (35.2%) | 72 (45.3%) | 31 (19.5%) |
|  |  | **>=45** | 18 (15.7%) | 67 (58.3%) | 30 (26.1%) |

## Predictors of being a named contact

### Non-funeral

Table k: Predictors of being a named non-funeral contact (logistic regression) multivariable analysis: All Countries. The most parsimonious yet adequate model (determined by the AIC, see methods section) is presented. Covariates that do not contribute to this model are not presented. Univariable regression results for this and subsequent regressions are available on request.

| **Category** | **Name** | **Odds Ratio** | **95% CI** | **p** |
| --- | --- | --- | --- | --- |
| Case Definition | Suspected | Ref |  |  |
|  | Confirmed | 2.45 | 2.01 - 2.99 | <0.001 |
|  | Probable | 1.91 | 1.51 - 2.41 | <0.001 |
| Fever | No | Ref |  |  |
|  | Yes | 1.52 | 1.20 - 1.95 | 0.001 |
|  | Missing | 2.04 | 1.45 - 2.88 | <0.001 |
| Unconscious | No | Ref |  |  |
|  | Yes | 1.98 | 1.26 - 3.03 | 0.002 |
|  | Missing | 1.79 | 0.95 - 3.28 | 0.064 |
| Confused | No | Ref |  |  |
|  | Yes | 0.60 | 0.40 - 0.86 | 0.007 |
|  | Missing | 0.33 | 0.18 - 0.63 | 0.001 |
| Respiratory symptoms | No | Ref |  |  |
|  | Yes | 1.18 | 1.00 - 1.40 | 0.055 |
|  | Missing | 0.93 | 0.68 - 1.28 | 0.662 |
| Other symptoms | No | Ref |  |  |
|  | Yes | 0.96 | 0.69 - 1.36 | 0.818 |
|  | Missing | 0.56 | 0.36 - 0.89 | 0.012 |
| Bleeding symptoms | No | Ref |  |  |
|  | Yes | 1.40 | 1.07 - 1.81 | 0.013 |
|  | Missing | 1.00 | 0.79 - 1.26 | 0.977 |
| Age group | < 16 yrs | Ref |  |  |
|  | ≥ 16 yrs | 1.87 | 1.52 - 2.34 | <0.001 |
|  | Missing | 1.84 | 1.16 - 2.84 | 0.007 |
| Hospitalized | No | Ref |  |  |
|  | Yes ETU | 0.62 | 0.50 - 0.77 | <0.001 |
|  | Yes not ETU | 0.79 | 0.62 - 1.01 | 0.059 |
|  | Yes, unknown hospital type | 0.92 | 0.74 - 1.13 | 0.410 |
|  | Missing | 0.75 | 0.58 - 0.95 | 0.021 |
| Number of days between report and death++ | ≥ 0 | Ref |  |  |
|  | < 0 | 1.18 | 0.94 - 1.47 | 0.147 |
|  | Missing | 0.84 | 0.65 - 1.08 | 0.168 |
| Country | Guinea | Ref |  |  |
|  | Liberia | 0.98 | 0.75 - 1.29 | 0.892 |
|  | Sierra Leone | 0.73 | 0.56 - 0.94 | 0.014 |
| Final Outcome | Alive | Ref |  |  |
|  | Dead | 2.04 | 1.50 - 2.79 | <0.001 |
|  | Missing | 1.11 | 0.85 - 1.46 | 0.449 |
| Travelled+ | No | Ref |  |  |
|  | Yes | 1.73 | 1.35 - 2.18 | <0.001 |
| Date of Report | Dec13-May14 | 2.12 | 1.35 - 3.25 | 0.001 |
|  | Jun14-Nov14 | Ref |  |  |
|  | Dec14-May15 | 0.82 | 0.67 - 1.01 | 0.066 |
|  | Missing | 0.79 | 0.59 - 1.05 | 0.114 |

+ The case reported travelling outside their home or village/town before becoming ill

++ A negative number means death preceded reporting of the case

### Funeral

Table l: Predictors of being a named funeral contact (logistic regression) multivariable analysis: All countries. The most parsimonious yet adequate model (determined by the AIC, see methods section) is presented. Covariates that do not contribute to this model are not presented.

| **Category** | **Name** | **Odds Ratio** | **95% CI** | **p** |
| --- | --- | --- | --- | --- |
| Case Definition | Suspected | Ref |  |  |
|  | Confirmed | 1.98 | 1.28 - 3.11 | 0.002 |
|  | Probable | 2.03 | 1.21 - 3.42 | 0.008 |
| Fever | No | Ref |  |  |
|  | Yes | 1.81 | 1.08 - 3.18 | 0.030 |
|  | Missing | 1.45 | 0.74 - 2.95 | 0.288 |
| Unconscious | No | Ref |  |  |
|  | Yes | 1.56 | 0.66 - 3.22 | 0.264 |
|  | Missing | 0.65 | 0.41 - 1.04 | 0.074 |
| Respiratory symptoms | No | Ref |  |  |
|  | Yes | 1.65 | 1.09 - 2.54 | 0.020 |
|  | Missing | 1.63 | 0.91 - 2.93 | 0.099 |
| Age group | < 16 yrs | Ref |  |  |
|  | ≥ 16 yrs | 2.44 | 1.47 - 4.36 | 0.001 |
|  | Missing | 2.94 | 0.93 - 7.88 | 0.043 |
| Hospitalized | No | Ref |  |  |
|  | Yes ETU | 0.18 | 0.09 - 0.34 | <0.001 |
|  | Yes not ETU | 0.49 | 0.26 - 0.87 | 0.019 |
|  | Yes, unknown hospital type | 0.56 | 0.34 - 0.89 | 0.018 |
|  | Missing | 0.44 | 0.27 - 0.71 | 0.001 |
| Number of days between report and death++ | ≥ 0 | Ref |  |  |
|  | < 0 | 1.64 | 1.12 - 2.40 | 0.010 |
|  | Missing | 1.09 | 0.65 - 1.78 | 0.729 |
| Country | Guinea | Ref |  |  |
|  | Liberia | 0.25 | 0.13 - 0.47 | <0.001 |
|  | Sierra Leone | 0.63 | 0.38 - 1.05 | 0.077 |
| Travelled+ | No | Ref |  |  |
|  | Yes | 2.47 | 1.50 - 3.90 | <0.001 |
| Date of Report | Dec13-May14 | 2.43 | 1.05 - 5.23 | 0.029 |
|  | Jun14-Nov14 | Ref |  |  |
|  | Dec14-May15 | 0.61 | 0.39 - 0.93 | 0.024 |
|  |  |  |  |  |
|  | Missing | 0.68 | 0.30 - 1.44 | 0.324 |

+ The case reported travelling outside their home or village/town before becoming ill

++ A negative number means death preceded reporting of the case

### Impact of hospitalization status on onwards transmission

**Table m: Odds ratio of being named as a non-funeral contact for hospitalized compared with non-hospitalized CP cases, overall, before 15^th^ August 2014, between 15^th^ August 2014 and 30 November 2014 and after 30 November 2014, and by country.**

|  | **OR (95% CI)** | **p-value** |
| --- | --- | --- |
| Overall | 0.78 ( 0.66 - 0.92 ) | 0.004 |
| Before 15^th^ August | 0.73 ( 0.49 - 1.09 ) | 0.113 |
| Between 15^th^ August and 30^th^ November | 0.76 ( 0.61 - 0.95 ) | 0.017 |
| December 2014 onwards | 0.71 ( 0.49 - 1.02 ) | 0.061 |
| Guinea | 0.36 ( 0.24 - 0.54 ) | <0.001 |
| Liberia | 0.78 ( 0.59 - 1.04 ) | 0.090 |
| Sierra Leone | 0.90 ( 0.70 - 1.16 ) | 0.416 |
| Guinea, before 15^th^ August | 0.38 ( 0.18 - 0.86 ) | 0.015 |
| Guinea, between 15^th^ Aug and 30^th^ Nov | 0.18 ( 0.08 - 0.47 ) | <0.001 |
| Guinea, from 1^st^ Dec onwards | 0.53 ( 0.31 - 0.94 ) | 0.026 |
| Liberia, before 15^th^ August | 0.71 ( 0.40 - 1.29 ) | 0.246 |
| Liberia, between 15^th^ Aug and 30^th^ Nov | 0.82 ( 0.59 - 1.14 ) | 0.230 |
| Liberia, from 1^st^ Dec onwards | 0.36 ( 0.04 - 3.23 ) | 0.332 |
| Sierra Leone, before 15^th^ August | 1.32 ( 0.62 - 3.28 ) | 0.504 |
| Sierra Leone, between 15^th^ Aug and 30^th^ Nov | 0.83 ( 0.60 - 1.17 ) | 0.282 |
| Sierra Leone, from 1^st^ Dec onwards | 0.63 ( 0.37 - 1.05 ) | 0.080 |

**Table n: Odds ratio of being named as a funeral contact for hospitalized compared with non-hospitalized CP cases, overall, before 15^th^ August 2014, between 15^th^ August 2014 and 30 November 2014 and after 30 November 2014, and by country.**

|  | **OR (95% CI)** | **p-value** |
| --- | --- | --- |
| Overall | 0.43 ( 0.29 - 0.63 ) | <0.001 |
| Before 15^th^ August | 0.46 ( 0.21 - 1.07 ) | 0.060 |
| Between 15^th^ August and 30^th^ November | 0.40 ( 0.23 - 0.69 ) | 0.001 |
| December 2014 onwards | 0.44 ( 0.20 - 0.93 ) | 0.038 |
| Guinea | 0.28 ( 0.15 - 0.55 ) | <0.001 |
| Liberia | 0.15 ( 0.04 - 0.44 ) | 0.001 |
| Sierra Leone | 0.77 ( 0.44 - 1.31 ) | 0.330 |
| Guinea, before 15^th^ August | 0.41 ( 0.11 - 1.64 ) | 0.176 |
| Guinea, between 15^th^ Aug and 30^th^ Nov | 0.12 ( 0.03 - 0.59 ) | 0.004 |
| Guinea, from 1^st^ Dec onwards | 0.42 ( 0.17 - 1.05 ) | 0.062 |
| Liberia, before 15^th^ August | 0.17 ( 0.02 - 0.79 ) | 0.035 |
| Liberia, between 15^th^ Aug and 30^th^ Nov | 0.14 ( 0.02 - 0.59 ) | 0.014 |
| Liberia, from 1^st^ Dec onwards* | NA | NA |
| Sierra Leone, before 15^th^ August | 1.51 ( 0.26- 28.50 ) | 0.701 |
| Sierra Leone, between 15^th^ Aug and 30^th^ Nov | 0.88 ( 0.45 - 1.69 ) | 0.705 |
| Sierra Leone, from 1^st^ Dec onwards | 0.17 ( 0.01 - 0.91 ) | 0.093 |

*Numbers too small to estimate the OR

**Table o: Sensitivity analysis (see methods): Odds ratio of being named as a funeral contact versus being named as a non-funeral contact, for hospitalized compared with non-hospitalized cases, overall, before 15^th^ August 2014, between 15^th^ August 2014 and 30 November 2014 and after 30 November 2014, and by country.**

|  | **OR (95% CI)** | **p-value** |
| --- | --- | --- |
| Overall | 0.60 (0.44 - 0.82) | 0.002 |
| Before 15^th^ August | 0.54 (0.25 - 1.20) | 0.131 |
| Between 15^th^ August and 30^th^ November | 0.60 (0.40 - 0.91) | 0.017 |
| December 2014 onwards | 0.72 (0.38 - 1.40) | 0.326 |
| Guinea | 0.44 (0.24 - 0.82) | 0.011 |
| Liberia | 0.26 (0.10 - 0.69) | 0.005 |
| Sierra Leone | 0.82 (0.54 - 1.20) | 0.332 |
| Guinea, before 15^th^ August | 0.55 (0.15 - 2.10) | 0.398 |
| Guinea, between 15^th^ Aug and 30^th^ Nov | 0.26 (0.08 - 0.88) | 0.038 |
| Guinea, from 1^st^ Dec onwards | 0.56 (0.23 - 1.30) | 0.197 |
| Liberia, before 15^th^ August | 0.30 (0.07 - 1.30) | 0.120 |
| Liberia, between 15^th^ Aug and 30^th^ Nov | 0.22 (0.06 - 0.83) | 0.021 |
| Liberia, from 1^st^ Dec onwards* | NA | NA |
| Sierra Leone, before 15^th^ August | 0.86 (0.16 - 4.60) | 0.827 |
| Sierra Leone, between 15^th^ Aug and 30^th^ Nov | 0.81 (0.49 - 1.30) | 0.409 |
| Sierra Leone, from 1^st^ Dec onwards | 0.59 (0.18 - 1.90) | 0.380 |

*Numbers too small to estimate the OR

## Predictors of being a multiply named contact

### Non-funeral

Table p: Predictors of being named multiple times as non-funeral exposure (Negative Binomial regression) multivariable analysis. The most parsimonious yet adequate model (determined by the AIC, see methods section) is presented. Covariates that do not contribute to this model are not presented.

| **Category** | **Name** | **Relative number of times being named** | **95% CI** | **p** |
| --- | --- | --- | --- | --- |
| Bleeding symptoms | No | Ref |  |  |
|  | Yes | 1.67 | 1.07 - 2.64 | 0.025 |
|  | Missing | 0.80 | 0.57 - 1.13 | 0.191 |
| Hospitalized | No | Ref |  |  |
|  | Yes ETU | 0.50 | 0.33 - 0.73 | 0.001 |
|  | Yes not ETU | 0.60 | 0.38 - 0.96 | 0.034 |
|  | Yes, unknown hospital type | 0.70 | 0.48 - 1.04 | 0.078 |
|  | Missing | 0.89 | 0.57 - 1.40 | 0.612 |
| Travelled+ | No | Ref |  |  |
|  | Yes | 1.66 | 1.10 - 2.52 | 0.017 |
| HCW | No | Ref |  |  |
|  | Yes | 0.48 | 0.23 - 0.96 | 0.044 |

+ The case reported travelling outside their home or village/town before becoming ill

### Funeral

Table q: Predictors of being named multiple times as funeral exposure (Negative Binomial regression) multivariable analysis. The most parsimonious yet adequate model (determined by the AIC, see methods section) is presented. Covariates that do not contribute to this model are not presented.

| **Category** | **Name** | **Relative number of times being named** | **95% CI** | **p** |
| --- | --- | --- | --- | --- |
| Travelled | No | Ref |  |  |
|  | Yes | 4.41 | 2.01 - 10.55 | <0.001 |

+ The case reported travelling outside their home or village/town before becoming ill

# Sensitivity Analysis for Case Definition

The results presented in the main text and S1 Text so far define an Ebola case as *Confirmed or Probable (CP)*. We removed *Suspected* cases from the linelist.

However, within country use of these definitions has varied. For example, Guinea reports very few Suspected cases. Also, cases can be re-classified from Suspected to Probable or Confirmed, or from Probable to Confirmed as more information is obtained (eg, test results).

Due to wide uncertainty as to whether Suspected cases are truly infected with Ebola we performed some sensitivity analysis using Confirmed, Probable and Suspected (CPS) cases.

## Numbers of CPS cases and exposures

The proportion of CPS cases reporting at least one exposure is 25.8% (vs 32.6% for CP cases (Table r). The proportion of matched CPS-CPS exposures was 16.6% (vs 13.9% for CP-CP exposures). The distributions of exposures and matched exposures do not vary substantially by country compared to the CP equivalents (although Guinea reports fewer than 20 Suspected cases in the dataset we analysed, so their numbers largely remain unchanged, Table r).

Table r: Number of Confirmed, Probable and Suspected (CPS) cases, exposures and matched CPS-CPS contacts. Equivalent of Table 1 in main text

|  | **All** | **Guinea** | **Liberia** | **Sierra Leone** |
| --- | --- | --- | --- | --- |
| Total cases | 29872 | 3543 | 8130 | 18199 |
| Cases reporting an exposure | 7713 (25.8%) | 894 (25.2%) | 2449 (30.1%) | 4370 (24%) |
| Only Non-Funeral | 4933 (64%) | 572 (64%) | 2013 (82.2%) | 2348 (53.7%) |
| Only Funeral | 355 (4.6%) | 41 (4.6%) | 73 (3%) | 241 (5.5%) |
| Both | 2425 (31.4%) | 281 (31.4%) | 363 (14.8%) | 1781 (40.8%) |
| Total reported exposures | 11751 | 1368 | 3292 | 7091 |
| Funeral reported | 2964 (25.2%) | 326 (23.8%) | 474 (14.4%) | 2164 (30.5%) |
| Non Funeral reported | 8787 (74.8%) | 1042 (76.2%) | 2818 (85.6%) | 4927 (69.5%) |
| Total matched exposures | 1956 (16.6%) | 319 (23.3%) | 633 (19.2%) | 1004 (14.2%) |
| Funeral matched | 384 (19.6%) | 68 (21.3%) | 74 (11.7%) | 242 (24.1%) |
| Non Funeral matched | 1572 (80.4%) | 251 (78.7%) | 559 (88.3%) | 762 (75.9%) |
| Total number of matched contacts^*^ | 1034 | 163 | 372 | 499 |
| ***Details about type of exposure*** | | | | |
| Funeral, reported type of exposure | 2031 (68.5%) | 217 (66.6%) | 311 (65.6%) | 1503 (69.5%) |
| Touched corpse | 1305 (64.3%) | 154 (71%) | 189 (60.8%) | 962 (64%) |
| Did not touch corpse | 726 (35.7%) | 63 (29%) | 122 (39.2%) | 541 (36%) |
| Non Funeral, reported type of exposure | 2787 (31.7%) | 102 (9.8%) | 1570 (55.7%) | 1115 (22.6%) |
| Belongings | 1593 (57.2%) | 30 (29.4%) | 828 (52.7%) | 735 (65.9%) |
| Bodily fluids | 1517 (54.4%) | 35 (34.3%) | 794 (50.6%) | 688 (61.7%) |
| Household | 1081 (38.8%) | 31 (30.4%) | 535 (34.1%) | 515 (46.2%) |
| Direct physical | 2422 (86.9%) | 72 (70.6%) | 1383 (88.1%) | 967 (86.7%) |
| ***Relationship between case and contact*** | | | | |
| Funeral, reported a relationship | 2481 (83.7%) | 53 (16.3%) | 424 (89.5%) | 2004 (92.6%) |
| Close family | 1368 (55.1%) | 34 (64.2%) | 222 (52.4%) | 1112 (55.5%) |
| Extended family | 667 (26.9%) | 11 (20.8%) | 114 (26.9%) | 542 (27%) |
| Friend | 162 (6.5%) | 1 (1.9%) | 64 (15.1%) | 97 (4.8%) |
| Neighbor | 225 (9.1%) | 1 (1.9%) | 12 (2.8%) | 212 (10.6%) |
| Health care | 6 (0.2%) | 0 (0%) | 0 (0%) | 6 (0.3%) |
| Other | 53 (2.1%) | 6 (11.3%) | 12 (2.8%) | 35 (1.7%) |
| Non Funeral, reported a relationship | 7439 (84.7%) | 242 (23.2%) | 2610 (92.6%) | 4587 (93.1%) |
| Close family | 4372 (58.8%) | 148 (61.2%) | 1543 (59.1%) | 2681 (58.4%) |
| Extended family | 1720 (23.1%) | 48 (19.8%) | 553 (21.2%) | 1119 (24.4%) |
| Friend | 445 (6%) | 10 (4.1%) | 234 (9%) | 201 (4.4%) |
| Neighbor | 562 (7.6%) | 12 (5%) | 128 (4.9%) | 422 (9.2%) |
| Health care | 127 (1.7%) | 6 (2.5%) | 52 (2%) | 69 (1.5%) |
| Other | 213 (2.9%) | 18 (7.4%) | 100 (3.8%) | 95 (2.1%) |

^*^ Cases who were named as contacts multiple times are only counted once.

## Main text figures, for the subset of data with Confirmed, Probable or Suspected (CPS) case definition

We examined how the main text results changed if we included Confirmed, Probable and Suspected individuals in the data and contacts.

There are over 10,000 Suspected cases in the dataset, most from Sierra Leone and Liberia, which is reflected in the plots over time (Figure s, top row). The pattern in the proportion reporting funeral and non-funeral exposures (Figure s, bottom row) is very similar to that in the main text Fig 1.

The patterns in the nature of the exposure and the type of relationships reported change very little when we include the Suspected cases, Table r. The majority of reported exposure events still occur between family members (close and extended). As before, of those explicitly reporting the type of transmission, the majority of non-funeral exposures involve direct physical contact or contact with bodily fluids and a large proportion of funeral exposures report touching the corpse.

In adding Suspected cases we have included a large subset of cases who name contacts who are matched to Confirmed, Probable and Suspected cases. Therefore, the number of times individuals are reported as a funeral or a non-funeral contact is higher for the CPS subset than for the CP subset, Figure t, panels A&B. The trends in the proportion of family or HCW relationships reported by HCW and non-HCW are very similar to those of the CP dataset, and similarly the proportion of HCW cases over time does not vary substantially from the CP version (Figure t, panels C&D compared to Figure e). However, confidence intervals are slightly larger due to the loss of power.

In the main text we included a linear regression of R as a function of the proportion reporting funeral exposures. When we add Suspected cases to this analysis results are similar: reducing the proportion of individuals reporting funeral exposures to less than 23% (95% CI: 11, 35%) [CPS] and less than 29% (95% CI: 21, 38%) [CP] will result in R <1 (Figure t, panel E). An absolute reduction of 0.1 in the proportion reporting funeral exposures would lead to an absolute reduction of 0.10 (95%CI 0.04, 0.15) in R (p<0.001) [CPS] and 0.10 (95%CI 0.05, 0.15) in R (p<0.001) [CP].

Similarly we examined the relationship between R and the delay between symptoms onset and hospitalization (Figure t, panel F). When we add Suspected cases to this analysis results are similar: increasing the proportion of individuals hospitalized ≤ 4 days from symptom onset to more than 65% (95% CI 60%, 69%) [CPS] and more than 64% (95%CI: 59, 68%) [CP] will result in R <1. An absolute increase of 10% in proportion of individuals hospitalized ≤ 4 days from symptom onset would lead to an absolute reduction of 0.20 (95%CI: 0.12, 0.28, p<0.001) in R [CPS] and 0.19 (95%CI: 0.12, 0.25, p<0.001) in R [CP].

The observed cumulative distributions of the delay between onset, death and hospitalization and the exposure event are very similar with and without the Suspected cases included in the matched pairs, Figure u.

Table s: Number of Confirmed, Probable and Suspected (CPS) cases naming 0, 1, 2, or 3 non-funeral exposures and 0, 1, or 2 funeral exposures. (Equivalent of Table a)

|  | | **Number of funeral exposures reported** | | | | | | | | | | | |
| --- | --- | --- | --- | --- | --- | --- | --- | --- | --- | --- | --- | --- | --- |
|  |  | **All** | | | **Guinea** | | | **Liberia** | | | **Sierra Leone** | | |
|  |  | **0** | **1** | **2** | **0** | **1** | **2** | **0** | **1** | **2** | **0** | **1** | **2** |
| **Number of non-funeral exposures reported** | **0** | 22159 | 343 | 12 | 2649 | 40 | 1 | 5681 | 70 | 3 | 13829 | 233 | 8 |
|  | **1** | 4112 | 1950 | 55 | 452 | 231 | 0 | 1716 | 292 | 16 | 1944 | 1427 | 39 |
|  | **2** | 703 | 285 | 84 | 106 | 44 | 2 | 221 | 30 | 14 | 376 | 211 | 68 |
|  | **3** | 118 | 26 | 25 | 14 | 3 | 1 | 76 | 7 | 4 | 28 | 16 | 20 |


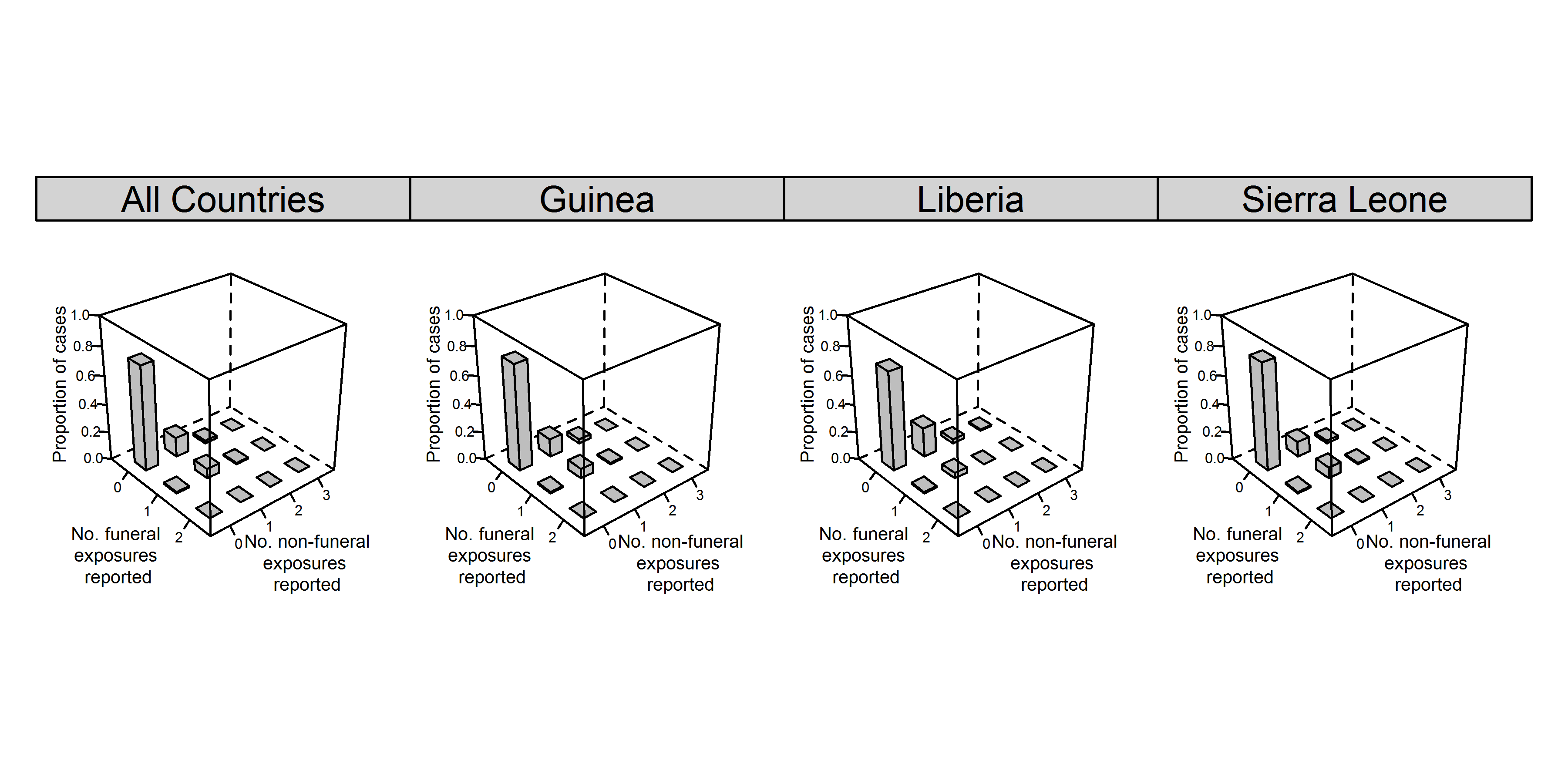


Figure r: Proportion of CPS cases naming 0, 1, 2, or 3 non-funeral exposures and 0, 1, or 2 funeral exposures (equivalent of Figure S3).


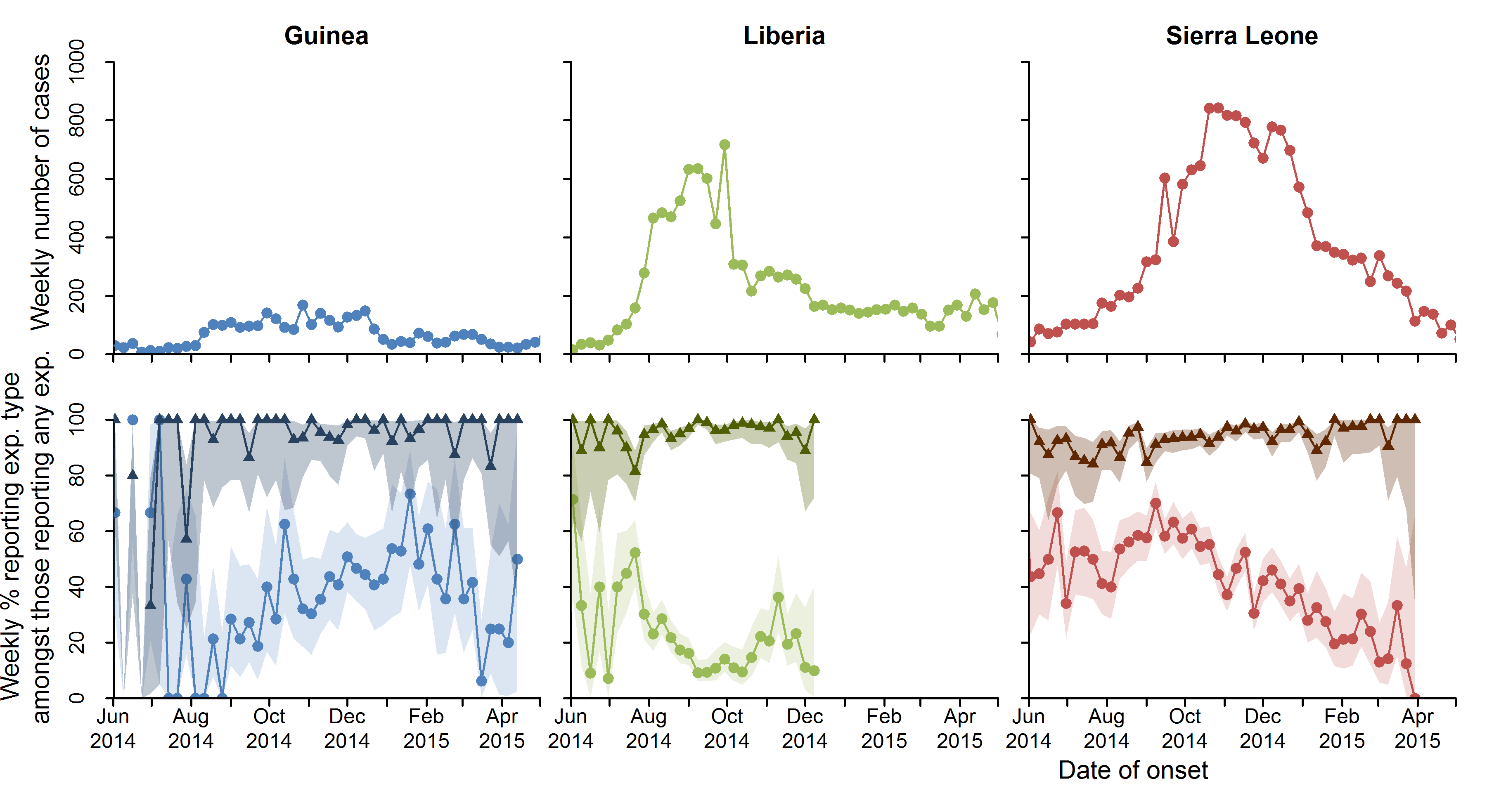


**Figure s: Total number of Confirmed, Probable and Suspected (CPS) cases over time (top row), percentage of cases who report an exposure that is a non-funeral exposure (second row, dark triangles) and a funeral exposure (second row, light circles).**


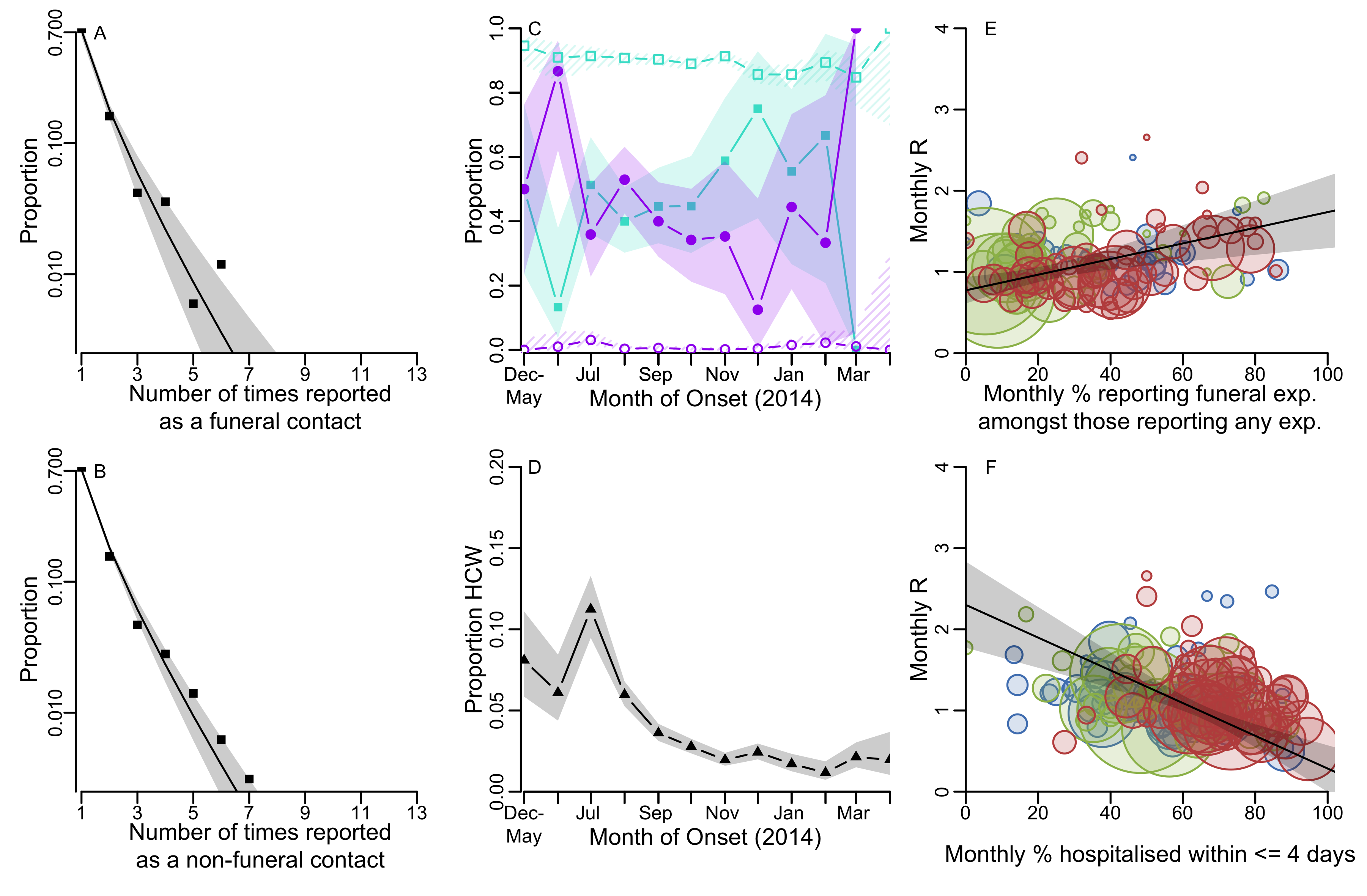


Figure t: A: the out-degree distribution of the network for funeral exposures, shows the probability that a named contact is named as a funeral exposure by a certain number of cases. Maximum likelihood logarithmic distribution is shown. B: as A, but non-funeral. C: Proportion of non-funeral exposures reported by HCWs (solid shading) and non-HCWs (hashed shading) which are with close or extended family or friends (turquoise) and with patients and HCWs (purple), by month. (Note that for HCW we have assumed that exposures reported with colleagues are with a HCW.) D: Proportion of cases who are health care workers (HCWs) in the WHO line-list, by month of symptom onset. E: Scatter plot of monthly proportion of incident cases who reported a funeral exposure among those reporting any exposure and monthly estimated reproduction numbers R from the supplemented incidence data *[*[*1*](#_ENREF_1)*]* (method as previously *[*[*3*](#_ENREF_3)*]*). Each point is a district month, colors are blue for Guinea, green for Liberia and red for Sierra Leone. We use a weighted regression method which takes account of the uncertainties in the data *[*[*6*](#_ENREF_6)*]*; trend lines are shown with 95% confidence intervals. The area of each circle is proportional to the weight of that point (see methods). The black trend line is for the whole dataset. F: Scatter plot of monthly proportion of incident cases who were hospitalized within <=4 days of symptom onset among with a reported hospitalization date and R from the supplemented incidence data *[*[*1*](#_ENREF_1)*]* (method and colors as A.).


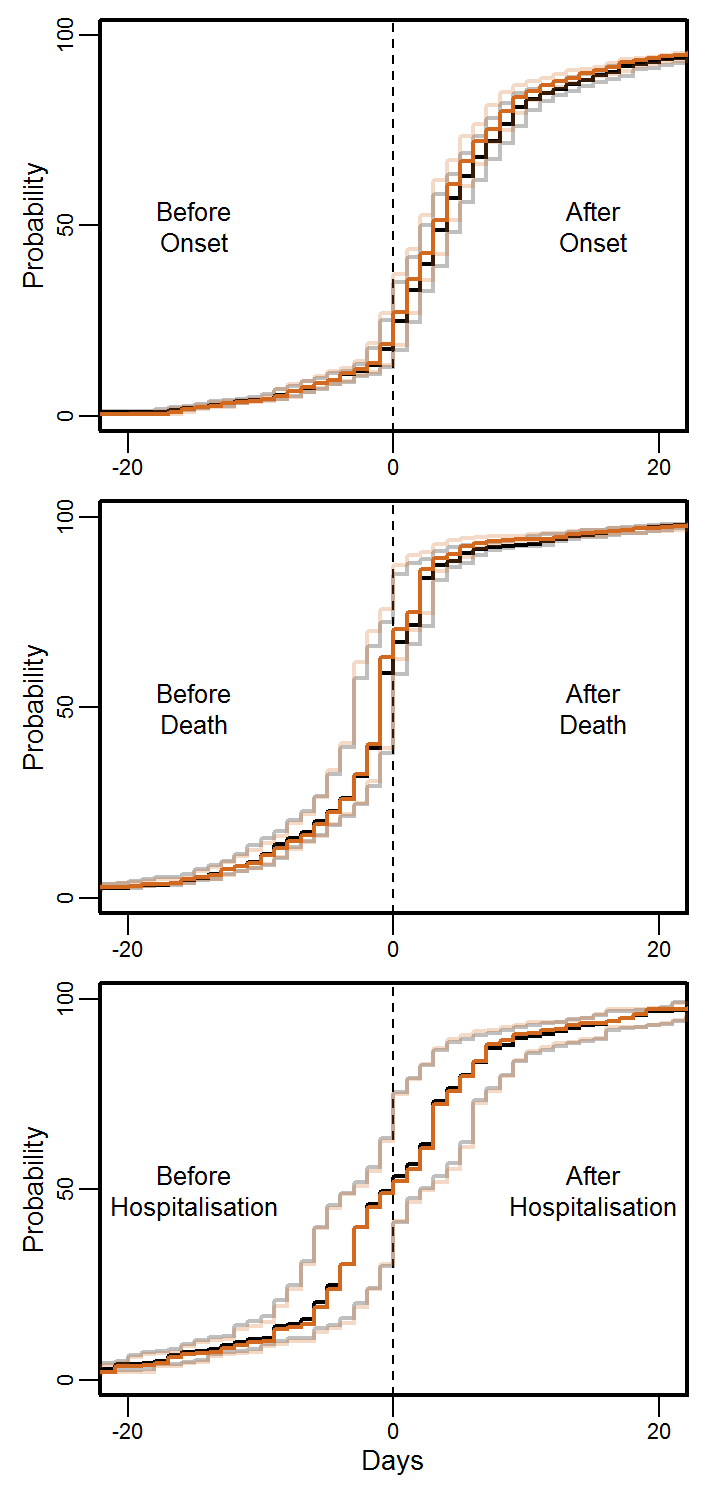


Figure u: The observed cumulative distribution functions for the midpoint (black line for CPS, orange forCP) and start and end (lighter colored lines) of the reported non-funeral exposure relative to onset (top), death (middle) and hospitalization (bottom) of the contact.

# References

1. W. H. O. Ebola Response Team (2015) West African Ebola Epidemic after One Year - Slowing but Not Yet under Control. N Engl J Med 372: 584-587.

2. World Health Organization (2014) Case definition recommendations for Ebola or Marburg virus diseases <http://www.who.int/csr/resources/publications/ebola/ebola-case-definition-contact-en.pdf>.

3. W. H. O. Ebola Response Team (2014) Ebola virus disease in West Africa--the first 9 months of the epidemic and forward projections. N Engl J Med 371: 1481-1495.

4. Fraser C (2007) Estimating individual and household reproduction numbers in an emerging epidemic. PLoS One 2: e758.

5. Cori A, Ferguson NM, Fraser C, Cauchemez S (2013) A new framework and software to estimate time-varying reproduction numbers during epidemics. Am J Epidemiol 178: 1505-1512.

6. Ripley BD, Thompson M (1987) Regression techniques for the detection of analytical bias. Analyst 112: 377-383.

7. Therneau T (2014) deming: Deming, Thiel-Sen and Passing-Bablock Regression. R package version 1.0-1. <http://CRAN.R-project.org/package=deming>

8. R Core Team (2014) R Foundation for Statistical Computing, Vienna, Austria.

9. Kullback S, Leibler RA (1951) On information and sufficiency. Annals of Mathematical Statistics 22: 79-86.

10. Burnham KP, Anderson DR (2002) Model selection and multimodel inference: a practical information-theoretic approach: Springer-Verlag.

11. Lloyd-Smith JO, Schreiber SJ, Kopp PE, Getz WM (2005) Superspreading and the effect of individual variation on disease emergence. Nature 438: 355-359.

12. Stumpf MPH, Wiuf C, May RM (2005) Subnets of scale-free networks are not scale-free: Sampling properties of networks. PNAS 102: 4221-4224
