# Supplementary material for: Exposure Patterns Driving Ebola Transmission in West Africa: A Retrospective Observational Study
Source: PLoS Med. 2016 Nov 15;13(11):e1002170. doi: 10.1371/journal.pmed.1002170 (PMC5112802; doi:10.1371/journal.pmed.1002170)
Supplement: S2 Text — (PDF) [file pmed.1002170.s003.pdf]

VIRAL HEMORHAGIC FEVER CASE INVESTIGATION FORM

Outbreak Case ID: ______

Health Facility Case ID: ______

Date of Case Report: ____/____/_____ (D, M, Yr)

Section 1. Patient Information

Patient's Surname: ______________________ Other Names:____________________________ Age: _______ ☐ Years ☐ Months

Gender: ☐ Male ☐ Female Phone Number of Patient/Family Member:_____________________ Owner of Phone: ________________

Status of Patient at Time of This Case Report: ☐ Alive ☐ Dead *If dead,* Date of Death: ___/___/____ (D, M, Yr)

Permanent Residence:

Head of Household: __________________________ Village/Town: _______________________ Parish: __________________________

Country of Residence: _________________ District: ____________________________ Sub-County: ____________________________

Occupation:

☐ Farmer ☐ Butcher ☐ Hunter/trader of game meat ☐ Miner ☐ Religious leader ☐ Housewife ☐ Pupil/student ☐ Child

☐ Businessman/woman; type of business: ___________________ ☐ Transporter; type of transport: ___________________________

☐ Healthcare worker; position: _________________ healthcare facility: ___________________ ☐ Traditional/spiritual healer

☐ Other; please specify occupation: ____

Location Where Patient Became Ill:

Village/Town: _________________________ District: _________________________ Sub-County: _________________________

GPS Coordinates at House: latitude: __________________ longitude: ________________________

If different from permanent residence, Dates residing at this location: ___/___/____ - ___/___/____ (D, M, Yr)

Section 2. Clinical Signs and Symptoms

Date of Initial Symptom Onset: ____/____/______ **(D, M, Yr)**

Please tick an answer for ALL symptoms indicating if they occurred during this illness between symptom onset and case detection:

Symptom	Response
Fever	☐ Yes ☐ No ☐ Unk
If yes, Temp: ____° C Source: ☐ Axillary ☐ Oral ☐ Rectal	
Vomiting/nausea	☐ Yes ☐ No ☐ Unk
Diarrhea	☐ Yes ☐ No ☐ Unk
Intense fatigue/general weakness	☐ Yes ☐ No ☐ Unk
Anorexia/loss of appetite	☐ Yes ☐ No ☐ Unk
Abdominal pain	☐ Yes ☐ No ☐ Unk
Chest pain	☐ Yes ☐ No ☐ Unk
Muscle pain	☐ Yes ☐ No ☐ Unk
Joint pain	☐ Yes ☐ No ☐ Unk
Headache	☐ Yes ☐ No ☐ Unk
Cough	☐ Yes ☐ No ☐ Unk
Difficulty breathing	☐ Yes ☐ No ☐ Unk
Difficulty swallowing	☐ Yes ☐ No ☐ Unk
Sore throat	☐ Yes ☐ No ☐ Unk
Jaundice (yellow eyes/gums/skin)	☐ Yes ☐ No ☐ Unk
Conjunctivitis (red eyes)	☐ Yes ☐ No ☐ Unk
Skin rash	☐ Yes ☐ No ☐ Unk
Hiccups	☐ Yes ☐ No ☐ Unk
Pain behind eyes/sensitive to light	☐ Yes ☐ No ☐ Unk
Coma/unconscious	☐ Yes ☐ No ☐ Unk
Confused or disoriented	☐ Yes ☐ No ☐ Unk

Symptom	Response
**Unexplained bleeding from any site**	☐ Yes ☐ No ☐ Unk
If Yes:	
Bleeding of the gums	☐ Yes ☐ No ☐ Unk
Bleeding from injection site	☐ Yes ☐ No ☐ Unk
Nose bleed (epistaxis)	☐ Yes ☐ No ☐ Unk
Bloody or black stools (melena)	☐ Yes ☐ No ☐ Unk
Fresh/red blood in vomit (hematemesis)	☐ Yes ☐ No ☐ Unk
Digested blood/"coffee grounds" in vomit	☐ Yes ☐ No ☐ Unk
Coughing up blood (hemoptysis)	☐ Yes ☐ No ☐ Unk
Bleeding from vagina, other than menstruation	☐ Yes ☐ No ☐ Unk
Bruising of the skin (petechiae/ecchymosis)	☐ Yes ☐ No ☐ Unk
Blood in urine (hematuria)	☐ Yes ☐ No ☐ Unk
Other hemorrhagic symptoms	☐ Yes ☐ No ☐ Unk

If yes, please specify: ____________________________

Other non-hemorrhagic clinical symptoms: ☐ Yes ☐ No ☐ Unk

If yes, please specifiy: ____________________________

Section 3. Hospitalization Information

At the time of this case report, is the patient hospitalized or currently being admitted to the hospital? ☐ Yes ☐ No

If yes, Date of Hospital Admission: ____/____/_____ (D, M, Yr) Health Facility Name: ____

Village/Town: __________________________ District: _______________________ Sub-County: __________________________

Is the patient in isolation or currently being placed there? ☐ Yes ☐ No *If yes,* date of isolation: ____/____/_____ (D, M, Yr)

Was the patient hospitalized or did he/she visit a health clinic previously for this illness? ☐ Yes ☐ No ☐ Unk

If yes, please complete a line of information for each previous hospitalization:

Dates of Hospitalization	Health Facility Name	Village	District	Was the patient isolated?
___/___/____ - ___/___/____ (D, M, Yr)				☐ Yes ☐ No
___/___/____ - ___/___/____ (D, M, Yr)				☐ Yes ☐ No

Outbreak Case ID:

Section 4. Epidemiological Risk Factors and Exposures

IN THE PAST ONE(1) MONTH PRIOR TO SYMPTOM ONSET:

1. Did the patient have contact with a known or suspect case, or with any sick person before becoming ill? ☐ Yes ☐ No ☐ Unk

If yes, please complete one line of information for each sick source case:

Name of Source Case	Relation to Patient	Dates of Exposure (D, M, Yr)	Village	District	Was the person dead or alive ?	Contact Types**
		___/___/___ - ___/___/___			☐ Alive<br>☐ Dead, date of death: ___/___/____ (D, M, Y)	
		___/___/___ - ___/___/___			☐ Alive<br>☐ Dead, date of death: ___/___/____ (D, M, Y)	
		___/___/___ - ___/___/___			☐ Alive<br>☐ Dead, date of death: ___/___/____ (D, M, Y)	

****Contact Types:** **(list all that apply)**

- **1** – Touched the body fluids of the case (blood, vomit, saliva, urine, feces)
- **2** – Had direct physical contact with the body of the case (alive or dead)
- **3** – Touched or shared the linens, clothes, or dishes/eating utensils of the case
- **4** – Slept, ate, or spent time in the same household or room as the case

2. Did the patient attend a funeral before becoming ill? ☐ Yes ☐ No ☐ Unk

If yes, please complete one line of information for each funeral attended:

Name of Deceased Person	Relation to Patient	Dates of Funeral Attendance (D, M, Yr)	Village	District	Did the patient participate (carry or touch the body)?
		___/___/____ - ___/___/____			☐ Yes ☐ No
		___/___/____ - ___/___/____			☐ Yes ☐ No

3. Did the patient travel outside their home or village/town before becoming ill? ☐ Yes ☐ No ☐ Unk

If yes, Village: ____________________ District: ____________________ Date(s): ___/___/____ - ___/___/____ (D, M, Yr)

4. Was the patient hospitalized or did he/she go to a clinic or visit anyone in the hospital before this illness? ☐ Yes ☐ No ☐ Unk

If yes, Patient Visited: ____________________ Date(s): ___/___/____ - ___/___/____ (D, M, Yr)

Health Facility Name: ____________________ Village: ____________________ District: ____________________

5. Did the patient consult a traditional/spiritual healer before becoming ill? ☐ Yes ☐ No ☐ Unk

If yes, Name of Healer: ____________________Village: ______________ District: ______________ Date: ___/___/____ (D, M, Yr)

6. Did the patient have direct contact (hunt, touch, eat) with animals or uncooked meat before becoming ill? ☐ Yes ☐ No ☐ Unk

If yes, please tick all that apply:

**Animal:**	**Status (check one only):**
☐ Bats or bat feces/urine	☐ Healthy ☐ Sick/Dead
☐ Primates (monkeys)	☐ Healthy ☐ Sick/Dead
☐ Rodents or rodent feces/urine	☐ Healthy ☐ Sick/Dead
☐ Pigs	☐ Healthy ☐ Sick/Dead
☐ Chickens or wild birds	☐ Healthy ☐ Sick/Dead
☐ Cows, goats, or sheep	☐ Healthy ☐ Sick/Dead
☐ Other; *specify*______________	☐ Healthy ☐ Sick/Dead

7. Did the patient get bitten by a tick in the past 2 weeks? ☐ Yes ☐ No ☐ Unk

Section 5. Clinical Specimens and Laboratory Testing

Specimen/shipping instructions:

- Label sample with **patient name, date of collection,** and **case ID**
- Send sample **cold** with a **cold/ice pack,** and **packaged appropriately**.
- Collect whole blood in a purple top (EDTA) tube – green or red top tubes acceptable if purple not available
- **Preferred sample volume = 4ml** (minimum sample volume = 2ml)

Has this patient had a sample submitted previously? ☐ Yes ☐ No

Sample 1: *Do not complete UVRI Only*

Sample Collection Date: ____/____/______ (D, M, Yr)

Sample Type:

☐ Whole Blood
☐ Post-mortem heart blood
☐ Skin biopsy
☐ Other specimen type, specify: ______________

Sample 2: *Do not complete UVRI Only*

Sample Collection Date: ____/____/______ (D, M, Yr)

Sample Type:

☐ Whole Blood
☐ Post-mortem heart blood
☐ Skin biopsy
☐ Other specimen type, specify: ______________

Section 6. Case Report Form Completed by:

Name: ____________________ Phone: ____________________ E-mail: ____________________

Position: ____________________ District: ____________________ Health Facility: ____________________

Information provided by: ☐ Patient ☐ Proxy; *If proxy*, Name:____________________ Relation to Patient: ______________

Case Name: ______________________ Outbreak Case ID: ______________________

****If the patient is deceased or has already recovered from illness, please fill out the next section.***
****If the patient is currently admitted to the hospital, leave the next section blank (it will be completed upon discharge)***

Section 7. Patient Outcome Information

Please fill out this section at the time of patient recovery and discharge from the hospital OR at the time of patient death.

Date Outcome Information Completed: ____/____/_____ (D, M, Yr)

Final Status of the Patient: ☐ Alive ☐ Dead

Did the patient have signs of unexplained bleeding at any time during their illness? ☐ Yes ☐ No ☐ Unk

If yes, please specify: ____

If the patient has recovered and been discharged from the hospital:

Name of hospital discharged from: ______________________ District: ______________________

If the patient was isolated, Date of discharge from the isolation ward: ____/____/______ (D, M, Yr)

Date of discharge from the hospital: ____/____/______ (D, M, Yr)

If the patient is dead:

Date of Death: ____/____/______ (D, M, Yr)

Place of Death: ☐ Community ☐ Hospital: ______________ ☐ Other: ______________

Village: ______________ District: ______________ Sub-County: ______________

Date of Funeral/Burial: ____/____/______ (D, M, Yr) Funeral conducted by: ☐ Family/community ☐ Outbreak burial team

Place of Funeral/Burial:

Village: ______________ District: ______________ Sub-County: ______________

Please tick an answer for ALL symptoms indicating if they occurred at any time during this illness including during hospitalization:

Fever	☐ Yes ☐ No ☐ Unk
If yes, Temp: ____º C Source: ☐ Axillary ☐ Oral ☐ Rectal	
Vomiting/nausea	☐ Yes ☐ No ☐ Unk
Diarrhea	☐ Yes ☐ No ☐ Unk
Intense fatigue/general weakness	☐ Yes ☐ No ☐ Unk
Anorexia/loss of appetite	☐ Yes ☐ No ☐ Unk
Abdominal pain	☐ Yes ☐ No ☐ Unk
Chest pain	☐ Yes ☐ No ☐ Unk
Muscle pain	☐ Yes ☐ No ☐ Unk
Joint pain	☐ Yes ☐ No ☐ Unk
Headache	☐ Yes ☐ No ☐ Unk
Cough	☐ Yes ☐ No ☐ Unk
Difficulty breathing	☐ Yes ☐ No ☐ Unk
Difficulty swallowing	☐ Yes ☐ No ☐ Unk
Sore throat	☐ Yes ☐ No ☐ Unk
Jaundice (yellow eyes/gums/skin)	☐ Yes ☐ No ☐ Unk
Conjunctivitis (red eyes)	☐ Yes ☐ No ☐ Unk
Skin rash	☐ Yes ☐ No ☐ Unk
Hiccups	☐ Yes ☐ No ☐ Unk
Pain behind eyes/sensitive to light	☐ Yes ☐ No ☐ Unk
Coma/unconscious	☐ Yes ☐ No ☐ Unk
Confused or disoriented	☐ Yes ☐ No ☐ Unk

Other non-hemorrhagic clinical symptoms: ☐ Yes ☐ No ☐ Unk

If yes, please specifiy: ______________________
