# Supplementary material for: Exposure Patterns Driving Ebola Transmission in West Africa: A Retrospective Observational Study
Source: PLoS Med. 2016 Nov 15;13(11):e1002170. doi: 10.1371/journal.pmed.1002170 (PMC5112802; doi:10.1371/journal.pmed.1002170)
Supplement: S3 Text — (PDF) [file pmed.1002170.s004.pdf]

FICHE DE NOTIFICATION DE FIEVRE HEMORRAGIQUE VIRALE

Numéro d'identification du patient: ____________

Autre numéro d'identification : ____________

Date de Notification: ____/____/_____ (J, M, A)

Section 1. Information sur le Patient

Nom de Famille: ________________________ Autres Noms:_______________________________ Age: _______ ☐ Années ☐ Mois

Sexe: ☐ Masculin ☐ Féminin Numéro de Téléphone (Patient/Famille):_________________ Propriétaire du Téléphone: ______________

Etat du patient au moment de la collecte d'information: ☐ Vivant ☐ Décédé *Si décédé*, Date du Décès: ___/___/____ (J, M, A)

Lieu de Résidence Permanente:

Nom du Chef de Famille: __________________________ Village/Ville: ______________________ Préfecture: _____________________

Pays de Résidence: _____________________ Région: __________________________ Sous-Préfecture: __________________________

Occupation:

☐ Planteur/Eleveur ☐ Boucher ☐ Chasseur/Vendeur de viande de brousse ☐ Mineur ☐ Chef religieux

☐ Ménagère ☐ Etudiant ☐ Enfant ☐ Médecin traditionnel

☐ Commerçant; type de commerce: _______________________ ☐ Transporteur; type de transport: ___________________________

☐ Personnel de santé; position: ________________________ nom du centre médical: ______________________________

☐ Autre; précisez le métier: ____

Endroit où le Patient est Tombé Malade:

Village/Ville: __________________________ Préfecture: _________________________ Sous- Préfecture: ________________________

Coordonnées GPS de la maison: Latitude: ________________________ Longitude: ________________________

Si ce n'est pas la résidence permanente, Dates de résidence dans cet endroit: ___/___/____ - ___/___/____ (J, M, A)

Section 2. Signes Cliniques et Symptômes

Date de début des signes et symptômes: ____/____/______ (J, M, A)

Cochez tous les signes et symptômes observés ou ressentis entre la date de début de la maladie et la date de notification:

Fièvre ☐ Oui ☐ Non ☐ Inc

Si oui, Temp: ____° C Mesure: ☐ Creux Axillaire ☐ Bouche ☐ Rectale

Nausées / Vomissements ☐ Oui ☐ Non ☐ Inc

Diarrhées ☐ Oui ☐ Non ☐ Inc

Fatigue générale intense ☐ Oui ☐ Non ☐ Inc

Perte d'appétit / anorexie ☐ Oui ☐ Non ☐ Inc

Douleurs abdominales ☐ Oui ☐ Non ☐ Inc

Douleurs thoraciques ☐ Oui ☐ Non ☐ Inc

Douleurs musculaires ☐ Oui ☐ Non ☐ Inc

Douleurs articulaires ☐ Oui ☐ Non ☐ Inc

Céphalées ☐ Oui ☐ Non ☐ Inc

Toux ☐ Oui ☐ Non ☐ Inc

Difficultés à respirer ☐ Oui ☐ Non ☐ Inc

Difficultés à avaler ☐ Oui ☐ Non ☐ Inc

Mal à la gorge ☐ Oui ☐ Non ☐ Inc

Ictère (conjonctives/gencives/peau) ☐ Oui ☐ Non ☐ Inc

Conjonctivite (œil rouge) ☐ Oui ☐ Non ☐ Inc

Eruptions cutanées ☐ Oui ☐ Non ☐ Inc

Hoquet ☐ Oui ☐ Non ☐ Inc

Douleurs retro-orbitaires/photophobie ☐ Oui ☐ Non ☐ Inc

Coma / perte de conscience ☐ Oui ☐ Non ☐ Inc

Confusion ou désorientation ☐ Oui ☐ Non ☐ Inc

Saignements ☐ Oui ☐ Non ☐ Inc

Si Oui:

Saignements des gencives ☐ Oui ☐ Non ☐ Inc

Saignements aux sites d'injections ☐ Oui ☐ Non ☐ Inc

Saignements du nez (épistaxis) ☐ Oui ☐ Non ☐ Inc

Selles rouges ou noires (mélénas) ☐ Oui ☐ Non ☐ Inc

Vomissements sanglants (hématémèses) ☐ Oui ☐ Non ☐ Inc

Vomissement noirâtre (vomito negro) ☐ Oui ☐ Non ☐ Inc

Toux sanglante (hémoptysie) ☐ Oui ☐ Non ☐ Inc

Saignements vaginaux, en dehors des règles ☐ Oui ☐ Non ☐ Inc

Hématomes / Pétéchies / purpura ☐ Oui ☐ Non ☐ Inc

Sang dans les urines (hématurie) ☐ Oui ☐ Non ☐ Inc

Autres signes hémorragiques ☐ Oui ☐ Non ☐ Inc

Si oui, précisez: ___________________________

Autres signes cliniques non-hémorragiques: ☐ Oui ☐ Non ☐ Inc

Si oui, précisez: ___________________________

Section 3. Informations sur l'hospitalisation

Au moment de cette notification, le malade est-il déjà hospitalisé ou en cours d'admission à l'hôpital? ☐ Oui ☐ Non

Si oui, Date d'hospitalisation: ____/____/_____ (J, M, A) Nom de l'hôpital: ____

Village/Ville: ________________________ Préfecture: _______________________ Sous-Préfecture: ________________________

Le malade est-il en isolement/en cours d'isolement? ☐ Oui ☐ Non *Si oui, D*ate d'isolement: ____/____/_____ (J, M, A)

Le malade était-il hospitalisé ailleurs ou a visité un centre de soins pour la maladie actuelle? ☐ Oui ☐ Non ☐ Inc

Si oui, veuillez compléter une ligne ci-dessous pour chacune des hospitalisations précédentes:

Dates d'hospitalisation	Nom du centre médical	Village	Préfecture	Le patient était-il en isolement?
___/___/____ - ___/___/____ (J, M, A)				☐ Oui<br>☐ Non
___/___/____ - ___/___/____ (J, M, A)				☐ Oui<br>☐ Non

Numéro d'identification du patient:

Section 4. Epidémiologie / Facteurs d'expositions

PENDANT LE MOIS PRÉCÉDENT LE DÉBUT DES SYMPTÔMES:

1. Il y a-t-il eu contacts avec un malade Ebola, connu/suspect, ou simplement avec une personne malade? ☐ Oui ☐ Non ☐ Inc

Si oui, veuillez compléter une ligne ci-dessous pour chacun des malades pouvant être une source de contamination:

Nom du malade potentiel	Lien de parenté	Date(s) du contact (J, M, A)	Village	Préfecture	Est-ce-que la personne était vivante ou décédée ?	Types de contact**
		___/___/___ - ___/___/___			☐ Vivante<br>☐ Décédée, Date: ___/___/____ (J, M, A)	
		___/___/___ - ___/___/___			☐ Vivante<br>☐ Décédée, Date: ___/___/____ (J, M, A)	
		___/___/___ - ___/___/___			☐ Vivante<br>☐ Décédée, Date: ___/___/____ (J, M, A)	

****Type de contact: (indiquez toutes les possibilités)**

- **1** – A touché des secrétions/excrétions du malade (sang, vomissures, salive, urine, selles)
- **2** – A touché directement le corps du malade (vivant ou décédé)
- **3** – A touché ou partagé linges, habits, plats/assiettes, instruments avec le malade
- **4** – A dormi ou mangé avec, ou séjourné dans la même maison ou pièce que le malade

2. Est-ce-que le patient a participé à des funérailles avant la maladie actuelle? ☐ Oui ☐ Non ☐ Inc

Si oui, veuillez compléter une ligne ci-dessous pour chacune des participations à un enterrement:

Nom de la personne décédée	Lien de parenté	Dates de participation aux funérailles (J, M, A)	Village	Préfecture	Avez-vous porté ou touché le corps?
		___/___/____ - ___/___/____			☐ Oui ☐ Non
		___/___/____ - ___/___/____			☐ Oui ☐ Non

3. Le patient a-t-il voyagé en dehors de chez lui ou de son village/ville avant la maladie actuelle? ☐ Oui ☐ Non ☐ Inc

Si oui, Village: ________________ Préfecture: ________________ Date(s): ___/___/____ - ___/___/____ (J, M, A)

4. Le patient a-t-il été hospitalisé, a-t-il consulté dans un hôpital ou visité quelqu'un hospitalisé avant la maladie actuelle? ☐ Oui ☐ Non ☐ Inc

Si oui, Nom du patient: ________________ Date(s): ___/___/____ - ___/___/____ (J, M, A)

Nom du Centre Médical: ________________ Village: ________________ Préfecture: ________________

5. Le patient a-t-il consulté un médecin traditionnel avant la maladie actuelle? ☐ Oui ☐ Non ☐ Inc

Si oui, Nom du Médecin: ________________Village: ________________ Préfecture: ________________ Date: ___/___/____ (J, M, A)

6. Le patient a-t-il eu un contact direct (chasse, touché, mangé) avec des animaux ou de la viande crue avant de tomber malade? ☐Oui ☐Non ☐Inc

Si oui, cochez les cases nécessaires:

Animal:	Status (check one only):
☐ Chauve-souris (ou excrétions de)	☐ En bonne santé ☐ Malade/Mort
☐ Singes	☐ En bonne santé ☐ Malade/Mort
☐ Rongeurs (ou excrétions de)	☐ En bonne santé ☐ Malade/Mort
☐ Cochons	☐ En bonne santé ☐ Malade/Mort
☐ Volaille ou oiseaux sauvages	☐ En bonne santé ☐ Malade/Mort
☐ Vaches, chèvres, ou moutons	☐ En bonne santé ☐ Malade/Mort
☐ Autres; *précisez* :____________	☐ En bonne santé ☐ Malade/Mort

7. Est-ce-que le patient a eu une piqûre de tique dans les 2 dernières semaines? ☐ Oui ☐ Non ☐ Inc

Section 5. Prélèvements Biologiques pour le Laboratoire

Mode de prélèvements et d'envoi :

- Identifier le tube: **nom**, **date de prélèvement** et le **numéro d'identification du malade**
- Envoyer les échantillons avec **réfrigération,** and **emballés correctement**.
- Prélever le sang complet dans un tube EDTA (bouchon violet) tube – si non disponible, bouchon vert (héparine) ou rouge (sans anticoagulant) sont acceptables
- **Volume demandé = 4ml** (volume minimum = 2ml)

Est-ce qu'un prélèvement a déjà été soumis pour ce malade? ☐ Oui ☐ Non

Prélèvement 1: *Ne pas remplir*

Date du prélèvement: ____/____/______ (J, M, A)

Type de prélèvement:

☐ Sang complet
☐ Ponction cardiaque (*post-mortem)*
☐ Biopsie de peau
☐ Autre prélèvement, précisez: ________________

Prélèvement 2: *Ne pas remplir*

Date du prélèvement: ____/____/______ (J, M, A)

Type de prélèvement:

☐ Sang complet
☐ Ponction cardiaque (*post-mortem)*
☐ Biopsie de peau
☐ Autre prélèvement, précisez: ________________

Section 6. Fiche de notification complétée par:

Nom: ________________ Téléphone: ________________ E-mail: ________________

Role: ________________ District: ________________ Centre médical: ________________

Informations fournies par ☐ Patient ☐ Représentant; *Si représentant*, Nom:________________ Lien de parenté: ________________

Nom du Patient:		Numéro Identification du Patient:	

*****Si le patient est décédé ou est déjà convalescent ou guéri, veuillez remplir la section suivante.***
*****Si le malade va être admis à l'hôpital, ne complétez pas la section suivante, (ce sera fait lors de la sortie)***

Section 7. Statut final du patient

Veuillez remplir cette section lorsque le patient est guéri et sort de l'hôpital ou lors de son décès.

Date à laquelle les informations sont rapportées: ____/____/_____ (J, M, A)

Statut final du patient: ☐ Vivant ☐ Décédé

Est-ce-que le patient a eu des signes hémorragiques inexpliqués pendant la durée de la maladie? ☐ Oui ☐ Non ☐ Inc

Si oui, veuillez préciser: ____

Si le malade est guéri et sort de l'hôpital:

Nom de l'hôpital: ______________________________ Préfecture: ________________________________

Si le malade était en isolement, date de sortie de la zone d'isolement: ____/____/______ (J, M, A)

Date de sortie de l'hôpital: ____/____/______ (J, M, A)

Si le malade est décédé:

Date du décès: ____/____/______ (J, M, A)

Lieu du décès: ☐ Domicile ☐ Hôpital: ______________________ ☐ Ailleurs: _______________________________

Village: ______________________ Préfecture: ________________________ Sous- Préfecture: ______________________

Date des funérailles: ____/____/______ (J, M, A) Funérailles organisées par: ☐ Famille/communauté ☐ Equipe d'enterrement

Lieu des funérailles/enterrement:

Village: ______________________ Préfecture: ________________________ Sous- Préfecture: ______________________

Veuillez cocher une réponse pour tous les signes et symptômes, indiquant s'ils ont été trouvés ou non pendant toute la durée de la maladie (en tenant compte également de la période d'hospitalisation):

Fièvre	☐ Oui ☐ Non ☐ Inc
Si oui, Temp: ____° C Mesure: ☐ Creux Axillaire ☐ Bouche ☐ Rectale	
Nausées / Vomissements	☐ Oui ☐ Non ☐ Inc
Diarrhées	☐ Oui ☐ Non ☐ Inc
Fatigue générale intense	☐ Oui ☐ Non ☐ Inc
Perte d'appétit / Anorexie	☐ Oui ☐ Non ☐ Inc
Douleurs abdominales	☐ Oui ☐ Non ☐ Inc
Douleurs thoraciques	☐ Oui ☐ Non ☐ Inc
Douleurs musculaires	☐ Oui ☐ Non ☐ Inc
Douleurs articulaires	☐ Oui ☐ Non ☐ Inc
Céphalées	☐ Oui ☐ Non ☐ Inc
Toux	☐ Oui ☐ Non ☐ Inc
Difficultés à respirer	☐ Oui ☐ Non ☐ Inc
Difficultés à avaler	☐ Oui ☐ Non ☐ Inc
Mal à la gorge	☐ Oui ☐ Non ☐ Inc
Ictère (conjonctives/gencives/peau)	☐ Oui ☐ Non ☐ Inc
Conjonctivite (œil rouge)	☐ Oui ☐ Non ☐ Inc
Eruptions cutanées	☐ Oui ☐ Non ☐ Inc
Hoquet	☐ Oui ☐ Non ☐ Inc
Douleurs retro-orbitaires/photophobie	☐ Oui ☐ Non ☐ Inc
Coma / perte de conscience	☐ Oui ☐ Non ☐ Inc
Confusion ou désorientation	☐ Oui ☐ Non ☐ Inc

Autres signes ou symptômes cliniques non hémorragiques: ☐ Oui ☐ Non ☐ Inc

Si oui, Veuillez précisez: ____________________________
