# Supplementary material for: Exposure Patterns Driving Ebola Transmission in West Africa: A Retrospective Observational Study
Source: PLoS Med. 2016 Nov 15;13(11):e1002170. doi: 10.1371/journal.pmed.1002170 (PMC5112802; doi:10.1371/journal.pmed.1002170)
Supplement: S4 Text — (PDF) [file pmed.1002170.s005.pdf]

EBOLA CASE INVESTIGATION FORM – Sierra Leone

Date of Case Report: D D / M M / Y Y Y Y

Outbreak Case ID: KAI0507234

☐ Patient is a followed contact: ***Convert to CASE in VHF***

Complete at end of interview: ☐ suspect ☐ probable ☐ unk

Patient's Last Name: ____________ **First Name:** ____________

Age: ______ Unit: ☐ Years ☐ Months Gender: ☐ Male ☐ Female

Patient Status at Time of This Report: ☐ Alive ☐ Dead ***If dead,*** Date of Death:

Permanent Residence:

Head of Household: ____________ Village/Town: ____________

District: ____________ Chiefdom: ____________ Mobile phone #: ____________

Patient's Occupation:

☐ Healthcare worker (includes anyone involved with the patient: nurse, ambulance driver, hospital cleaner, etc.)

Position: ____________ Healthcare facility: ____________.

☐ Other; please specify occupation: ____________.

Location Where Patient Became Ill:

Village/Town: ____________ District: ____________ Chiefdom: ____________

Date Patient First Became Sick: D D / M M / Y Y Y Y

Read each one aloud and mark an answer for every symptom occurred during this illness (not only right now):

Fever	☐ Yes	☐ No	☐ Unk	Headache	☐ Yes	☐ No	☐ Unk
Vomiting/nausea	☐ Yes	☐ No	☐ Unk	Difficulty breathing	☐ Yes	☐ No	☐ Unk
Diarrhea	☐ Yes	☐ No	☐ Unk	Difficulty swallowing	☐ Yes	☐ No	☐ Unk
Conjunctivitis (red eyes)	☐ Yes	☐ No	☐ Unk	Skin rash	☐ Yes	☐ No	☐ Unk
Intense fatigue/weakness	☐ Yes	☐ No	☐ Unk	Hiccups	☐ Yes	☐ No	☐ Unk
Anorexia/loss of appetite	☐ Yes	☐ No	☐ Unk	Unexplained bleeding	☐ Yes	☐ No	☐ Unk
Abdominal pain	☐ Yes	☐ No	☐ Unk	If yes, please specify: ____________			
Muscle pain	☐ Yes	☐ No	☐ Unk	Other symptoms:	☐ Yes	☐ No	☐ Unk
Joint pain	☐ Yes	☐ No	☐ Unk	If yes, please specify: ____________			

At the time of this report, is the patient hospitalized or being admitted to the hospital? ☐ Yes ☐ No ☐ Unk

If yes, Date of Hospital Admission D D / M M / Y Y Y Y

Hospital Name: ____________ District: ____________

Is the patient now, or will he/she soon be, in an Ebola treatment unit? ☐ Yes ☐ No ☐ Unk

If yes, date of admission (or future admission to the ETU (isolation): D D / M M / Y Y Y Y

Was the patient hospitalized or visited a clinic previously for this illness (this includes any type of care: pharmacist, traditional healer, etc.)? ☐ Yes ☐ No ☐ Unk

If yes, Dates of Hospitalization: D D / M M / Y Y Y Y

Hospital Name: ____________ District: ____________

Uniprint-F 8A

KAI0507234 KAI0507234 KAI0507234

KAI0507234 KAI0507234 KAI0507234

IN THE PAST ONE (1) MONTH PRIOR TO SYMPTOM ONSET:

Outbreak Case ID: KAI0507234

1. Did the patient have contact with a suspected or confirmed Ebola case in the one month before becoming ill?

☐ Yes ☐ No ☐ Unk

If yes, please complete one line of information for each sick source case:

Name of Source Case	Relation to Patient	Date of Last Contact (DD, MM, YYYY)	Village/Town	District	Was the person dead or alive?
		/ /			☐ Alive<br>☐ Dead<br>Date of Death: / / (DD,MM,YYYY)
		/ /			☐ Alive<br>☐ Dead<br>Date of Death: / / (DD,MM,YYYY)

2. Did the patient attend a funeral in the one month before becoming ill? ☐ Yes ☐ No ☐ Unk

If yes, Name of deceased person	Relation to Patient	Date of Funeral (DD, MM, YYYY)	Village/Town	District	Did the patient participate? (carry or touch the body)?
		/ /			☐ Yes<br>☐ No

3. Did the patient travel outside their hometown or village/town before becoming ill? ☐ Yes ☐ No ☐ Unk

If yes, Village: ______ Chiefdom: ______

District: ______ Date(s): __/__/____ - __/__/____

Case Report Form Completed by:

Name: ______ Phone: ______ E-mail: ______

Position: ______ District: ______ Health Facility: ______

Information provided by:

☐ Patient ☐ Proxy *If proxy*, Name: ______ Relation to patient: ______

Patient Outcome Information:

Please fill out this section at the time of patient recovery and discharge from the hospital OR patient death.

Date Outcome Information Completed: __/__/____

Final Status of the Patient: ☐ Alive/Recovered ☐ Dead

If the patient has recovered and been discharged from the hospital:

Hospital discharged from: ______ District: ______

Date of discharge from the hospital: __/__/____

If the patient is dead:

Date of Death: __/__/____

Place of Death: ☐ Community ☐ Hospital District: ______

Date of Funeral/Burial: __/__/____

Funeral conducted by: ☐Family/community ☐ Outbreak burial team

Place of Funeral/Burial: Village: ______ Chiefdom: ______ District: ______

Uniprint-F_BA

EBOLA Clinical Specimens and Laboratory Testing

Outbreak Case ID: KAI0507234

Sample 2

Date of Specimen Collection: DD / MM / YYYY

Patient's Last Name: ______________ **First Name:** ______________

Age: ______ Unit: ☐ Years ☐ Months Gender: ☐ Male ☐ Female

Permanent Residence:

Head of Household: ______________ Village/Town: ______________

District: ______________ Chiefdom: ______________ Mobile phone #: ______________

Date Patient First Became Sick: DD / MM / YYYY

Specimen/shipping instructions:
- Label sample with **patient name, date of collection,** and **case ID**
- Send sample cold with a **cold/ice pack**, and **packaged appropriately**
- Collect whole blood in a purple top (EDTA) tube green or red top tubes acceptable if purple not available
- **Preferred** sample volume = **4ml** (minimum sample volume = 2ml)

Uniprint-F_SA

Has this patient had a sample submitted previously? ☐ Yes ☐ No ☐ Unk

Sample Type: ☐ Whole blood ☐ Skin biopsy ☐ Post-mortem heart blood ☐ Other specimen type, specify: ______________

TEAR HERE ------ TEAR HERE ------ TEAR HERE ------ TEAR HERE ------ TEAR HERE ------ TEAR HERE ------ TEAR HERE

EBOLA Clinical Specimens and Laboratory Testing

Outbreak Case ID: KAI0507234

Sample 1

Date of Specimen Collection: DD / MM / YYYY

Patient's Last Name: ______________ **First Name:** ______________

Age: ______ Unit: ☐ Years ☐ Months Gender: ☐ Male ☐ Female

Permanent Residence:

Head of Household: ______________ Village/Town: ______________

District: ______________ Chiefdom: ______________ Mobile phone #: ______________

Date Patient First Became Sick: DD / MM / YYYY

Specimen/shipping instructions:
- Label sample with **patient name, date of collection,** and **case ID**
- Send sample cold with a **cold/ice pack**, and **packaged appropriately**
- Collect whole blood in a purple top (EDTA) tube green or red top tubes acceptable if purple not available
- **Preferred** sample volume = **4ml** (minimum sample volume = 2ml)

Uniprint-F_SA

Has this patient had a sample submitted previously? ☐ Yes ☐ No ☐ Unk

Sample Type: ☐ Whole blood ☐ Skin biopsy ☐ Post-mortem heart blood ☐ Other specimen type, specify: ______________
