# Supplementary material for: Exposure Patterns Driving Ebola Transmission in West Africa: A Retrospective Observational Study
Source: PLoS Med. 2016 Nov 15;13(11):e1002170. doi: 10.1371/journal.pmed.1002170 (PMC5112802; doi:10.1371/journal.pmed.1002170)
Supplement: S5 Text — (PDF) [file pmed.1002170.s006.pdf]

LIBERIA EBOLA CASE INVESTIGATION FORM

CASE ID _ _ _ _ _ - _ _ _ _ _
County ID   Patient ID

Facility ID: ____________

REQUIRED FIELDS

Date of report MM__ DD__ YY__ **County of report**____________
Village of report ____________________
Investigation initiated by ☐ Case Investigation Team ☐ ETU ☐ CCC ☐ Burial ☐ Other________
Patient's surname______________________ **Patient's other names**______________________
Age (yrs) ____ (0 if <1 y.o.) **Sex** ☐M ☐ F **Date patient first became sick** MM___ DD___ YY___
Healthcare worker / Works in health setting ☐ Y ☐ N ☐ Unk
If yes, Position ____________________ Healthcare facility______________________
Family/friend/immediate contact name ____________________ **Phone number** ______________
Religion ☐ Christian ☐ Muslim ☐ Atheist ☐ Traditionalist ☐ Other ☐ Unk
Where patient lives Village/Town________________________ Clan/Zone____________________
District____________________ County ________________ Country____________________
Where patient first became sick Village/Town__________________ Clan/Zone__________________
District____________________ County ________________ Country____________________

Ask the patient about the following symptoms if possible, or else ask a close relative or friend

Fever	☐ Yes	☐ No	☐ Unk	Joint pain	☐ Yes	☐ No	☐ Unk
Vomiting/nausea	☐ Yes	☐ No	☐ Unk	Headache	☐ Yes	☐ No	☐ Unk
Diarrhea	☐ Yes	☐ No	☐ Unk	Cough	☐ Yes	☐ No	☐ Unk
Intense fatigue/weakness	☐ Yes	☐ No	☐ Unk	Difficulty breathing	☐ Yes	☐ No	☐ Unk
Anorexia/loss of appetite	☐ Yes	☐ No	☐ Unk	Difficulty swallowing	☐ Yes	☐ No	☐ Unk
Abdominal pain	☐ Yes	☐ No	☐ Unk	Hiccups	☐ Yes	☐ No	☐ Unk
Chest pain	☐ Yes	☐ No	☐ Unk	Unexplained bleeding	☐ Yes	☐ No	☐ Unk
Muscle pain	☐ Yes	☐ No	☐ Unk	*If yes,* list areas of body ______________			

Has the patient previously visited a health care facility for this illness? ☐ Yes ☐ No ☐ Unk
If yes, dates in facility MM___ DD___ YY___ to MM___ DD___ YY___
Facility name ____________________________ County ________________
Was the case previously a contact? (i.e. followed by contact tracers) ☐ Yes ☐ No ☐ Unk
Did the patient contact an ill person in the last 21 days before becoming ill? ☐ Yes ☐ No ☐ Unk

Name of source case	Last contact date	County	Village/Town	Status	Date of Death
	/ /			☐ Alive ☐ Dead	/ /
	/ /			☐ Alive ☐ Dead	/ /

Did patient attend a funeral in the last 21 days before becoming ill? ☐ Yes ☐ No ☐ Unk
If yes, Name deceased ______________ Funeral date MM___ DD___ YY___ Village ______________
County ________________________ Did the patient touch or carry the body? ☐ Yes ☐ No ☐ Unk
Did patient travel outside their home town in the last 21 days before becoming ill? ☐ Yes ☐ No ☐ Unk
If yes, Country of travel ☐ Liberia ☐ Guinea ☐ Sierra Leone ☐ Other____________________
If Liberia, Village ______________County ______________ Dates MM___ DD___ YY___ to MM___ DD___ YY___

Patient current status
☐ Admitted to ETU ☐ Admitted to CCC *if yes* ETU/CCC name__________________ on MM__ DD__ YY__
☐ Awaiting transportation to ETU or CCC
☐ Refused to go to an ETU or CCC because ______________________________
☐ Dead *If dead,* **Date of death** MM___ DD___ YY___ ☐ Not applicable

Epidemiological case classification ☐ Suspected ☐ Probable ☐ Not a case ☐ Unknown

Other comments

Completed By ____________________ Phone number ________________ Position ____________________
RETURN COMPLETED FORM TO THE COUNTY HEALTH TEAM - Date received MM____ DD____ YY____

LIBERIA EBOLA CASE INVESTIGATION PACKAGE

CONTACT LISTING FORM

CASE ID _ _ _ _ _ - _ _ _ _ _
County ID **Patient ID**

Case Information

Surname	Other Names	Sex	County of Residence	Clan/Zone	Village/Town	Date Illness Onset	Date of Isolation	Date of Death

Epidemiological case classification ☐ **Suspected** ☐ **Probable**

****For all information on location, please list information on where the contact will be residing for the next month.**

Contact Information

Surname	Other Names	Sex (M/F)	Age (yrs)	Phone Number	Relation to Case	Date of last Contact with Case (mm/dd/yy)	Type of Exposure* **list all**	Head of Household	County	Zone/Clan	Village/ Community/ Block	Village/ Community Leader	Healthcare Worker (Y/N) if yes, list facility

*Types of Exposure (list number) 1 = Touched the body fluids of the case (blood, vomit, saliva, urine, feces); 2= Had direct physical contact with the body of the case (alive or dead); 3= Touched or shared the linens, clothes, or dishes/eating utensils of the case; 4 = Slept, ate, or spent time in the same household or room as the case; 5=no direct contact reported, but need to be followed

Total number of contacts ____ Contact Sheet Filled by Name ____________________________ Position ______________________ Phone ________________

RETURN THIS COMPLETED FORM TO THE COUNTY HEALTH TEAM - Date Received MM___ DD___ YY___

LIBERIA EBOLA CASE INVESTIGATION PACKAGE

PATIENT OUTCOME FORM

CASE ID_ _ _ _ _ - _ _ _ _ _
County ID **Patient ID**

FILL OUT THIS SECTION AND GIVE TO ETU/CCC

Patient's surname ______________________ **Patient's other names**__________________________

Age (yrs) ____ (0 if <1y.o.) **Sex** ☐ M ☐ F

Where patient lives Village/Town_____________________________ Clan/Zone_______________________

District__________________________ County _____________________ Country______________________

Family/Friend/Immediate contact name_____________________**Phone number**______________________

Investigator name___________________________ **Investigator phone number**______________________

Date outcome information completed MM___ DD___ YY___

Final status of patient ☐Discharged/Recovered ☐ Dead

☐Discharged/Never tested positive ☐Discharged/Triage (no testing)

If patient recovered and discharged

Hospital/Ebola Treatment Unit discharged from ____

County ____________________________________ Date of discharge MM___ DD___ YY___

If patient is dead

Date of death MM___ DD___ YY___

Place of death ☐ Community location ☐ETU ☐CCC ☐Hospital ☐Specify location name_______________

Date of burial MM___ DD___ YY___ Burial conducted by ☐Family/Community ☐Burial Team

Completed By ___________________ Phone number ___________________ Affiliation ☐ CIT ☐ Burial team ☐ ETU ☐ CCC

Form 11/25/2014

Keep this form at location where patient is isolated (e.g. hand to ambulance driver).

Fill out at time of patient's recovery and discharge OR at time of patient's death.

Once patient outcome known, return to County Health Team

LIBERIA EBOLA CASE INVESTIGATION PACKAGE

LABORATORY SAMPLE SUBMISSION FORM

CASE ID_ _ _ _ _ - _ _ _ _ _
County ID Patient ID

Complete this section when sample is collected

Patient's surname ______________________ **Patient's other names**_______________________
Age (yrs) ____ (0 if <1y.o.) **Sex** □ M □ F
Where patient lives Village/Town____________________________Clan/Zone________________________
District__________________________ County ___________________ Country_______________________
Sample submitted by _________________________(name) **from** ________________________(facility)
Affiliation □ Community case investigator/County □ ETU □ CCC □ Burial team □ Other__________
Sample type □ Whole blood □ Swab □ Postmortem heart blood □ Other _____________________
Reason for testing □ Diagnosis □ Readiness for discharge
Date collected MM___ DD___ YY___ **Date submitted** MM___ DD___ YY___
Send results to ____________________________(name) **from** ______________________________(facility)
Phone number ________________________ Email ____

To be completed by Laboratory **Laboratory** ________________________________

Date received MM___ DD___ YY___ **Sample ID#** _____________________________
Date tested MM___ DD___ YY___
Result Interpretation □ Positive □ Negative □ Indeterminate
Other Test Results
______________ □ Positive □ Negative □ Indeterminate
______________ □ Positive □ Negative □ Indeterminate

Form 11/27/2014

- -

LIBERIA EBOLA CASE INVESTIGATION PACKAGE

LABORATORY SAMPLE SUBMISSION FORM

CASE ID_ _ _ _ _ - _ _ _ _ _
County ID Patient ID

Complete this section when sample is collected

Patient's surname ______________________ **Patient's other names**_______________________
Age (yrs) ____ (0 if <1y.o.) **Sex** □ M □ F
Where patient lives Village/Town____________________________Clan/Zone________________________
District__________________________ County ___________________ Country_______________________
Sample submitted by _________________________(name) **from** ________________________(facility)
Affiliation □ Community case investigator/County □ ETU □ CCC □ Burial team □ Other__________
Sample type □ Whole blood □ Swab □ Postmortem heart blood □ Other _____________________
Reason for testing □ Diagnosis □ Readiness for discharge
Date collected MM___ DD___ YY___ **Date submitted** MM___ DD___ YY___
Send results to ____________________________(name) **from** ______________________________(facility)
Phone number ________________________ Email ____

To be completed by Laboratory **Laboratory** ________________________________

Date received MM___ DD___ YY___ **Sample ID#** _____________________________
Date tested MM___ DD___ YY___
Result Interpretation □ Positive □ Negative □ Indeterminate
Other Test Results
______________ □ Positive □ Negative □ Indeterminate
______________ □ Positive □ Negative □ Indeterminate

Form 11/27/2014

FORM TO BE SENT WITH SAMPLE TO LAB

LIBERIA EBOLA CASE INVESTIGATION PACKAGE

Instructions for Case Investigation Completion and Form Submission

A case investigation form should be completed for all possible Ebola cases. Reporting is essential for monitoring and controlling the outbreak. The information provided here together with laboratory testing are essential in classifying a person as a probable or confirmed case or whether Ebola can be excluded as the cause of illness.

Completing the Case Investigation Form

1. Assign a **unique Case ID number**, provided by the county health team, in the top right corner of **all forms** in the Case Investigation Package to allow linking of all forms for one case.

2. A family member or friend's phone number MUST BE collected for all suspect Ebola cases to enable follow-up with the patient's family.

3. When collecting information on the date a patient first became sick (date of illness onset) and symptoms, collect the information directly from the patient if possible, or otherwise from a family member or friend if there is someone who would know when the suspect person started to feel sick

   - Use the provided calendars as a reference to help determine a precise date when interviewing
   - If the interviews are unable to provide a specific date of onset, a reasonable estimate based on information learned can be used.

4. Where patient lives - ask the patient where they are currently living now

5. Ask the patient about each of the symptoms on the form and indicate Yes, No, or Unknown for each symptom on the list. If there is bleeding that is not caused by an accident (by trauma), check this box and list all body areas with bleeding.

6. A healthcare worker includes any individual who is involved with or works in a health care facility e.g. hygienist, cleaner, ambulance driver as well as a nurse or doctor

7. If the patient is in an ETU or CCC, list the name of the facility and the date when they arrived there. Indicate if they are going to be taken to one today or as soon as possible. If the patient refuses to leave or the family refuses to allow them to go, describe why (e.g., cannot leave family, fear of ETU, community resistance).

8. Every completed form should classify the person as either Suspect, Probable, or Not A Case

   Suspect Case: Any person with acute fever and three or more of the symptoms on this form, OR any person with acute fever and signs of hemorrhage, OR any unexplained death

   Probable Case: A suspect case who also had contact with a confirmed or probable case in the three weeks prior to becoming ill OR a person with acute fever who had contact with a confirmed or probable case in the three weeks prior to becoming ill

   Not A Case: A person who was investigated but does not meet any of these definitions

What to do with the Case Investigation Package

9. Fill out completely and submit the **Case Investigation Form** to the County Health Team immediately.

10. Fill out completely and submit the **Contact Listing Form** to the County Health Team immediately to ensure contact tracing teams are dispatched.

11. Fill out the Case ID and basic patient information on the **Patient Outcome Information Form**. This should be kept with the patient and completed when the person's final outcome is known.

12. Fill out the Case ID and basic patient information on the **Laboratory Sample Submission Form**. This should be kept with the patient until a sample is collected, at which point the form should accompany the sample to the lab with the Case ID included.

13. Once this information has been received by the County Health Team it can be entered into the HMIS surveillance system. Case information collected should be submitted to the HMIS system online at https://ebola.dhis2.org/

Any questions or requests for user registration should be directed to the county health team.

November - 2014

S	M	T	W	T	F	S
						1
2	3	4	5	6	7	8
9	10	11	12	13	14	15
16	17	18	19	20	21	22
23	24	25	26	27	28	29
30						

December - 2014

S	M	T	W	T	F	S
	1	2	3	4	5	6
7	8	9	10	11	12	13
14	15	16	17	18	19	20
21	22	23	24	25	26	27
28	29	30	31			

January - 2015

S	M	T	W	T	F	S
				1	2	3
4	5	6	7	8	9	10
11	12	13	14	15	16	17
18	19	20	21	22	23	24
25	26	27	28	29	30	31

February - 2015

S	M	T	W	T	F	S
1	2	3	4	5	6	7
8	9	10	11	12	13	14
15	16	17	18	19	20	21
22	23	24	25	26	27	28

March - 2015

S	M	T	W	T	F	S
1	2	3	4	5	6	7
8	9	10	11	12	13	14
15	16	17	18	19	20	21
22	23	24	25	26	27	28
29	30	31				

April - 2015

S	M	T	W	T	F	S
			1	2	3	4
5	6	7	8	9	10	11
12	13	14	15	16	17	18
19	20	21	22	23	24	25
26	27	28	29	30		
