# Supplementary material for: Exposure Patterns Driving Ebola Transmission in West Africa: A Retrospective Observational Study
Source: PLoS Med. 2016 Nov 15;13(11):e1002170. doi: 10.1371/journal.pmed.1002170 (PMC5112802; doi:10.1371/journal.pmed.1002170)
Supplement: S6 Text — (PDF) [file pmed.1002170.s007.pdf]

FICHE DE NOTIFICATION DE FIEVRE HEMORRAGIQUE VIRALE (VERSION COURTE)

Numéro d'identification du patient : ____________

Date de Notification: ____/____/_____ (J, M, A)

Section 1. Information sur le Patient

Nom de Famille : ______________________ **Autres Noms** : __________________________

Age: ______ ☐ Ans ☐ Mois **Sexe:** ☐ Masculin ☐ Féminin

Numéro de Téléphone (Patient/famille): __________________________

Etat du patient à la date de notification : ☐Vivant ☐Décédé *Si décédé,* Date du Décès: ___/___/___ (J, M, A)

Lieu de Résidence Permanente :
Nom du Chef de Famille : ____________________ Pays : __________________ Région : __________________
Préfecture : __________________ Sous-préfecture : ___________________ Village/Ville : __________________

Occupation :
☐ Personnel de santé; Position : ____________________ Nom du centre médical : ______________________
☐ Autre; précisez : ________________________

Endroit où le patient est tombé malade : Pays : __________________ Préfecture : _____________________
Sous-préfecture : __________________ Village/Ville : _________________

Section 2. Signes Cliniques et Symptômes

Date du début des signes et symptômes : ____/____/______ (J, M, A)

Veuillez marquer une réponse pour CHAQUE symptôme indiquant s'il est survenu pendant <u>cette maladie</u> :

Fièvre	☐ Oui	☐ Non	☐ Inc
Nausées / vomissements	☐ Oui	☐ Non	☐ Inc
Diarrhées	☐ Oui	☐ Non	☐ Inc
Fatigue générale intense	☐ Oui	☐ Non	☐ Inc
Perte d'appétit / anorexie	☐ Oui	☐ Non	☐ Inc
Douleurs abdominales	☐ Oui	☐ Non	☐ Inc
Douleurs thoraciques	☐ Oui	☐ Non	☐ Inc
Douleurs musculaires	☐ Oui	☐ Non	☐ Inc
Douleurs articulaires	☐ Oui	☐ Non	☐ Inc
Céphalées	☐ Oui	☐ Non	☐ Inc
Toux	☐ Oui	☐ Non	☐ Inc
Difficultés à respirer	☐ Oui	☐ Non	☐ Inc
Difficultés à avaler	☐ Oui	☐ Non	☐ Inc
Mal à la gorge	☐ Oui	☐ Non	☐ Inc
Conjonctivite (œil rouge)	☐ Oui	☐ Non	☐ Inc
Eruptions cutanées	☐ Oui	☐ Non	☐ Inc
Hoquet	☐ Oui	☐ Non	☐ Inc

Saignements inexpliqués ☐ Oui ☐ Non ☐ Inc
Si oui, précisez: _________________________

Autres signes cliniques non-hémorragiques :
☐ Oui ☐ Non ☐ Inc *Si oui*, précisez:_______________

Section 3. Informations sur l'Hospitalisation

Au moment de cette notification, le patient est-il hospitalisé ou en cours d'admission à l'hôpital? ☐Oui ☐Non

Si oui, Date d'hospitalisation : ____/____/_____ (J, M, A) Nom du centre médical : _______________________
Préfecture : ________________________ Village/Ville : _______________________
Le patient est-il dans un CTE (isolement) or en cours d'isolement? ☐ Oui ☐ Non
Si oui, date d'isolement/admission au CTE : ____/____/_____ (J, M, A)

Le patient était-il hospitalisé ailleurs ou a-t-il visité un autre centre médical <u>pour cette maladie</u>?
☐ Oui ☐ Non ☐ Inc

Si oui, Dates d'hospitalisation : ___/___/____ - ___/___/____ (J, M, A)
Nom du centre médical : ________________ Préfecture : ________________ Village/Ville : ______________

Section 4. Epidémiologie / Facteurs d'Expositions

PENDANT LE MOIS PRÉCÉDENT LE DÉBUT DES SYMPTÔMES:

1. Le patient a-t-il eu des contacts avec un malade Ebola ou avec une personne malade? ☐Oui ☐Non ☐Inc

Si oui, veuillez compléter une ligne pour chacun des malades qui pouvant être une source de contamination :

Nom du cas source	Lien de parenté	Date de dernier contact (J, M, A)	Préfecture	Village/Ville	Est-ce que le malade était vivant ou décédé ?
		___/___/___			☐ Vivant<br>☐ Décédé, date du décès : __/__/__ (J, M, A)
		___/___/___			☐ Vivant<br>☐ Décédé, date du décès : __/__/__ (J, M, A)

2. Le patient a-t-il participé à des funérailles avant de tomber malade ? ☐ Oui ☐ Non ☐ Inc

Si oui, Nom du Décès : ____________________ Lien de parenté: ______________________________

Date des funérailles : ___/___/____ (J, M, A) Préfecture : _______________ Village/Ville : _____________

Le patient a-t-il porté ou touché le corps du décès ? ☐ Oui ☐ Non

3. Le patient a-t-il voyagé en dehors de son village/ville avant de tomber malade ? ☐ Oui ☐ Non ☐ Inc

Si oui, Préfecture : _______________ Village/Ville : ______________ Date(s) : ___/___/___ - ___/___/___ (J, M, A)

Section 5. Prélèvements Biologiques pour le Laboratoire

Est-ce qu'un prélèvement a déjà été soumis pour ce malade ? ☐ Oui ☐ Non

Ce prélèvement soumis par : Nom : ________________________ Centre médical : _____________________

Téléphone : ______________________ E-mail : _______________________

Prélèvement 1 : ☐

Date du prélèvement : ____/____/______ (J, M, A)

Type de prélèvement :

- ☐ Sang complet
- ☐ Ponction cardiaque (*post-mortem*)
- ☐ Biopsie de peau
- ☐ Swab orale
- ☐ Autre prélèvement, précisez: ____________

Prélèvement 2 : ☐

Date du prélèvement : ____/____/______ (J, M, A)

Type de prélèvement :

- ☐ Sang complet
- ☐ Ponction cardiaque (*post-mortem*)
- ☐ Biopsie de peau
- ☐ Swab orale
- ☐ Autre prélèvement, précisez: ____________

Section 6. Fiche de Notification Complétée par :

Nom : _________________________ Téléphone : _____________________ E-mail : _________________________

Section 7. Statut Final du Patient

Veuillez remplir cette section lorsque le patient est sorti guéri de l'hôpital <u>OU</u> lorsqu'il est décédé

Date à laquelle les informations du statut final sont rapportées : ____/____/_____ (J, M, A)

Statut Final du Patient: ☐ Vivant/Guéri ☐ Décédé

Si le patient est sorti guéri de l'hôpital :

Nom de l'hôpital : ____________________ Préfecture : ____________________

Si le patient était en isolement dans un CTE, Date de sortie de la zone d'isolement : ____/____/____ (J, M, A)

Date de sortie de l'hôpital : ____/____/______ (J, M, A)

Si le patient est décédé :

Date du Décès : ____/____/_____ (J, M, A)

Lieu du Décès : ☐ Communautaire ☐ Hôpital : ____________________ Préfecture : ____________________

Date des Funérailles : __/__/__ (J, M, A) Funérailles organisées par : ☐Famille/communautaire ☐Equipe d'enterrement

Lieu des funérailles: Préfecture: _____________ Sous-Préfecture : ______________ Village/Ville: ____________
